# Supplementary material for: Neutralizing and binding antibodies are a correlate of risk of COVID-19 in the CoVPN 3008 study in people with HIV
Source: Nat Commun. 2025 Oct 6;16:8876. doi: 10.1038/s41467-025-63948-4 (PMC12501024; doi:10.1038/s41467-025-63948-4)

CoVPN 3008 Study Team

First name	Last name	Organization
Sharlaa	Badal-Faesen	Clinical HIV Research Unit / Helen Joseph CRS
Kagisho	Baepanye	Hutchinson Centre Research Institute of South Africa
Veronique	Bailey	Hutchinson Centre Research Institute of South Africa
Katekani	Baloyi-Oseh	Hutchinson Centre Research Institute of South Africa
Mumtaz	Booley	Hutchinson Centre Research Institute of South Africa
Johannes Louis	Botha	TASK Eden
Yolande	Brown	Hutchinson Centre Research Institute of South Africa
Valerie	Brown	The Emmes Company, LLC
Lisa	Bunts	Fred Hutchinson Cancer Center
Soritha	Coetzer	Synexus Helderberg
Myron	Cohen	University of North Carolina at Chapel Hill
Shirley	Collie	BioInformatiCo
Rodney	Dawson	University of Cape Town Lung Institute CRS
Pallabi	Deb	Fred Hutchinson Cancer Center
Hana	El Sahly	Baylor College of Medicine
Jill	El-Khorazaty	The Emmes Company, LLC
Andries	Engelbrecht	Hutchinson Centre Research Institute of South Africa
Marianne	Gildea	FHI 360
Dhevium	Govender	Hutchinson Centre Research Institute of South Africa
Jen	Hanke	Fred Hutchinson Cancer Center
Jayla	Harris	Fred Hutchinson Cancer Center
Simone	Hendricks	Hutchinson Centre Research Institute of South Africa
Nick	Hopkinson	Fred Hutchinson Cancer Center
Haley	Howell	The Emmes Company, LLC
Nzeera	Ketter	Fred Hutchinson Cancer Center
Kentse	Khuto	Hutchinson Centre Research Institute of South Africa
Faatima	Laher Omar	Hutchinson Centre Research Institute of South Africa
Leolin	Katsidzira	University of Zimbabwe
Kim	Linton	The Emmes Company, LLC
James	Ludwig	Fred Hutchinson Cancer Center
Bongile	Mabilane	Hutchinson Centre Research Institute of South Africa
Matshidiso	Malefo	Hutchinson Centre Research Institute of South Africa
Ndiitwani	Mamushiana	Ndlovu Research Centre
Daciana	Margineantu	Fred Hutchinson Cancer Center
Jeanine	May	The Emmes Company, LLC
Fatima	Mayat	Perinatal HIV Research Unit
Cindy	Molitor	Fred Hutchinson Cancer Center

Yeshnee	Naidoo	KwaZulu-Natal Research Innovation and Sequencing Platform
Michelle	Nebergall	Fred Hutchinson Cancer Center
Alan	Nguyen	Fred Hutchinson Cancer Center
Sarah	Nikles	The Emmes Company, LLC
Bianca	Noronha	The Emmes Company, LLC
Melissa	Peda	Fred Hutchinson Cancer Center
Tamara	Phiri	Queen Elizabeth Central Hospital, Malawi
Shanthie	Pillay	Hutchinson Centre Research Institute of South Africa
Sureshnee	Pillay	KwaZulu-Natal Research Innovation and Sequencing Platform
Lori	Proulx-Burns	Fred Hutchinson Cancer Center
Laurie	Rinn	Fred Hutchinson Cancer Center
Lisa	Sanders	Fred Hutchinson Cancer Center
Carrie	Sopher	Fred Hutchinson Cancer Center
Smitha	Sripathy	Fred Hutchinson Cancer Center
Michael	Stirewalt	Fred Hutchinson Cancer Center
Houriiyah	Tegally	KwaZulu-Natal Research Innovation and Sequencing Platform
Sara	Thiebaud	Fred Hutchinson Cancer Center
Alicia	Toledano	The Emmes Company, LLC
Stephanie	Van Wyk	Centre for Epidemic Response & Innovation
Shamaya	Whitby	Fred Hutchinson Cancer Center
Stephany	Wilcox	Hutchinson Centre Research Institute of South Africa
Eduan	Wilkinson	Centre for Epidemic Response & Innovation
Haven	Wilvich	Fred Hutchinson Cancer Center
Charles	Wiysonge	South African Medical Research Council
Nelisiwe	Xaba	Hutchinson Centre Research Institute of South Africa
Ntokozo	Xulu	Hutchinson Centre Research Institute of South Africa

We also thank Judith Lucas and Tara McNair for GCLP Operations, Duke University.

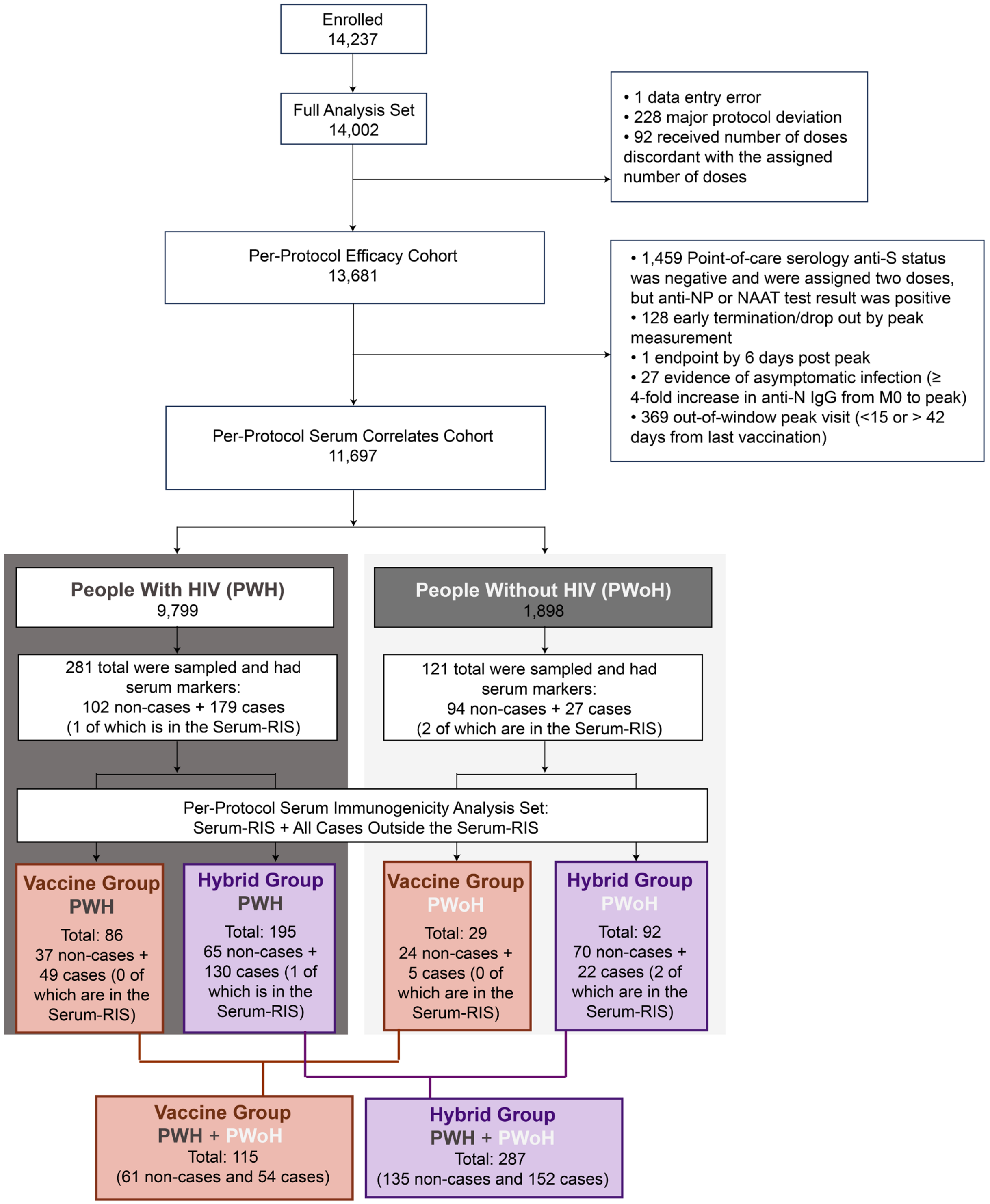

Supplementary Figure 1. Flowchart and numbers of participants in the Per-protocol Serum Immunogenicity Analysis Set, with antibody marker data available at both M0 and Peak and hence eligible for immune correlates analysis. RIS, Random Immunogenicity Subset.

Supplementary Table 1. Demographics of Per-protocol Serum Random Immunogenicity Subset participants in Key Pre-specified Subgroups and Overall.

Characteristic	Hybrid PWH, N = 66	Hybrid PWoH, N = 72	Vaccine PWH, N = 37	Vaccine PWoH, N = 24	Total, N = 199
Received vaccine doses as assigned	66 (100.0%)	72 (100.0%)	37 (100.0%)	24 (100.0%)	199 (100.0%)
SARS-CoV-2 NAAT result - N (%)					
Negative	64 (97.0%)	71 (98.6%)	37 (100.0%)	24 (100.0%)	196 (98.5%)
Positive	2 (3.0%)	1 (1.4%)	0 (0.0%)	0 (0.0%)	3 (1.5%)
Missing	0 (0.0%)	0 (0.0%)	0 (0.0%)	0 (0.0%)	0 (0.0%)
Central lab anti-N test result - N (%)					
Negative	19 (28.8%)	22 (30.6%)	37 (100.0%)	24 (100.0%)	102 (51.3%)
Positive	47 (71.2%)	50 (69.4%)	0 (0.0%)	0 (0.0%)	97 (48.7%)
Missing	0 (0.0%)	0 (0.0%)	0 (0.0%)	0 (0.0%)	0 (0.0%)
Point-of-care anti-Spike result – N (%)					
Negative	0 (0.0%)	0 (0.0%)	37 (100.0%)	24 (100.0%)	61 (100.0%)
Positive	66 (100.0%)	72 (100.0%)	0 (0.0%)	0 (0.0%)	138 (100.0%)
Missing	0 (0.0%)	0 (0.0%)	0 (0.0%)	0 (0.0%)	0 (0.0%)
Country					
Botswana	1 (1.5%)	2 (2.8%)	1 (2.7%)	2 (8.3%)	6 (3.0%)
Kenya	2 (3.0%)	2 (2.8%)	1 (2.7%)	0 (0.0%)	5 (2.5%)
South Africa	51 (77.3%)	59 (81.9%)	25 (67.6%)	21 (87.5%)	156 (78.4%)
Uganda	8 (12.1%)	6 (8.3%)	6 (16.2%)	1 (4.2%)	21 (10.6%)
Zambia	4 (6.1%)	3 (4.2%)	4 (10.8%)	0 (0.0%)	11 (5.5%)
Sex - N (%)					
Male	14 (21.2%)	35 (48.6%)	16 (43.2%)	20 (83.3%)	85 (42.7%)
Female	52 (78.8%)	37 (51.4%)	21 (56.8%)	4 (16.7%)	114 (57.3%)
Sex, BMI ≤25 - N (%)					
Male, ≤25	11 (78.6%)	25 (71.4%)	11 (68.8%)	19 (95.0%)	66 (77.6%)
Male, >25	3 (21.4%)	10 (28.6%)	5 (31.3%)	1 (5.0%)	19 (22.4%)
Female, ≤25	14 (26.9%)	5 (13.5%)	11 (52.4%)	2 (50.0%)	32 (28.1%)
Female, >25	38 (73.1%)	32 (86.5%)	10 (47.6%)	2 (50.0%)	82 (71.9%)
Race - N (%)					
Asian	0 (0.0%)	1 (1.4%)	0 (0.0%)	0 (0.0%)	1 (0.5%)
Black or African American	63 (95.5%)	69 (95.8%)	35 (94.6%)	21 (87.5%)	188 (94.5%)
Multiple	3 (4.5%)	2 (2.8%)	2 (5.4%)	3 (12.5%)	10 (5.0%)
Median age (range) - years	40.0 (23.0, 64.0)	38.5 (19.0, 67.0)	43.0 (20.0, 59.0)	36.0 (21.0, 65.0)	40.0 (19.0, 67.0)
Age category - N (%)					
≤40 years	34 (51.5%)	37 (51.4%)	16 (43.2%)	13 (54.2%)	100 (50.3%)
>40 years	32 (48.5%)	35 (48.6%)	21 (56.8%)	11 (45.8%)	99 (49.7%)
Active tuberculosis					
Yes	0 (0.0%)	0 (0.0%)	0 (0.0%)	0 (0.0%)	0 (0.0%)
No	65 (98.5%)	69 (95.8%)	37 (100.0%)	24 (100.0%)	195 (98.0%)
Missing	1 (1.5%)	3 (4.2%)	0 (0.0%)	0 (0.0%)	4 (2.0%)
History of tuberculosis					
Yes	11 (16.7%)	3 (4.2%)	4 (10.8%)	1 (4.2%)	19 (9.5%)
No	54 (81.8%)	66 (91.7%)	33 (89.2%)	23 (95.8%)	176 (88.4%)
Missing	1 (1.5%)	3 (4.2%)	0 (0.0%)	0 (0.0%)	4 (2.0%)

Characteristic	Hybrid PWH, N = 66	Hybrid PWoH, N = 72	Vaccine PWH, N = 37	Vaccine PWoH, N = 24	Total, N = 199
CD4 count** (cells/mm3) - N (%)					
<200	4 (6.1%)	0 (0.0%)	8 (21.6%)	0 (0.0%)	12 (6.0%)
200 - <350	5 (7.6%)	0 (0.0%)	3 (8.1%)	0 (0.0%)	8 (4.0%)
350 - <500	6 (9.1%)	0 (0.0%)	3 (8.1%)	0 (0.0%)	9 (4.5%)
≥500	47 (71.2%)	3 (4.2%)	21 (56.8%)	0 (0.0%)	71 (35.7%)
Missing	4 (6.1%)	69 (95.8%)	2 (5.4%)	24 (100.0%)	99 (49.7%)
Median CD4 count** (IQR) - cells/mm3	656.0 (508.8, 927.5)	789.0 (708.5, 964.5)	547.0 (270.0, 846.0)	NA (NA, NA)	645.0 (459.5, 913.0)
HIV viral load** (copies/mL) - N (%)					
<50	52 (78.8%)	3 (4.2%)	24 (64.9%)	0 (0.0%)	79 (39.7%)
≥50	10 (15.2%)	0 (0.0%)	10 (27.0%)	0 (0.0%)	20 (10.1%)
Missing	4 (6.1%)	69 (95.8%)	3 (8.1%)	24 (100.0%)	100 (50.3%)
Median HIV-1 viral load** (IQR) - copies/mL	76.5 (44.5, 234.8)	NA (NA, NA)	60.0 (40.0, 28,872.0)	NA (NA, NA)	60.0 (40.0, 19,599.0)
ART status**					
On ART	57 (86.4%)	0 (0.0%)	33 (89.2%)	0 (0.0%)	90 (45.2%)
Not on ART	9 (13.6%)	72 (100.0%)	4 (10.8%)	24 (100.0%)	109 (54.8%)
Missing	0 (0.0%)	0 (0.0%)	0 (0.0%)	0 (0.0%)	0 (0.0%)

** Only measured among PWH. The denominator for calculating percentages in the 'Total' column is the number of participants in study groups 1 and 2. PWH, people with HIV.

Supplementary Table 2. Comparison of estimated covariate-adjusted $\log_{10}$-scale antibody responses between A) Hybrid Group vs. Vaccine Group at M0, B) Hybrid PWH vs Hybrid PWoH at M0, C) Hybrid Group vs. Vaccine Group at Peak; D) Hybrid PWH vs. Hybrid PWoH at Peak; E) Vaccine PWH vs. Vaccine PWoH at Peak. The cohorts are random samples from the four groups, comprising the Serum-Random Immunogenicity Subset (Serum-RIS). P-values are two-sided and based on nonparametric bootstrap (see Methods). The random seed is set to 123 via R function set.seed(123).

A. Hybrid vs. Vaccine Group at M0 (adjusting for baseline factors HIV status, Age, Sex, BMI)

Marker ($\log_{10}$-scale)	Hybrid (95% CI)	Vaccine (95% CI)	Difference (95% CI)	P-value
M0 IgG N Index	4.72 (4.59, 4.84)	3.78 (3.63, 3.93)	0.94 (0.74, 1.13)	<0.001
M0 IgG Spike BA.5	4.24 (4.15, 4.33)	2.68 (2.54, 2.82)	1.56 (1.39, 1.74)	<0.001
M0 nAb-ID50 BA.4/5	2.54 (2.43, 2.67)	0.95 (0.84, 1.08)	1.59 (1.42,1.76)	<0.001

B. Hybrid PWH vs. Hybrid PWoH at M0 (adjusting for baseline factors Age, Sex, BMI)

Marker ($\log_{10}$-scale)	Hybrid PWH (95% CI)	Hybrid PWoH (95% CI)	Diff (95% CI)	P-value
M0 IgG N Index	4.72 (4.52, 4.91)	4.69 (4.51, 4.85)	0.03 (-0.22, 0.29)	0.779
M0 IgG Spike BA.5	4.17 (4.03, 4.32)	4.26 (4.09, 4.42)	-0.08 (-0.32, 0.14)	0.461
M0 nAb-ID50 BA.4/5	2.51 (2.34, 2.67)	2.57 (2.38, 2.77)	-0.06 (-0.33, 0.20)	0.700

C. Hybrid vs. Vaccine Group at Peak (adjusting for baseline factors HIV status, Age, Sex, BMI)

Marker ($\log_{10}$-scale)	Hybrid (95% CI)	Vaccine (95% CI)	Diff (95% CI)	P-value
Peak IgG Spike BA.5	5.31 (5.25, 5.38)	5.10 (5.00, 5.20)	0.21 (0.10, 0.33)	<0.001
Peak nAb-ID50 BA.4/5	3.69 (3.55, 3.83)	2.85 (2.66, 3.02)	0.84 (0.62, 1.07) (	<0.001

D. Hybrid PWH vs. Hybrid PWoH at Peak (adjusting for baseline factors Age, Sex, BMI)

Marker	Hybrid PWH (95% CI)	Hybrid PWoH (95% CI)	Diff (95% CI)	P-value
Peak IgG Spike BA.5	5.28 (5.20, 5.35)	5.37 (5.22, 5.49)	-0.09 (-0.24, 0.07)	0.277
Peak nAb-ID50 BA.4/5	3.74	3.70	0.04	0.803

	(3.50, 3.99)	(3.46, 3.92)	(-0.29, 0.37)	

E. Vaccine PWH vs. Vaccine PWoH at Peak (adjusting for baseline factors Age, Sex, BMI)

Marker	Vaccine PWH (95% CI)	Vaccine PWoH (95% CI)	Difference (95% CI)	P-value
Peak IgG Spike BA.5	5.12 (5.00, 5.22)	4.89 (4.81, 4.97)	0.23 (0.08, 0.36)	0.003
Peak nAb-ID50 BA.4/5	3.00 (2.74, 3.25)	2.51 (2.30, 2.73)	0.48 (0.17, 0.81)	0.005

nAb-ID50, 50% inhibitory serum dilution neutralizing antibody titer.

Hybrid Group PWH

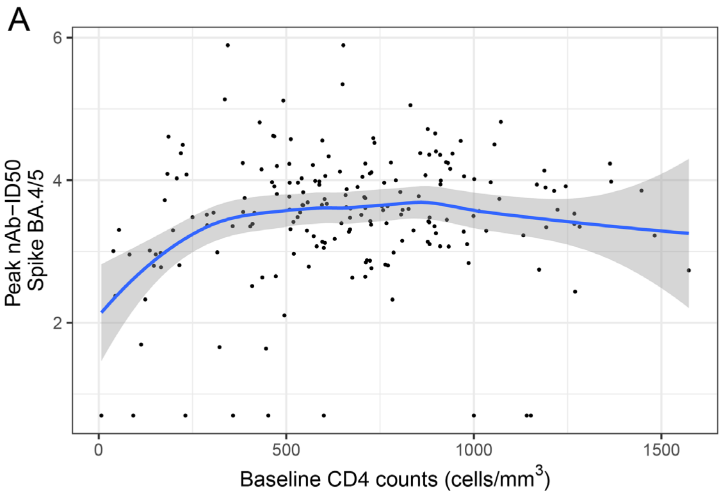

Vaccine Group PWH

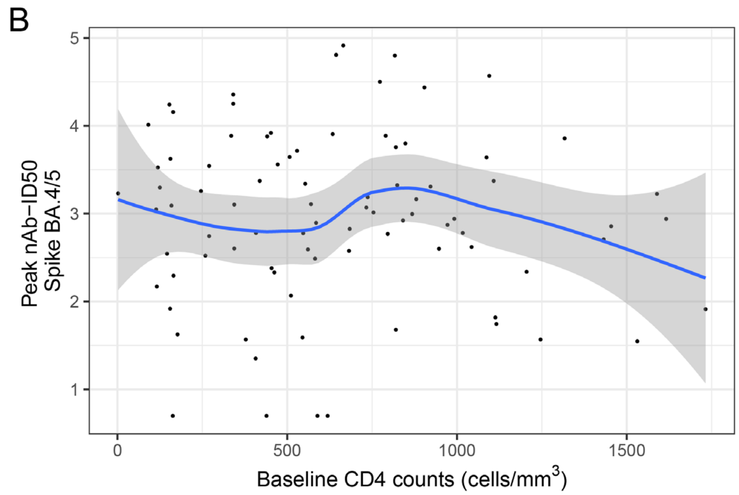

Supplementary Figure 2. Scatterplots of baseline CD4+ T-cell count and Peak nAb-ID50 BA.4/5 neutralizing antibody titer, including data from the 103 PWH participants in the Serum-Random Immunogenicity Subset, A) in the Hybrid Group (N=66) or B) in the Vaccine Group (N=37).

Supplementary Table 3. Two-sided Wald Interaction tests from covariate-adjusted Cox proportional hazards models of whether the hazard ratio of COVID-19 differs for the PWH subgroup compared to the people without HIV (PWoH) subgroup, with Holm-Bonferroni family-wise error rate (FWER) adjusted p-values. Data points are from eligible COVID-19 cases and non-cases in the Per-protocol Serum Immunogenicity Analysis Set. The Cox models use inverse probability sampling weighting. Analyses adjusted for whether enrolled in South Africa, HIV status, TB status, enrollment period (< 3 months, 3-6 months, > 6 months post first person enrolled), and baseline risk score. Markers at M0 are studied as correlates of risk only for the Hybrid Group, given that in the Vaccine Group the markers have very low variability at M0 because the participants are SAR-CoV-2 seronegative. The anti-N markers are only studied at M0 because the N protein is not included in the vaccine.
N, Nucleocapsid protein; nAb ID50, 50% inhibitory serum dilution neutralizing antibody titer; Pk = Peak time point [4 weeks post last vaccine dose (M1 for Hybrid Group, M2 for Vaccine Group)].

Antibody Marker	Hybrid Group Interaction P-value	FWER P-value	Vaccine Group Interaction P-value (FWER P-value)	FWER P-value
M0 IgG N Index (AU/ml)	P=0.011	0.077	NA	NA
M0 IgG Spike BA.4/5 (AU/ml)	P=0.660	1	NA	NA
M0 nAb ID50 BA.4/5 (AU/ml)	P=0.938	1	NA	NA
Peak IgG Spike BA.4/5 (AU/ml)	P=0.619	1	P=0.823	1
Peak nAb ID50 BA.4/5 (AU/ml)	P=0.179	1	P=0.551	1

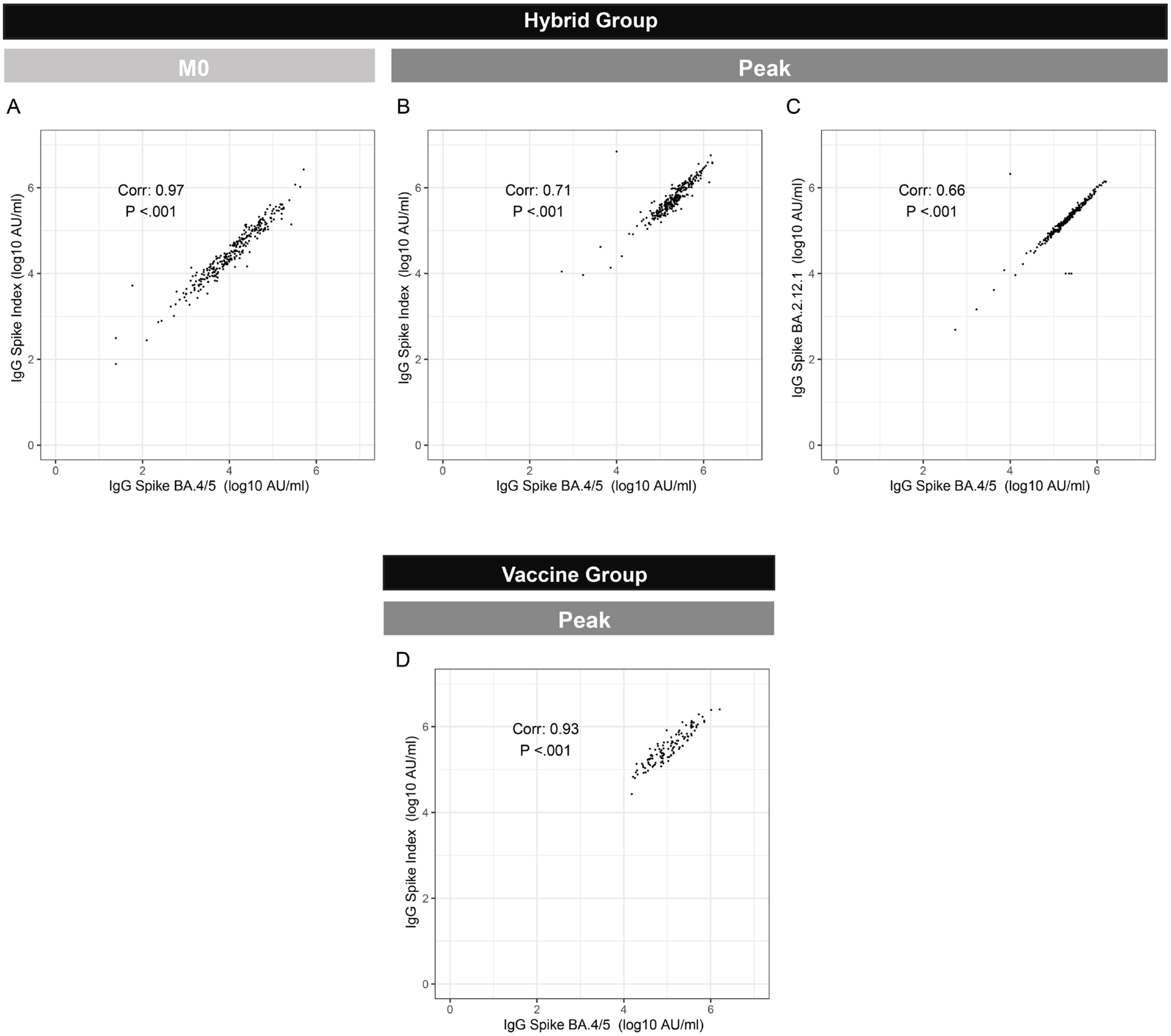

Supplementary Figure 3. Pairwise correlations of IgG Spike BA.4/5 and (A, B, D) IgG Spike Index or (C) IgG Spike BA.2.12.1 at (A) M0 or (B, C, D) at Peak, in (A, B, C) the Hybrid Group or (D) the Vaccine Group. Data points are from eligible COVID-19 cases and non-cases in the Per-protocol Serum Immunogenicity Analysis Set.

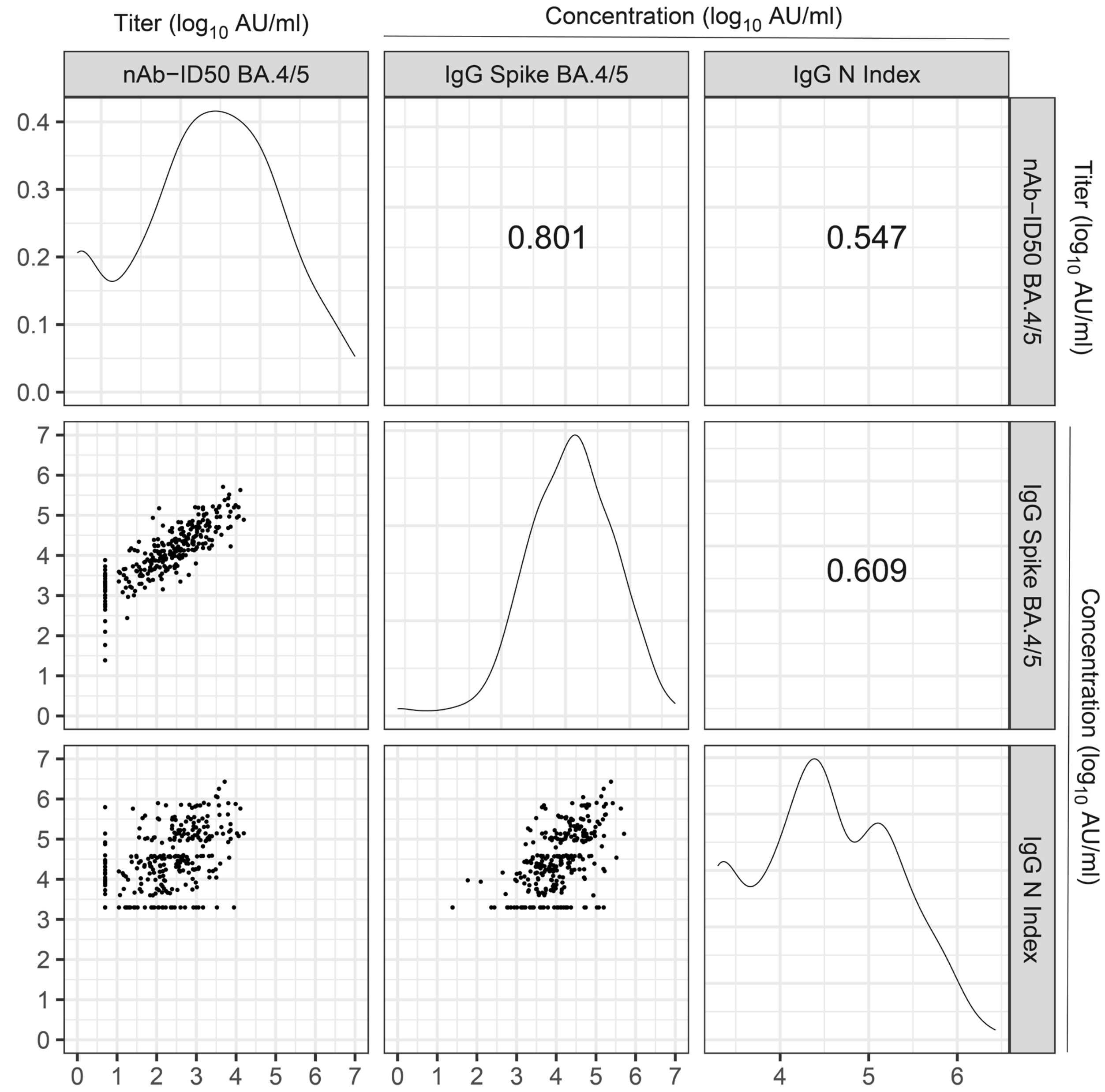

Supplementary Figure 4. Pairwise correlations of the antibody markers evaluated as correlates at M0 in the Hybrid Group. Data points are from eligible COVID-19 cases and non-cases in the Per-protocol Serum Immunogenicity Analysis Set. P < 0.001 for all three pairwise correlations.

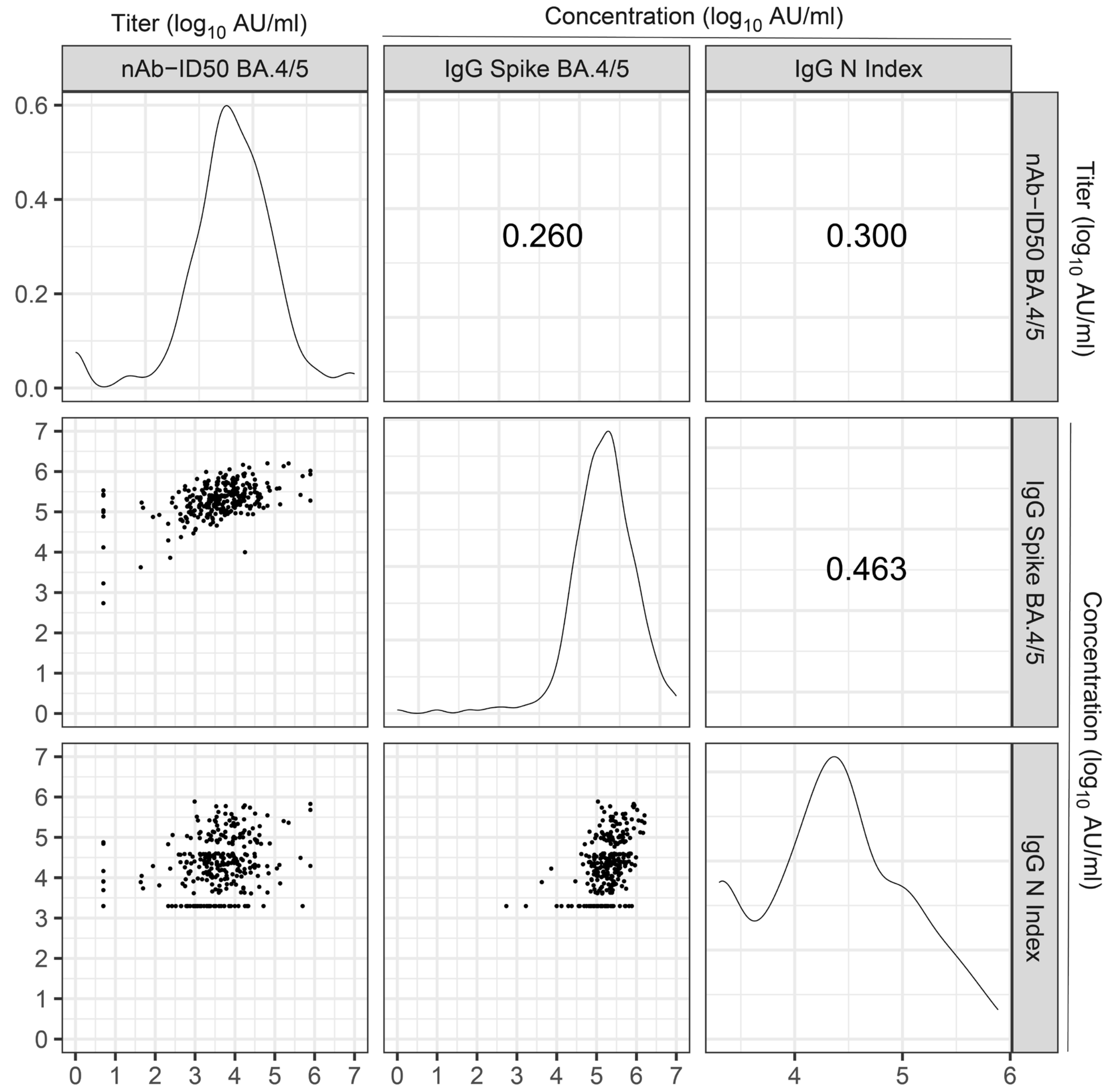

Supplementary Figure 5. Pairwise correlations of the antibody markers evaluated as correlates at Peak in the Hybrid Group. Data points are from eligible COVID-19 cases and non-cases in the Per-protocol Serum Immunogenicity Analysis Set. Peak, 4 weeks post last vaccine dose (M1 for Hybrid Group). P < 0.001 for all three pairwise correlations.

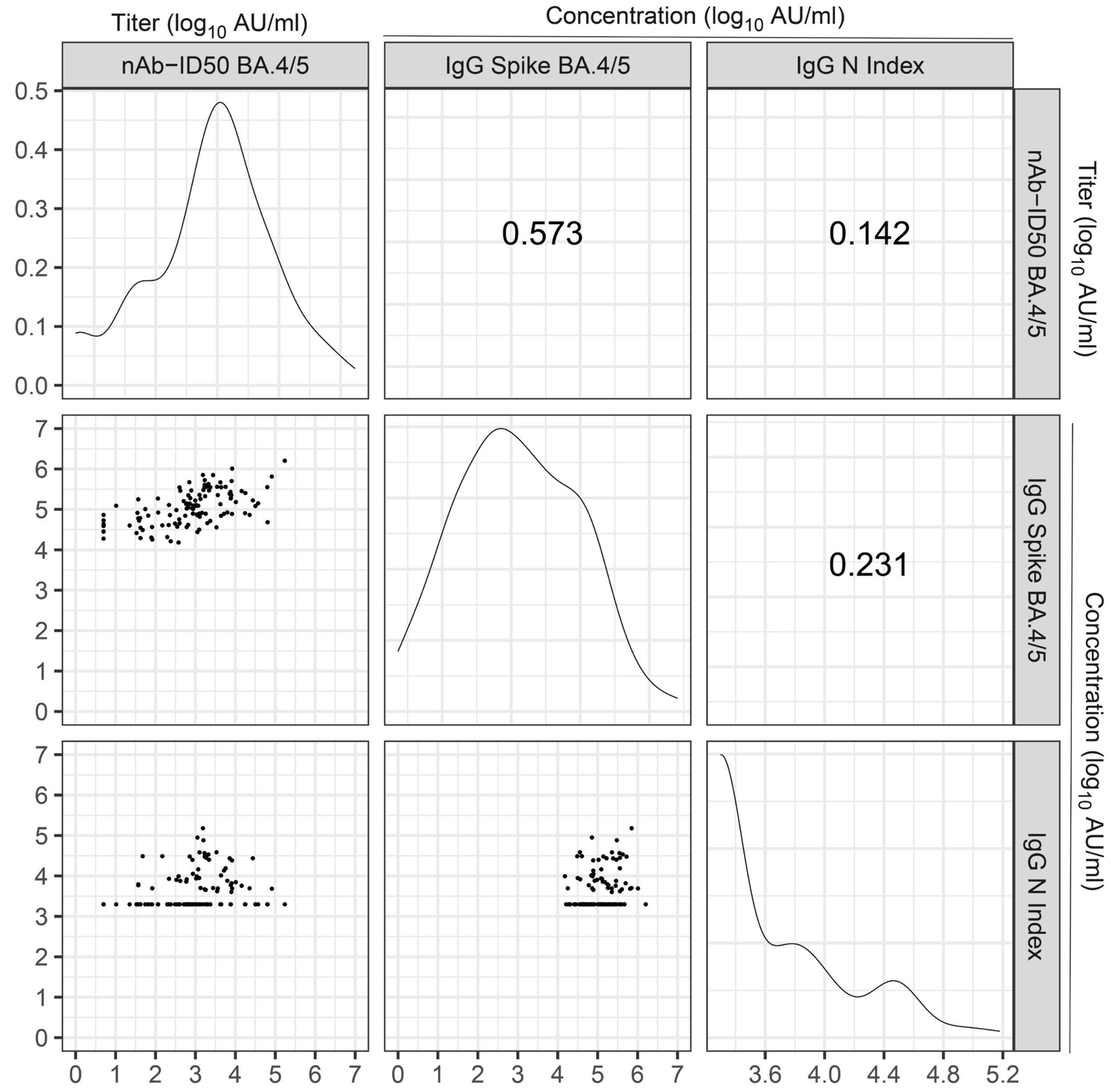

Supplementary Figure 6. Pairwise correlations of the antibody markers evaluated as correlates at Peak in the Vaccine Group. Data points are from eligible COVID-19 cases and non-cases in the Per-protocol Serum Immunogenicity Analysis Set. Peak, 4 weeks post last vaccine dose (M2 for Vaccine Group). P < 0.001 (IgG Spike vs nAb), P = 0.13 (IgG N vs nAb), and P = 0.013 (IgG Spike vs IgG N).

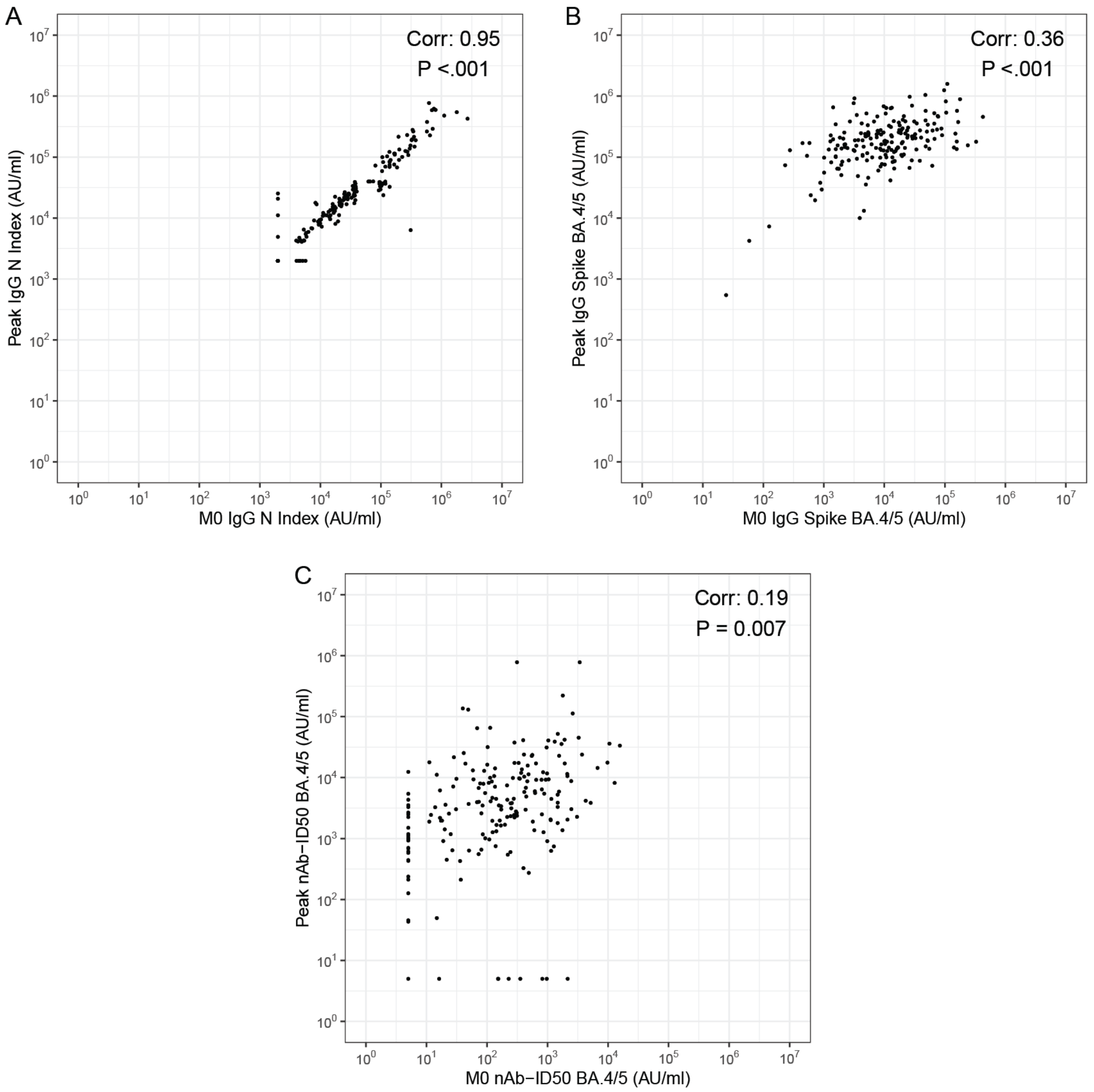

Supplementary Figure 7. Pairwise correlations of each of the antibody markers evaluated as correlates at M0 and Peak, for the Hybrid Group. (A) M0 IgG N Index x Peak IgG N Index, (B) M0 IgG Spike BA.4/5 x Peak IgG Spike BA.4/5, (C) M0 nAb-ID50 BA.4/5 x Peak nAb-ID50 BA.4/5. Data points are from eligible COVID-19 cases and non-cases in the Per-protocol Serum Immunogenicity Analysis Set. Peak, 4 weeks post last vaccine dose (M1 for Hybrid Group).

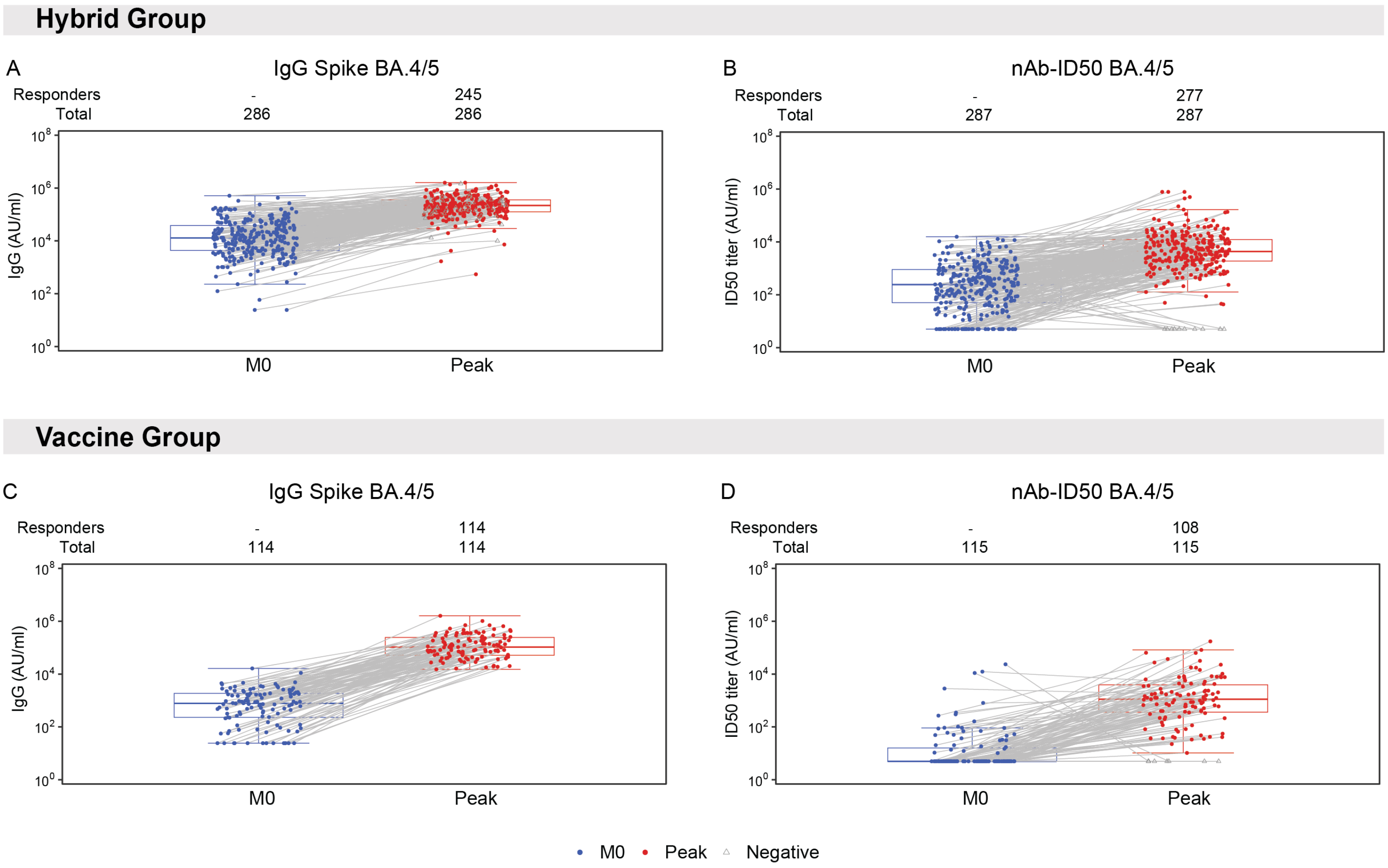

Supplementary Figure 8. Spaghetti plots connecting M0 and Peak antibody marker values for (A) Hybrid Group IgG Spike BA.4/5 concentration, (B) Hybrid Group nAb-ID50 BA.4/5 titer, (C) Vaccine Group IgG Spike BA.4/5 concentration, (D) Vaccine Group nAb-ID50 BA.4/5 titer. Peak, 4 weeks post last vaccine dose (M1 for Hybrid Group, M2 for Vaccine Group). Data points are from eligible COVID-19 cases and non-cases in the Per-protocol Serum Random Immunogenicity Subset. nAb-ID50, 50% inhibitory serum dilution neutralizing antibody titer.

Hybrid Group

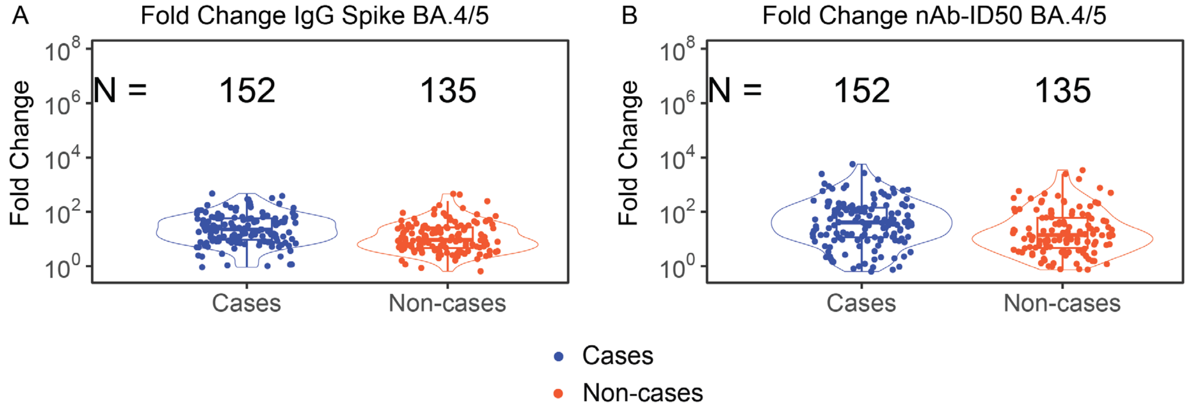

Supplementary Figure 9. Antibody marker fold-change (Peak/M0) of A) IgG Spike BA.4/5 concentration and B) nAb-ID50 BA.4/5 titer by COVID-19 outcome status for the Hybrid Group. Data points are from eligible COVID-19 cases (COVID-19 7-230 days post Peak) and non-cases in the Per-protocol Serum Immunogenicity Analysis Set. The violin plots contain interior box plots with upper and lower horizontal edges representing the 25th and 75th percentiles of antibody level and middle line representing the 50th percentile. The vertical bars represent the distance from the 25th (or 75th) percentile of antibody level and the minimum (or maximum) antibody level within the 25th (or 75th) percentile of antibody level minus (or plus) 1.5 times the interquartile range. Each side shows a rotated probability density (estimated by a kernel density estimator with a default Gaussian kernel) of the data. Cases acquired a COVID-19 endpoint 7 days post Peak through 230 days post Peak. Non-cases did not have a positive RT-PCR result at the Peak visit and did not acquire a COVID-19 endpoint after M0 up to the date by which the last enrolled participant reached 230 days post Peak (March 31, 2023). Peak, 4 weeks post last vaccine dose (M1 for Hybrid Group). nAb-ID50, 50% inhibitory serum dilution neutralizing antibody titer.

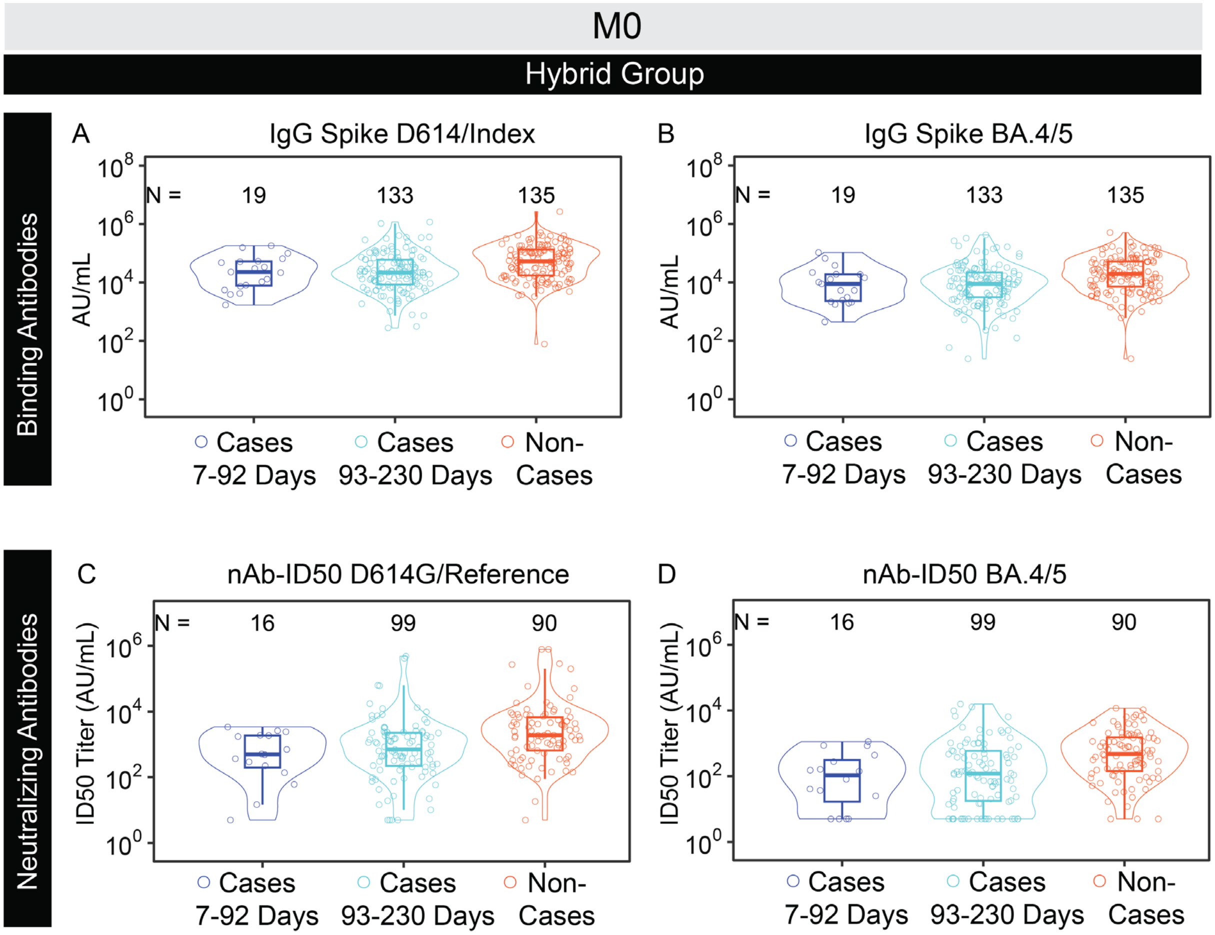

Supplementary Figure 10. Antibody marker levels at M0 in the Hybrid Group by COVID-19 outcome status. Data points are from eligible COVID-19 cases and non-cases in the Per-protocol Serum Immunogenicity Analysis Set. To compare the M0 immune responses against the vaccine strain (almost identical to D614/Index or D614G/Reference) compared to against the BA.5 circulating strain, the plots compare IgG Spike (A) D614/Index vs. (B) BA.4/5; nAb-ID50 (C) D614G/Reference vs. (D) BA.4/5. For non-cases, the GM values (in AU/ml) are (A) 49,384, (B) 18,477, (C) 2233, and (D) 367.8. The violin plots contain interior box plots with upper and lower horizontal edges representing the 25th and 75th percentiles of antibody level and middle line representing the 50th percentile. The vertical bars represent the distance from the 25th (or 75th) percentile of antibody level and the minimum (or maximum) antibody level within the 25th (or 75th) percentile of antibody level minus (or plus) 1.5 times the interquartile range. Each side shows a rotated probability density (estimated by a kernel density estimator with a default Gaussian kernel) of the data. Cases acquired a COVID-19 endpoint 7 days post Peak through 92 days post Peak or 93 days post Peak through 230 days post Peak, as designated in the key at the bottom of each panel. Non-cases did not have a positive RT-PCR result at the Peak visit and did not acquire a COVID-19 endpoint after M0 up to the date by which the last enrolled participant reached 230 days post Peak (March 31, 2023). N, Nucleocapsid protein; nAb-ID50, 50% inhibitory serum dilution neutralizing antibody titer.

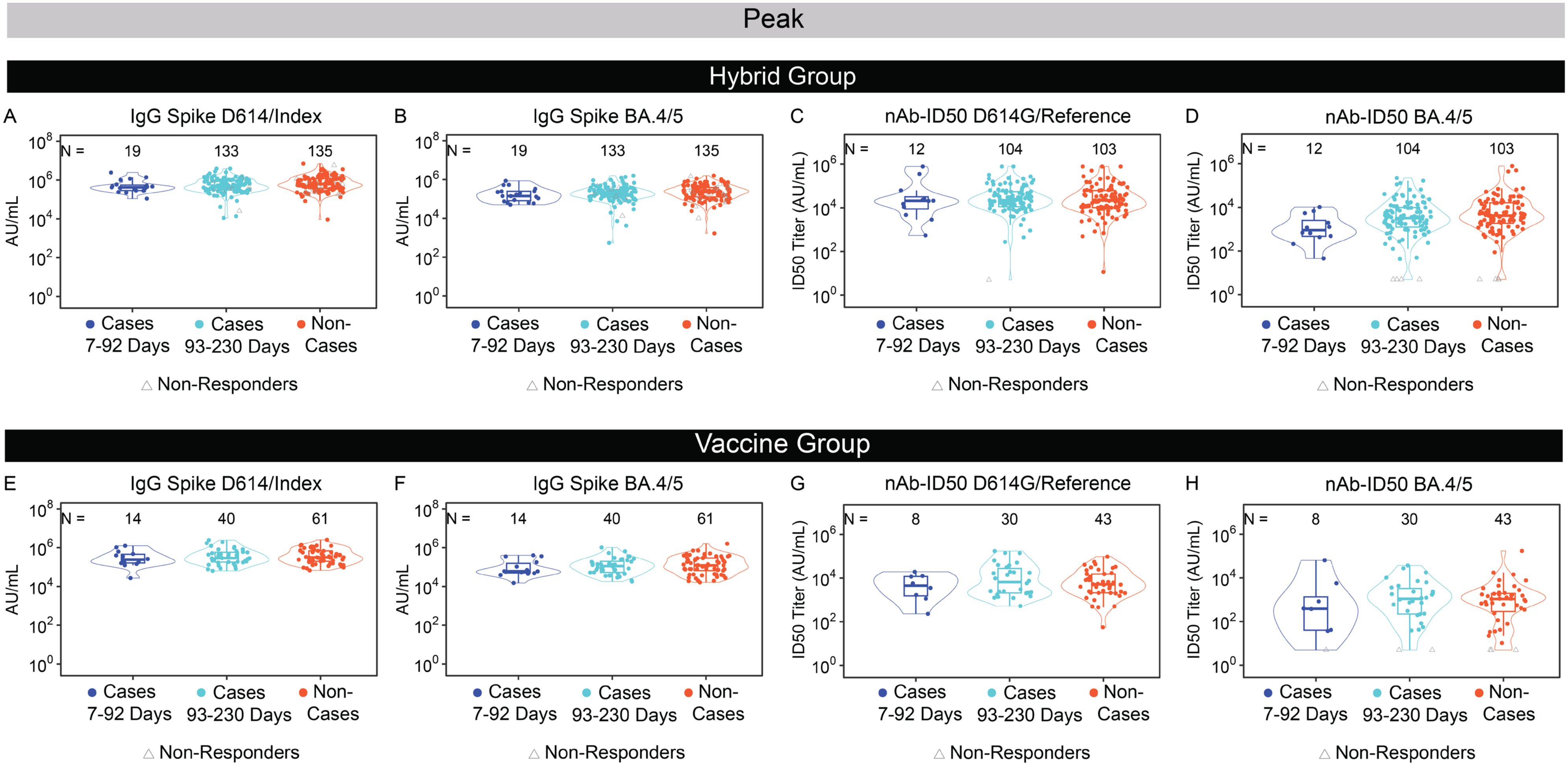

Supplementary Figure 11. Antibody marker levels at Peak in the Hybrid Group and the Vaccine Group by COVID-19 outcome status. Data points are from eligible COVID-19 cases and non-cases in the Per-protocol Serum Immunogenicity Analysis Set. To quantify drop off in antibody responses against the vaccine strain (almost identical to D614/Index for IgG Spike or D614G/Reference for nAb ID50) compared to against the BA.5 circulating strain, the plots compare IgG Spike (A, E) D614/Index vs. (B, F) BA.4/5; nAb-ID50 (C, G) D614G/Reference vs. (D, H) BA.4/5. (A-D) Hybrid Group, (E-H) Vaccine Group. Peak, 4 weeks post last vaccine dose (M1 for Hybrid Group, M2 for Vaccine Group). For non-cases, the GM values (in AU/ml) are: (A) 614,838, (B) 213,245 , (C) 21,786, (D) 4700, (E) 353,331, (F) 120,017, (G) 5279, and (H) 757. The violin plots contain interior box plots with upper and lower

horizontal edges representing the 25th and 75th percentiles of antibody level and middle line representing the 50th percentile. The vertical bars represent the distance from the 25th (or 75th) percentile of antibody level and the minimum (or maximum) antibody level within the 25th (or 75th) percentile of antibody level minus (or plus) 1.5 times the interquartile range. Each side shows a rotated probability density (estimated by a kernel density estimator with a default Gaussian kernel) of the data. Vaccine-response at Peak for each immune marker is defined in Table 1. Cases acquired a COVID-19 endpoint 7 days post Peak through 92 days post Peak or 93 days post Peak through 230 days post Peak, as designated in the key at the bottom of each panel. Non-cases did not have a positive RT-PCR result at the Peak visit and did not acquire a COVID-19 endpoint after M0 up to the date by which the last enrolled participant reached 230 days post Peak (March 31, 2023). Peak, 4 weeks post last vaccine dose (M1 for Hybrid Group, M2 for Vaccine Group); N, Nucleocapsid protein; nAb-ID50, 50% inhibitory serum dilution neutralizing antibody titer.

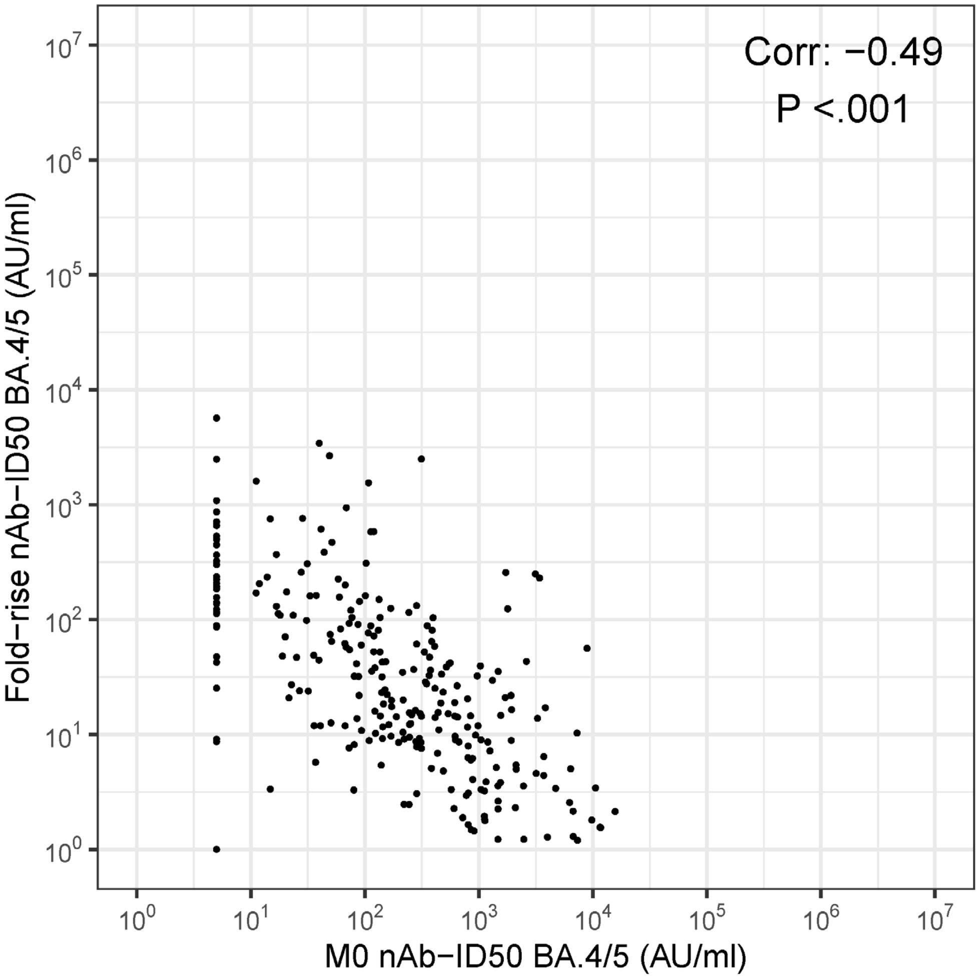

Supplementary Figure 12. Pairwise correlation of M0 nAb-ID50 BA.4/5 and IgG Spike Index at (A) M0 and (B, C) at Peak, in (A, B) the Hybrid Group and (C) the Vaccine Group. Data points are from eligible COVID-19 cases and non-cases in the Per-protocol Serum Immunogenicity Analysis Set.

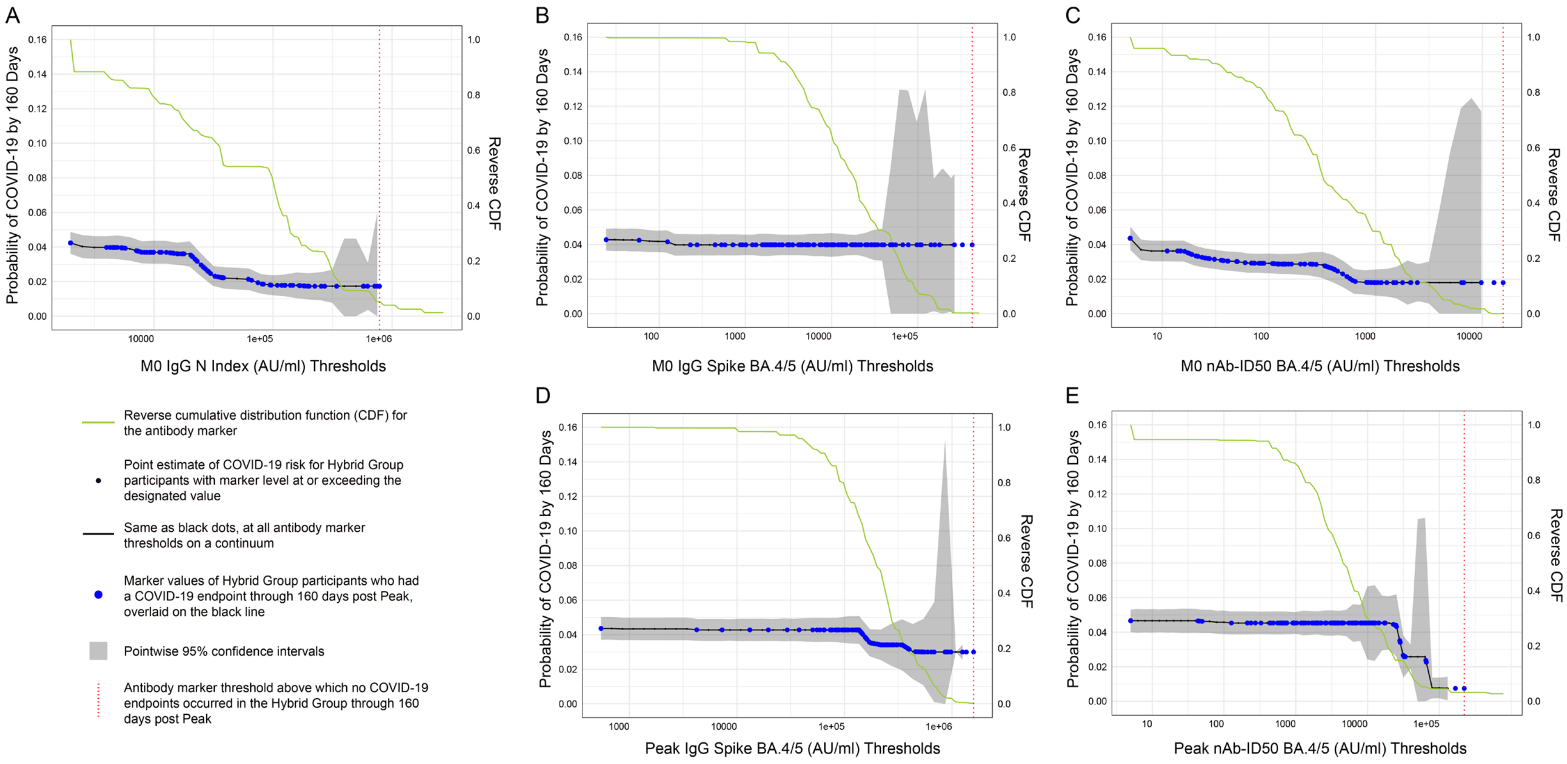

Supplementary Figure 13. Covariate-adjusted cumulative incidence of COVID-19 through 160 days post Peak for the Hybrid Group with thresholded antibody marker (A) M0 IgG N Index concentration, (B) M0 IgG Spike BA.4/5 concentration, (C) M0 nAb-ID50 BA.4/5 titer, (D) Peak IgG Spike BA.4/5 concentration, (E) Peak nAb-ID50 BA.4/5 titer. Each black dot (threshold value) represents a point estimate of COVID-19 risk through 160 days post-Peak for Hybrid Group participants if their marker levels were as high as or higher than that given threshold value. The grid of thresholds was created by segmenting the marker values at COVID-19 endpoints into increments of 0.1. This grid spans from the minimum marker value to the highest value for which there are at least 3 COVID-19 endpoints with a marker value at or above that value. The solid black lines linearly interpolate the grid points. The grey shaded area indicates pointwise 95% CIs. The estimates and CIs were adjusted using the assumption that the true threshold-response risk is non-increasing. The green curve is the estimate of the reverse cumulative distribution function (CDF) of the marker. The blue dots represent the marker values of Hybrid Group participants who had a COVID-19 endpoint through 160 days post Peak, overlaid on the black line. The vertical red dashed line is the antibody marker threshold above which no COVID-19 endpoints occurred in the Hybrid Group through 160 days post Peak. Analyses adjusted for whether enrolled in South Africa, HIV status, and baseline risk score. Data points are from eligible COVID-19 cases and non-cases in the Per-protocol Serum Immunogenicity Analysis Set. nAb-ID50, 50% inhibitory serum dilution neutralizing antibody titer; Peak, 4 weeks post last vaccine dose (M1 for Hybrid Group).

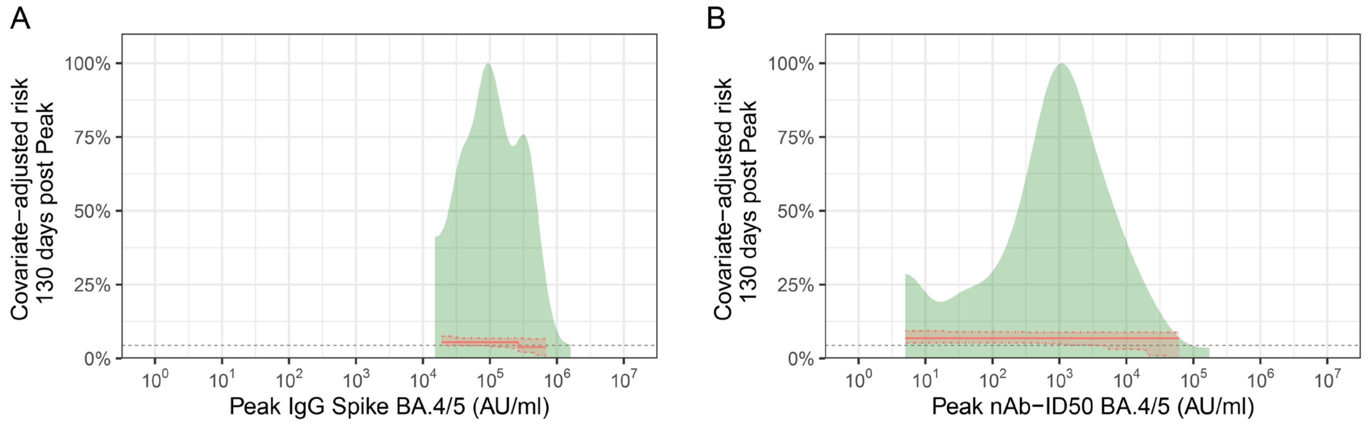

Supplementary Figure 14. Sensitivity analysis for the correlates of risk analyses in the Vaccine Group for antibodies measured at Peak. Data points are from eligible COVID-19 cases and non-cases in the Per-protocol Serum Immunogenicity Analysis Set in the Vaccine Group (N = 115). Controlled risk plots for Vaccine Group (N = 115) antibody markers at Peak, over follow-up through 130 days post Peak. The plots show the covariate-adjusted probability of COVID-19 by 130 days post Peak under hypothetical assignments of all participants to the Vaccine Group and a given marker value as indicated on the x-axis. (A) Peak IgG Spike BA.4/5 concentration, (B) Peak nAb ID50 BA.4/5 titer. Controlled risk was estimated using a monotone-constrained nonparametric method with covariate adjustment (default implementation in the R package *vaccine* available at CRAN[1]) and restricted to the middle 95% quantiles of the available marker data. Dotted lines and shading indicate pointwise 95% CIs. The horizontal gray lines are the overall cumulative incidence of COVID-19 from 7 to 92 days (or 165 days, as designated) post Peak in the Vaccine Group. The background kernel density plots are estimates of the distribution of the antibody marker at M0 or at Peak. Analyses adjusted for whether enrolled in South Africa, HIV status, TB status, enrolment period (< 3 months, 3-6 months, > 6 months post first person enrolled), and baseline risk score. AU, arbitrary units; FWER, family-wise error rate adjusted p-value; N, Nucleocapsid protein; nAb-ID50, 50% inhibitory serum dilution neutralizing antibody titer; Peak, 4 weeks post last vaccine dose (M2 for Vaccine Group).

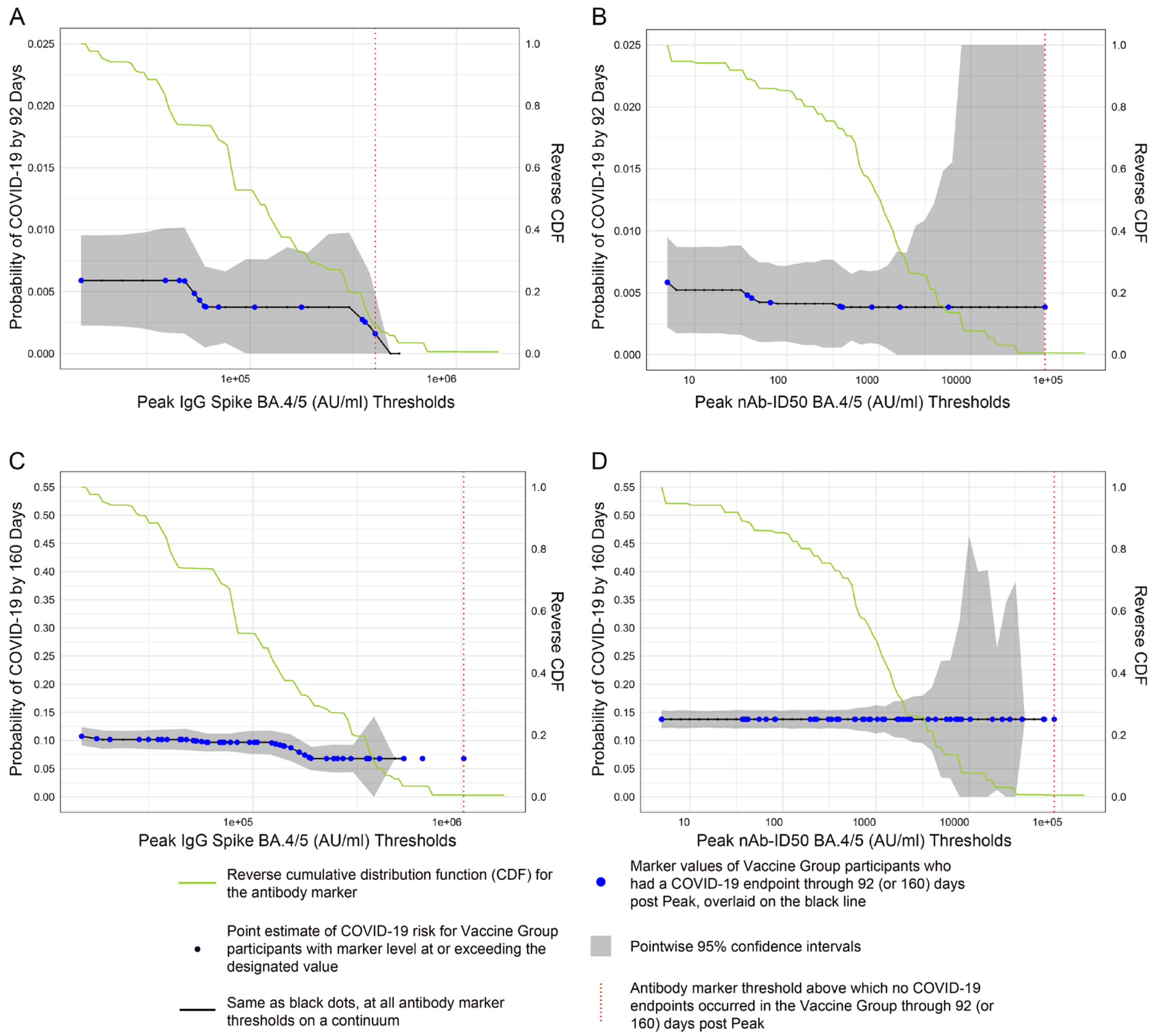

Supplementary Figure 15. Covariate-adjusted cumulative incidence of COVID-19 through (A, B) 92 days post Peak or (C, D) 160 days post Peak for the Vaccine Group with thresholded antibody marker (A, C) Peak IgG Spike BA.4/5 concentration and (B, D) Peak nAb-ID50 BA.4/5 titer. Each black dot (threshold value) represents a point estimate of COVID-19 risk through 92 (or 160) days post-Peak for Vaccine Group participants if their marker levels were as high as or higher than that given threshold value. The grid of thresholds was created by segmenting the marker values at COVID-19 endpoints into increments of 0.1. This grid spans from the minimum marker value to the highest value for which there are at least 3 COVID-19 endpoints with a marker value at or above that value. The solid black lines linearly interpolate the grid points. The grey shaded area indicates pointwise 95% CIs. The estimates and CIs were adjusted using the assumption that the true threshold-response risk is non-increasing. The green curve is the estimate of the reverse cumulative distribution function (CDF) of the marker. The blue dots represent the marker values of Vaccine Group participants who had a COVID-19 endpoint through the relevant follow-up (92 or 160 days), overlaid on the black line. The vertical red dashed line is the antibody marker threshold above which no COVID-19 endpoints occurred in the Vaccine Group through 92 (or 160) days post Peak. Analyses through 92 days post Peak (A, B) adjusted for baseline risk score only; analyses through 160 days post Peak (C, D) adjusted for whether enrolled in South Africa, HIV status, and baseline risk score; Data points are from eligible COVID-19 cases and non-cases in the Per-protocol Serum Immunogenicity Analysis Set. nAb-ID50, 50% inhibitory serum dilution neutralizing antibody titer; Peak, 4 weeks post last vaccine dose (M2 for Vaccine Group).

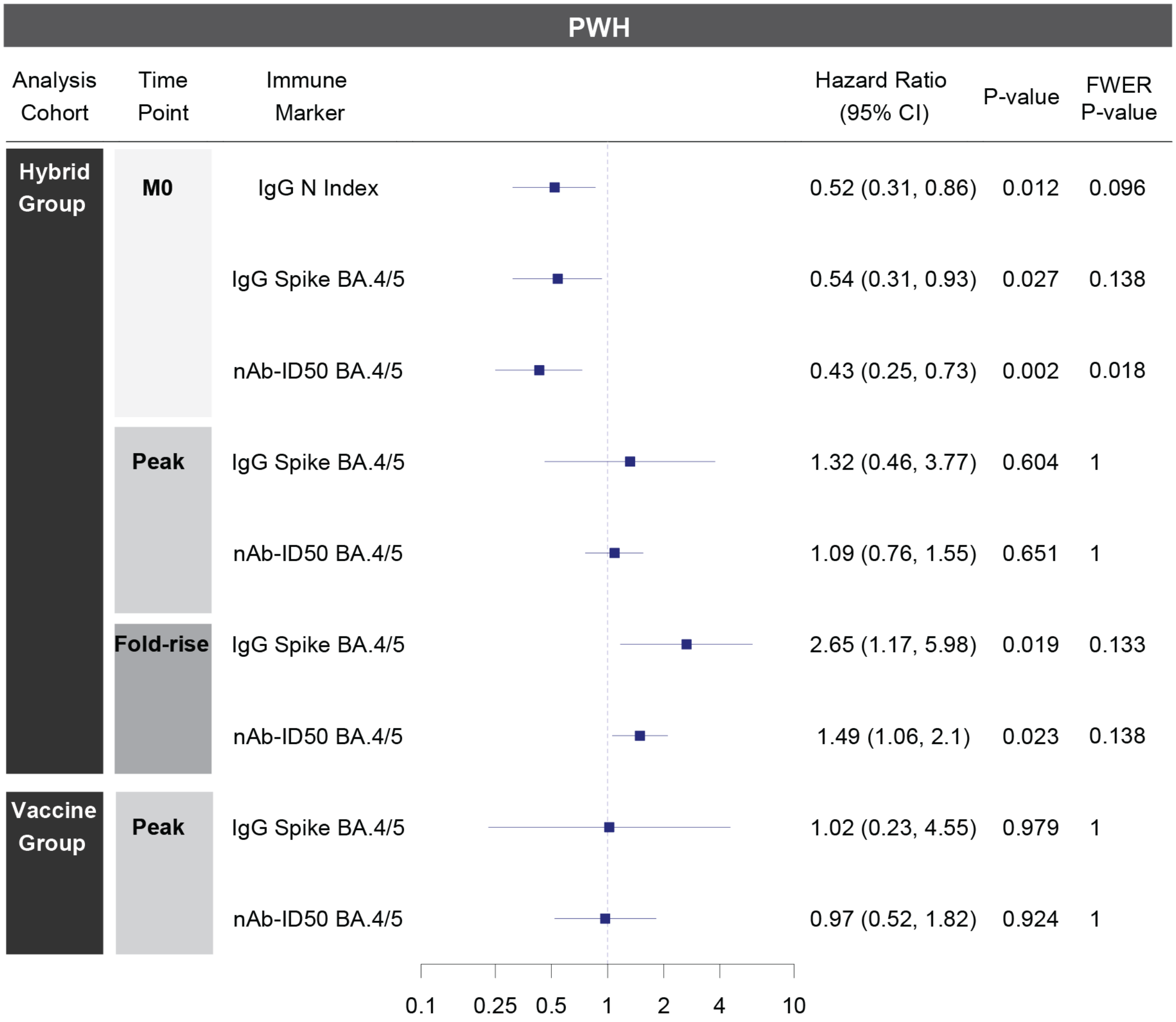

Supplementary Figure 16. Covariate-adjusted hazard ratios (HRs) of COVID-19 (7 to 230 days post Peak) per 10-fold increase in the designated immune marker at M0, at Peak, or for Peak/M0 fold-rise as indicated, for PWH. Data points are from eligible COVID-19 cases and non-cases in the Per-protocol Serum Immunogenicity Analysis Set, for the subgroup of people with HIV (PWH) (N = 195 in the Hybrid Group and N = 86 in the Vaccine Group). Cases acquired a COVID-19 endpoint 7 days post Peak through 230 days post Peak. Non-cases did not have a positive RT-PCR result at the Peak visit and did not acquire a COVID-19 endpoint after M0 up to the date by which the last enrolled participant reached 230 days post Peak (March 31, 2023). HRs were estimated using inverse probability sampling weighted Cox regression models; 95% confidence intervals (CIs) and Wald-based p-values are shown. Analyses of the antibody markers adjusted for whether enrolled in South Africa, HIV status, TB status, enrollment period (< 3 months, 3-6 months, > 6 months post first person enrolled), and baseline risk score. Markers against N at Peak are not studied as correlates because the mRNA-1273 vaccine did not contain N. FWER, family-wise error rate adjusted p-value; N, Nucleocapsid protein; nAb-ID50, 50% inhibitory serum dilution neutralizing antibody titer; Peak, 4 weeks post last vaccine dose (M1 for Hybrid Group, M2 for Vaccine Group).

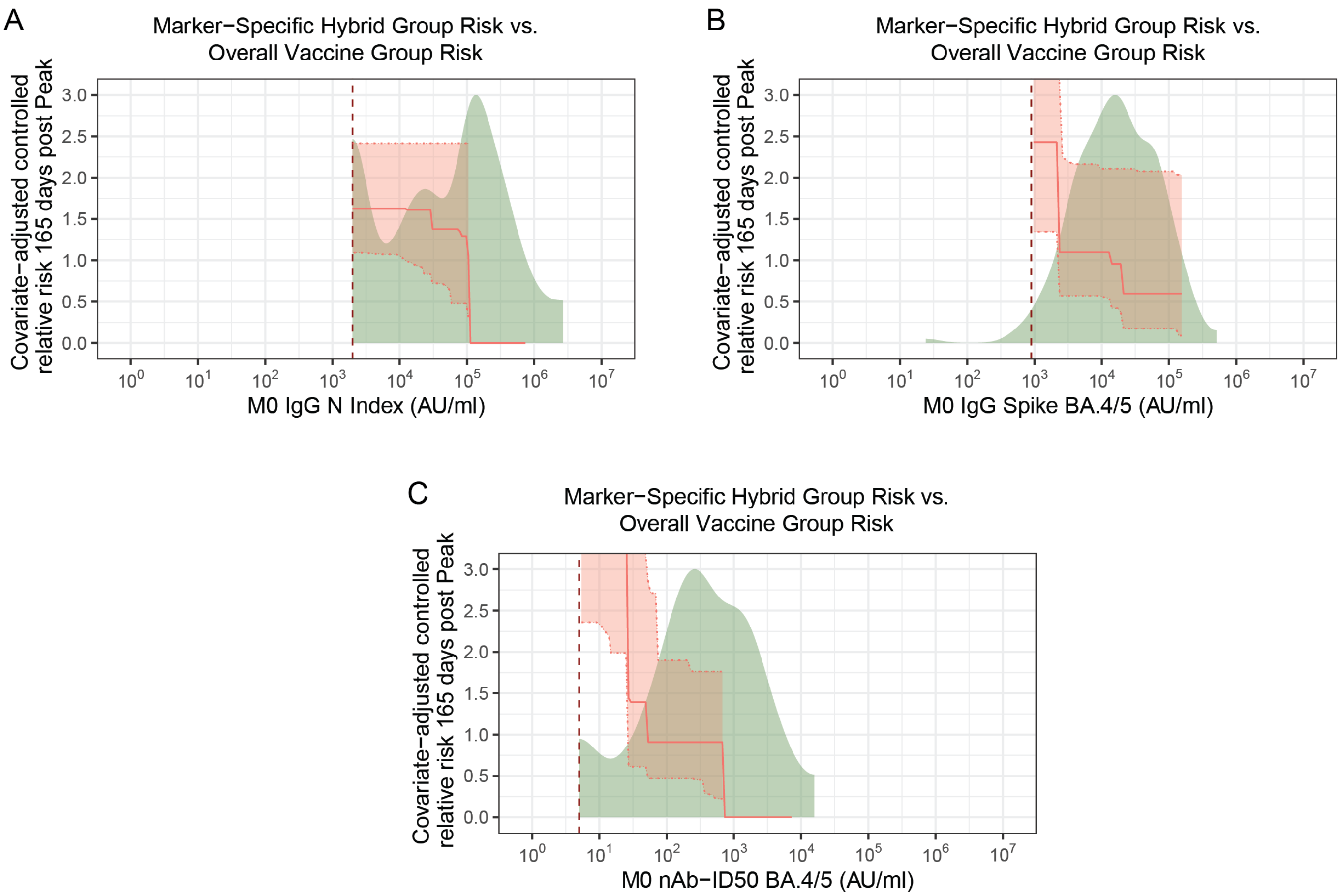

Supplementary Figure 17. Controlled relative risk of COVID-19 through 165 days post Peak for Hybrid Group (N = 287) antibody marker value at M0 vs. overall risk for the Vaccine Group (N = 115). Data points are from eligible COVID-19 cases and non-cases in the Per-protocol Serum Immunogenicity Analysis Set. (A) IgG N Index concentration, (B) IgG Spike BA.4/5 concentration, (C) nAb-ID50 BA.4/5 titer. Controlled risk by M0 marker value was estimated using a monotone-constrained nonparametric method with covariate adjustment,[2] and divided by the nonparametric estimate of the overall covariate-adjusted risk of the Vaccine Group to constitute the controlled relative risk. Dotted lines and shading indicate pointwise 95% CIs. The background kernel density plots are estimates of the distribution of each antibody marker. The dotted red line indicates the median marker level among the Vaccine Group. Analyses adjusted for whether enrolled in South Africa, HIV status, TB status, enrollment period (< 3 months, 3-6 months, > 6 months post first person enrolled), and baseline risk score. AU, arbitrary units; nAb-ID50, 50% inhibitory serum dilution neutralizing antibody titer; Peak, 4 weeks post last vaccine dose (M1 for Hybrid Group, M2 for Vaccine Group).

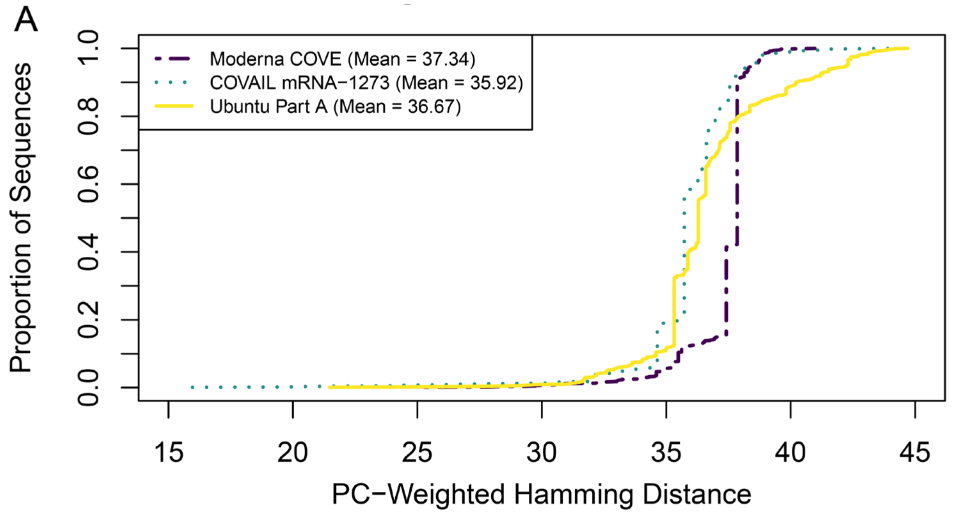

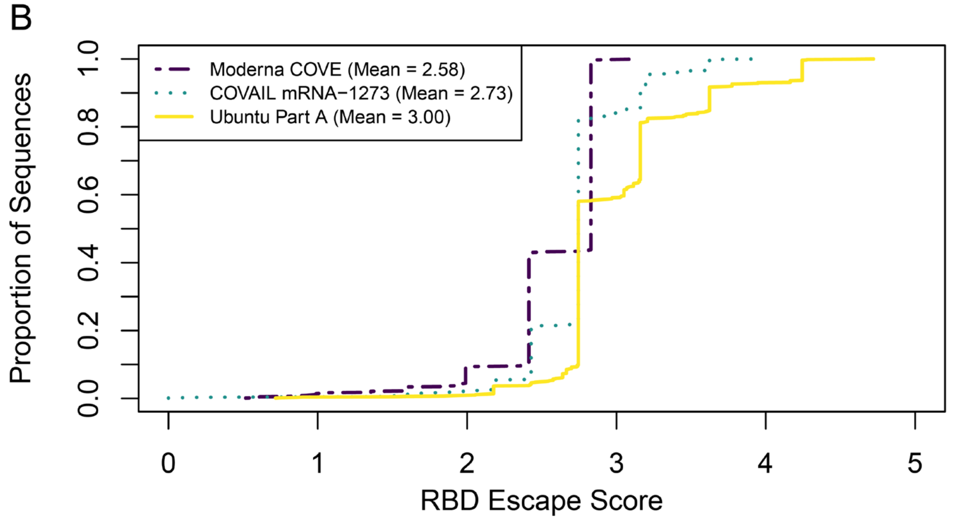

Supplementary Figure 18. Distributions of Spike amino acid sequence distances of estimated circulating strains to the mRNA-1273 ancestral vaccine strain in three immune correlates studies of mRNA-1273: Moderna COVE 3-dose mRNA-1273 recipients, COVAIL second-booster mRNA-1273 recipients, CoVPN 3008 Part A 2-dose mRNA-1273 recipients. (A) Spike physico-chemical weighted Hamming distances, as done in Magaret et al.,[3] scaled to reflect number of mismatches from the vaccine strain; (B) RBD antigenic escape distances where escape is from the mRNA-1273 vaccine strain.[4] Moderna COVE correlates study COVID-19 endpoints, all BA.1 lineage, occurred in the U.S. from December 1, 2021 to April 5, 2022: shown are distances from a random sample of 1000 complete BA.1 Spike sequences from GISAID U.S. entries sampled uniformly over the same range of dates. COVAIL correlates study mRNA-1273 vaccine arm COVID-19 endpoints occurred in the U.S. from May 30, 2022 to November 28, 2022: shown are distances from a random sample of 1000 complete Spike sequences from GISAID U.S. entries sampled uniformly over the same range of dates. CoVPN 3008 Part A correlates study COVID-19 endpoints including both the Vaccine and Hybrid groups occurred in Botswana, eSwatini, Kenya, Malawi, South Africa, Uganda, and Zambia from April 26, 2022 to February 7, 2023: shown are distances from a random sample of 1000 sequences from GISAID sampled uniformly over the same range of dates and proportionately stratified by country representation.

Supplementary Table 4.

Data Availability

GISAID Identifier: EPI_SET_241220es

doi: 10.55876/gis8.241220es

All genome sequences and associated metadata in this dataset are published in GISAID's EpiCoV database. To view the contributors of each individual sequence with details such as accession number, Virus name, Collection date, Originating Lab and Submitting Lab and the list of Authors, visit 10.55876/gis8.241220es

Data Snapshot

- EPI_SET_241220es is composed of 3,000 individual genome sequences.
- The collection dates range from 2021-12-07 to 2023-02-07;
- Data were collected in 8 countries and territories;
- All sequences in this dataset are compared relative to hCoV-19/Wuhan/WIV04/2019 (WIV04), the official reference sequence employed by GISAID (EPI_ISL_402124). Learn more at https://gisaid.org/WIV04.

Supplementary Table 5. Assay limits for (A) the MSD multiplex serology binding antibody assay [V-PLEX SARS-CoV-2 (IgG) Kit k15651u] (conducted by Tomaras lab) and (B) the Nexelis pseudovirus neutralizing antibody assay (conducted by Mkhize lab).

**A. Binding Antibody Assay Limits**				
**SARS-CoV-2 Spike Variant**	**Positivity cut-off (electrochemiluminescence at dilution 500)**	**Positivity cut-off (AU/ml)**	**LLOQ (AU/ml)**	**ULOQ (AU/ml)**
Index (D614, vaccine strain)	5175	156.1	24.5	35000
Alpha (B.1.1.7)	6985	254.0	35	23500
Beta (B.1.351)	3183	123.3	36	10500
Delta (B.1.617.2; AY.4)	2829	93.4	32	20000
Omicron BA.1 (BA.1.1.529)	1141	31.9	14.5	2950
Omicron BA.2	1207	43.6	31	6000
Omicron BA.2.12.1	1488	42.4	16.5	5500
Omicron BA.2.75	1254	28.7	19.5	2850
Omicron BA.5	2031	48.6	17	5500
SARS-CoV-2 Nucleocapsid (N) Index	NA	3970	23	40000
**B. Neutralizing Antibody Assay Limits**				
**SARS-CoV-2 Spike Pseudotyped Virus**			**LOD: ID50**	
Reference (D614G) (vaccine strain with the D614G mutation)			10	
Omicron BA.4/BA.5			10	

*IgG readouts below the antigen-specific positivity cut-off are assigned the value positivity cut-off divided by 2. Neutralization ID50 readouts below the LOD are assigned the value LOD/2. Only the Index (D614) strain MSD assay readouts have been calibrated to the WHO 20/136 International Standard and thus only anti-Spike IgG Index and anti-N IgG Index concentrations can be converted to be expressed in units of binding antibody units (BAU)/ml. For this conversion, the presented value in AU/ml needs to be multiplied by the conversion factor 0.009 for Spike and 0.0024 for N, which convert the readouts to the BAU/ml scale. The Vaccine Research Center established these conversion factors for the MSD assay readout for the D614 Index strain as described in the Supplementary Material of Gilbert et al.[5]

AU, arbitrary units; ID50, 50% inhibitory serum dilution; IU, international units; LOD, limit of detection; LLOQ, lower limit of quantitation; ULOQ, upper limit of quantitation.

Supplementary References

1. Kenny A. Statistical tools for immune correlates analysis of vaccine clinical trial data. PhD dissertation, Department of Biostatistics, University of Washington. 2023.
2. Gilbert PB, Fong Y, Kenny A, Carone M. A Controlled Effects Approach to Assessing Immune Correlates of Protection. *Biostatistics* 2023; **24**(4): 850–65.
3. Magaret CA, Li L, deCamp AC, et al. Quantifying how single dose Ad26.COV2.S vaccine efficacy depends on Spike sequence features. *Nat Commun* 2024; **15**(1): 2175.
4. Greaney AJ, Starr TN, Bloom JD. An antibody-escape estimator for mutations to the SARS-CoV-2 receptor-binding domain. *Virus Evol* 2022; **8**(1): veac021.
5. Gilbert PB, Montefiori DC, McDermott AB, et al. Immune correlates analysis of the mRNA-1273 COVID-19 vaccine efficacy clinical trial. *Science* 2022; **375**(6576): 43-50.

Statistical Analysis Plan for Assessing Binding Antibody and Neutralizing Antibody Immune Correlates in the CoVPN 3008 Study of mRNA-1273 Vaccination in East and Southern Africa: Part A with Follow-up Through 6 Months

Aaron Hudson[1], Bo Zhang[1], Yutong Jin[1], Youyi Fong[1], Jiani Hu[1], Yiwen Lu[1], Chenchen Yu[1], Bhavesh Borate[1], Yunda Huang[1], Peter Gilbert[1]

[1]Vaccine and Infectious Disease Division, Fred Hutchinson Cancer Center, Seattle, Washington

June 26, 2025

Contents

List of Tables 4

List of Figures 5

1 Introduction 6

2 Preliminaries 6

3 Objectives of the Immune Correlates Study 7
3.1 Correlates of Risk (CoR) Objectives 7
3.2 Correlates of Protection (CoP) Objectives 8

4 Immune marker case-cohort sampling measurement plan 9
4.1 Case-cohort sampling 9
4.2 Per-protocol correlates cohorts 9
4.3 Random Immunogenicity Subset of the Per-Protocol Immunogenicity Set 10
4.4 Sampling weights 11
4.4.1 Correlates analyses 11
4.5 Flags in the database 12

5 Immune Markers Assessed in the Correlates Analyses 13
5.1 Antibody markers 13
5.1.1 (1) MSD V-Plex IgG Spike and IgG N binding antibody markers 13
5.1.2 (2) Pseudovirus-nAb assay nAb ID50 15
5.2 Summary of primary immune markers 15
5.3 Definition of immune markers by time point 15
5.4 Positive Response Calls for bAb and nAb Assays 16
5.5 Calculation of categorical/discretized markers into tertiles or dichotomous markers . 16
5.6 Missing data: Imputation of missing immune markers in the per-protocol immunogenicity set 17

6 Study Cohorts 17

7 Study Endpoints for Assessment of Correlates 17

8 Frameworks/Approaches for Assessing Immune Correlates 20
8.1 Correlates of Risk and Correlates of Protection 20

9 Baseline Factors Adjusted for in Immune Correlates Analyses 21
9.1 Baseline Risk Score 22

10 Correlates Analysis Descriptive Tables by Case/Non-Case Status 22

11 Correlates of Risk Analysis Plan 23
11.1 Univariable marker CoR analyses 23

11.2 Multivariable marker CoR analyses 24
11.3 Choice of regression methods 24
11.4 Univariate CoR: Nonparametric threshold regression modeling 26
11.5 P-values and Multiple hypothesis testing adjustment for CoR analysis 27
11.6 Missing data (happenstance) on immune markers 27

12 Correlates of Protection: Interventional Effects 27
12.1 CoP: Controlled Risk and Relative Risk 28
12.1.1 Controlled Risk and Relative Risk Analysis 28
12.1.2 Point and 95% confidence interval estimation of $\mathrm{RR}(1,s)$ and of $RR_C(1;s_1,s_2) = \mathrm{RR}(1,s_2)/\mathrm{RR}(1,s_1)$ assuming the causal assumptions hold 29
12.1.3 Sensitivity analysis (to unmeasured confounding) for the Cox model controlled relative risk analysis 30
12.1.4 Monotone-constrained nonparametric approach 34
12.1.5 Plotting results for controlled risk and controlled relative risk analyses 34
12.2 CoP: Stochastic Interventional Thresholded Effects on Risk and Relative Risk 34

13 Summary of the Set of CoR and CoP Analyses and Their Requirements and Contingencies, and Synthesis of the Results, Including Reconciling Any Possible Contradictions in Results 35

List of Tables

1 Participant flow from the per-protocol efficacy cohort to the per-protocol serum correlates cohort. 10
2 Participant flow from the serum cohort with at least one serum measurement at M0 and Peak to the per-protocol serum immunogenicity set. 10
3 Numbers of Participants in the Per-protocol Serum Immunogenicity Subset 11
4 Inverse probability sampling weights for the serum markers for the correlates analyses. Case and non-case weights are calculated separately. 12
5 Positivity Cutoff Values, Lower and Upper Quantitation Limits of the MSD V-Plex Assay 14
6 Correlates of Risk (CoRs) and Correlates of Protection (CoPs) Objectives for Peak Time Point Markers 21
7 Summary of Stage 1 Peak Marker CoR and CoP Analyses with Requirements/Contingencies for Conduct of the Analysis 36

List of Figures

1 Case-cohort sampling design (Prentice, 1986) that measures M0 and peak immune markers in all participants selected into the Random immunogenicity Subset (subcohort) and in all COVID-19 endpoint cases occurring outside of the subcohort by Month 6. . . . 9
2 Relationship among study endpoints . . . 19

1 Introduction

The multicenter CoVPN 3008 trial assessed the safety of mRNA-1273, the relative effectiveness of hybrid versus vaccine immunity, and SARS-CoV-2 viral persistence among people with HIV (PWH) in East and Southern Africa during the omicron outbreak.

Unvaccinated adults with HIV or another comorbidity associated with severe Covid-19 received one dose of mRNA-1273 (hybrid immunity) or two doses one month apart (vaccine immunity), depending on baseline SARS-CoV-2 serostatus. Part A of CoVPN 3008 assessed the association of hybrid versus vaccine immunity with Covid-19 and severe Covid-19 through to 6 months using covariate-adjusted Cox regression and counterfactual cumulative incidence methods.

Between December 2021 and September 2022, CoVPN 3008 enrolled 14,237 participants; 14,001 (83% PWH, 69% SARS-CoV-2 seropositive) were included in analyses. For PWH, the 6-month cumulative incidence in the hybrid immunity and vaccine immunity groups were 2.02% (95% confidence interval [CI] 1.61-2.44) and 3.40% (95% CI 2.30-4.49) for Covid-19, and 0.048% (95% CI 0.00-0.10) and 0.32% (95% CI 0.59-0.63) for severe Covid-19. The hybrid immunity group had a 42% lower hazard rate of COVID-19 (hazard ratio [HR] 0.58; 95% CI 0.44-0.77; p<0.001) and a 73% lower hazard rate of severe COVID-19 (HR 0.27; 95% CI 0.07-1.04; p=0.056) than the vaccine immunity group. These results can be found in Garrett et al. (2025).

This SAP describes the details of the statistical methods used in the manuscript: *Neutralizing and Binding Antibody Immune Correlates Analysis of the CoVPN 3008 Study of mRNA-1273 Vaccination in People with HIV.*

The manuscript assesses antibody markers measured at baseline and one month post last vaccination (referred to as the peak time point, which is at Peak or M2), as immune correlates of risk and as immune correlates of protection against COVID-19 in Part A of the CoVPN 3008 Study. Because the study has no placebo arm, a correlate of protection refers to a way in which an immune marker measurement causally interrelates to the protective effect of hybrid immunity vs. vaccine immunity (HIPP vs. VIPP) on COVID-19. That is, whereas correlates analyses of phase 3 COVID-19 vaccine efficacy trials assessed correlates of protection based on the vaccine vs. placebo arms, the P3008 Part A correlates analyses assess correlates of protection based on the hybrid immunity vs. vaccine immunity groups. Because these groups are not randomized, it implies the analyses need additional assumptions and sensitivity analyses to understand results and characterize their uncertainty.

2 Preliminaries

All correlates analyses restrict to per-protocol study participants.

Following Garrett et al. (2025), Analysis Group (henceforth AG) AG 1 represents people living with HIV (PWH), overall SARS-CoV-2 status negative, and assigned 2 doses (PWH, vaccine immunity). AG 2-1 represents PWH, overall SARS-CoV-2 status positive (POC anti-S positive), and assigned 1 dose (PWH, hybrid immunity). AG 3 represents HIV-negative, SARS-CoV-2 status negative, and assigned 2 doses (HIV-negative, vaccine immunity). AG 4-1 represents people living without HIV (PWoH), overall SARS-CoV-2 status positive (POC anti-S positive), and assigned 1 dose (PWoH, hybrid immunity).

By definition, participants in the per-protocol hybrid immunity (per-protocol vaccine immunity) analysis group receive one (two) doses of mRNA-1273. We refer to these per-protocol analysis groups as the hybrid immunity per-protocol cohort (AG 2-1 + AG 4-1; henceforth HIPP) and the vaccine immunity per-protocol cohort (AG 1 + AG 3; henceforth VIPP). We also refer to the Overall PP cohort as the union of the HIPP and VIPP cohorts. Only participants qualifying for the HIPP or VIPP cohorts are included in correlates analyses.

The primary marker time point for analysis, referred to as 'peak' for convenience, is the visit 4-weeks post dose 1 (post dose 2) for the HIPP cohort (VIPP cohort).

The correlate of risk (CoR) and correlate of protection (CoP) objectives are only addressed for the CDC COVID-19 endpoint.

In general the correlates analyses consider study endpoints out to 6 months post enrollment. For cumulative-incidence based parameter analyses that use the continuous time-to-event in days, the final time point t_F for data analyses is taken to be the latest time point near the typical number of daya to the month 6 visit at which stable inference can be obtained; for controlled risk analyses $t_F = 165$ days post peak and for thresholded risk analyses $t_F = 160$ days post peak.

For Cox-model based time-to-event analyses and for "final value" analyses that do not use the time-to-event data but instead only register whether the event occurs through to the M6 visit, all events are counted up to the M6 visit date.

The time origin for each time to study endpoint is the peak time point study visit.

All correlates analyses start counting study endpoints 7 days after the peak time point study visit, where participants experiencing the study endpoint earlier than this time point are excluded from the analysis.

All correlates analyses adjust for baseline prognostic factors, chosen to be similar to those adjusted for in the primary Part A manuscript (Garrett et al., 2025) and taking into account the number of endpoints. Variables being adjusted for are listed in Section 9.

Correlates analyses in this manuscript is conducted based on antibody markers only. Given that these markers can be measured from serum samples that were stored from all trial participants, it was possible to obtain these measurements for the majority of COVID-19 endpoint cases.

3 Objectives of the Immune Correlates Study

As noted, the CoR and CoP objectives are only addressed for the CDC COVID-19 endpoint.

3.1 Correlates of Risk (CoR) Objectives

The following CoR objectives are assessed for each individual immune marker that is defined for correlates analysis (so-called 'univariable marker' correlates analyses), for the marker measured at the baseline/M0 time point:

1. To assess the immune markers at M0 as a CoR of COVID-19 by M6, for each of the HIPP and VIPP groups

2. To assess whether PWH status at baseline modifies the CoRs in Objective 1 for each of the HIPP and VIPP groups

The following CoR objectives are assessed for each individual immune marker measured at the peak time point:

1. To assess the immune markers at peak as a CoR of COVID-19 by M6, for each of the HIPP and VIPP groups
2. To assess the fold-rise immune markers from baseline to peak as a CoR of COVID-19 by M6 for the HIPP (not for VIPP) group
3. To assess whether PWH status at baseline modifies the CoRs in Objectives 1 & 2 for each of the HIPP and VIPP groups
4. To assess the immune markers at peak as a CoR of COVID-19 by 3 months post peak, for each of the HIPP and VIPP groups

PWH status is an important participant factor in this study. For serum marker-based CoR analyses, Cox modeling based correlates analyses of each CoR objective will test for a non-zero interaction coefficient of the PWH status intersected with each marker under study, for each of the HIPP and VIPP groups. If the results suggest a general lack of evidence for interactions (which is hypothesized to occur based on the primary Part A relative vaccine efficacy results), then all subsequent CoR and CoP analyses will be done (1) restricting to PWH (HIPP PWH, VIPP PWH) and (2) pooling over PWH and persons living without HIV (HIPP, VIPP), and will not be done for persons living without HIV. If the results support evidence of interaction, then CoR results (but not CoP results) will also be presented for the separate group of persons living without HIV.

The following multivariable marker CoR analyses are conducted for the immune markers:

1. For selected sets of multiple markers, multivariable Cox models to assess the set of markers for their association with COVID-19

3.2 Correlates of Protection (CoP) Objectives

The following CoP objectives are assessed for Part A:

- To assess M0 and peak markers (absolute levels) as controlled risk and controlled relative risk CoPs [hybrid vs. vaccine immunity] against COVID-19 with the controlled effects framework (Gilbert et al., 2023)
- To assess M0 and peak markers (absolute levels) as controlled risk and controlled relative risk CoPs [hybrid vs. vaccine immunity] against COVID-19 with the stochastic threshold-based intervention framework (van der Laan et al., 2023)

4 Immune marker case-cohort sampling measurement plan

4.1 Case-cohort sampling

Figure 1 illustrates the concept of the case-cohort (Prentice, 1986) sampling design that is used for measuring M0 and peak immune markers in a random sample of trial participants. The random sample is stratified by the baseline covariates: HIV-1 serostatus, SARS-CoV-2 point-of-care anti-spike serostatus, and clinical sites with PBMC processing capability. Because the design uses a stratified random sample instead of the simple random sample proposed by Prentice (1986), the design may also be referred to as a "two-phase sampling design" (Breslow et al., 2009b,a), where "phase one" refers to variables measured in all participants and "phase two" refers to variables only measured in a subset (thus the "case-cohort sample" constitutes the phase-two data).

The case-cohort design enables using the same random immunogenicity subset to assess correlates for multiple endpoints, relevant for studying multiple COVID-19 endpoints and potential additional endpoints in future correlates analyses. This makes the design operationally simpler than a case-control sampling design.

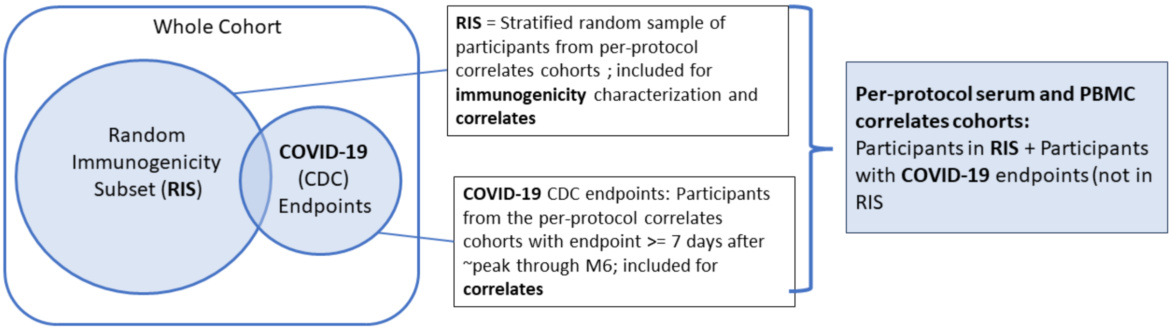

Figure 1: Case-cohort sampling design (Prentice, 1986) that measures M0 and peak immune markers in all participants selected into the Random immunogenicity Subset (subcohort) and in all COVID-19 endpoint cases occurring outside of the subcohort by Month 6.

4.2 Per-protocol correlates cohorts

The raw data include 13,681 Per-Protocol Efficacy set participants, including 13,330 non-cases and 351 cases. Participants who (1) belonged to AG 2-2 or AG 4-2; (2) dropped out of the study by

the peak time point (time origin); (3) had an early CDC endpoint by 7 days post the peak time point; (4) showed evidence of asymptomatic infection (as defined by a $\geq$ 4-times increase in the anti-N titer from M0 to the peak time point); and (5) had an out-of-window peak visit (defined as less than 15 days or exceeding 42 days) are excluded. Table 1 summarizes the number of ptids excluded from each of the above steps. The final per-protocol serum correlates cohort consists of 11,982 participants.

N	Non-cases	Cases	Total
Per-protocol efficacy cohort	13,330	351	13,681
Reasons for exclusion from the per-protocol correlates cohorts:			
– Discordant number of intended/received vaccinations			
(i.e. AG 2-2 or AG 4-2)	1417	42	1459
– Early termination/drop out by peak	54	74	128
– CDC COVID-19 endpoint by 6 days post peak	0	1	1
– $\geq$4-fold increase in anti-N IgG concentration from M0 to peak	15	12	27
– Out-of-window peak visit ($<$ 15 or $>$ 42 days)	367	2	369
Per-protocol serum correlates cohort	11,477	220	11,697

Table 1: Participant flow from the per-protocol efficacy cohort to the per-protocol serum correlates cohort.

4.3 Random Immunogenicity Subset of the Per-Protocol Immunogenicity Set

Random Immunogenicity Subset (RIS) was sampled from all enrolled participants with all of the following information available: baseline HIV-1 status, baseline POC SARS-CoV-2 anti-spike serostatus, availability of serum samples at M0 and peak. For serum sampling, availability of some antibody data at both M0 and peak is required for both IgG and nAb. Table 2 summarizes the number of non-cases and cases from per-protocol efficacy participants with at least one measurement at M0 or peak to the final per-protocol serum immunogenicity set.

N	Non-cases	Cases	Total
Per-protocol RIS and cases	282	351	633
– AG 2-2 or AG 4-2	52	42	94
– Early termination/drop out by peak ($t = 0$)	1	74	75
– CDC endpoint by 6 days post peak (early infection)	0	1	1
– 4-fold increase in anti-N IgG from baseline to peak	15	12	27
– Out-of-window visits ($<$ 15 or $>$ 42 days)	6	2	8
– No IgG measured at M0 or Peak	12	14	26
– No nAb ID50 measured at M0 or Peak	0	0	0
Per-protocol Serum Immunogenicity Set (ph2.m1.sera)	196	206	402

Table 2: Participant flow from the serum cohort with at least one serum measurement at M0 and Peak to the per-protocol serum immunogenicity set.

Table 3 shows the phase-2 sample sizes of the Per-Protocol Serum Immunogenicity Analysis Sets

constituting the immune marker data used for Part A correlates analyses.

Study Group		1	2	3	4	Total
Analysis Group		1	2.1	3	4.1	
HIV		+	+	−	−	
SARS-CoV-2		−	+	−	+	
Serum	Non-cases	37	65	24	70	196
	Cases	49	130	5	22	206
	Total	86	195	29	92	402

Table 3: Numbers of Participants in the Per-protocol Serum Immunogenicity Subset

4.4 Sampling weights

4.4.1 Correlates analyses

Most of the statistical methods use inverse probability of marker sampling weights to account for the case-cohort sampling design that oversamples COVID-19 endpoint cases and uses stratified sampling. In two-phase sampling data analysis nomenclature, the "phase 1 ptids" are all per-protocol serum correlates cohort individuals. The "phase 2 ptids" are then the subset of these phase 1 ptids in the immunogenicity subcohort with M0 and Peak immune marker data available.

Weights are calculated as follows. For baseline sampling stratum k used in the stratified sampling, the IPS weight w_k assigned to a non-case participant in stratum k is defined by $\hat{w}_k = 1/\hat{\pi}(k) = N_k/n_k$, where N_k is the number of per-protocol stratum k non-cases and n_k is the number of these participants that also have M0 and peak marker data available. As the sampling design samples all COVID-19 cases, cases constitute a single stratum with the constant weight defined by the empirical frequency.

Table 4 summarizes the number of per-protocol correlates serum cohort participants in each strata k (N_k), number of participants with marker measurements (n_k), and the corresponding sampling weights ($w_k = N_k/n_k$). Within serum markers, the missingness pattern for various serum markers has been examined and found to be highly concordant; therefore, only one set of weights will be constructed for serum markers.

A total of $K = 5$ strata were defined for the per-protocol serum correlates cohort, including 4 non-cases strata by analysis group (AG 1, AG 2-1, AG 3, and AG 4-1) and 1 cases stratum.

	Non-Cases					Cases
Analysis Group	1	2.1	3	4.1	Total	Cases
HIV	+	+	−	−		
SARS-CoV-2	−	+	−	+		
PP Serum Cohort N_k	2153	7458	329	1537	11,477	220
Serum Measured n_k	37	65	24	70	196	206
Weights N_k/n_k	58.19	114.74	13.71	21.96	-	1.07

Table 4: Inverse probability sampling weights for the serum markers for the correlates analyses. Case and non-case weights are calculated separately.

The sampling of the serum samples corresponded to the Peak time point where the phase-1 sample consisted of N_k or M_k participants and the phase-2 sample consisted of n_k or m_k participants as listed in Table 4. The sampling is done independent of the censoring process for the non-cases and independent of the event times for the cases; therefore, we would expect the weights be applicable for all time points in a cumulative-incidence-based analysis. When the phase-2 sample size is small, as is the case for analyses that only focus on a subgroup (e.g., Analysis Group 2.1), the censoring process or the event time distribution in the phase-2 sample could be quite different from those in the phase-1 sample by chance. In this case, we would use *calibrated weights*. For non-cases, the calibrated weights are defined as the empirical ratio of the number of phase-1 non-cases who remained uncensored by a specified time point t_0 to the number of phase-2 samples who remained uncensored by t_0. For cases, calibrated weights are defined as the ratio of the number of phase-1 cases by t_0 to the number of phase-2 cases by t_0.

4.5 Flags in the database

We generate the following flags for the analysis dataset:

(1) **ph1.m1.sera**: a flag of 11,697 participants (11,477 non-cases and 220 cases). They are free of drop-out, early infections, asymptomatic infections [IgG anti-N ratio (Peak/M0) > 4] and out-of-window second blood draws.

(2) **ph2.m1.sera**: a flag of 402 participants (196 non-cases and 206 cases). In the raw data, these participants have at least one measurement at both M0 and Peak for IgG serum. There is no missing data for neutralization BA.5 titer, whose variable name in the data is“BBA.4/5ID50”

Different analysis cohorts are defined using the flags above.

(Example 1) For correlates analyses of the IgG serum subset that includes both PWH and PWoH (N = 402): ph2.m1.sera==1.

(Example 2) For immunogenicity characterization analysis of serum assays that includes both PWH and PWoH (N = 199): ph2.m1.sera == 1 & IMMFL == “Y”.

5 Immune Markers Assessed in the Correlates Analyses

5.1 Antibody markers

The antibody markers of interest are measured using two different humoral immunogenicity assays:

(1) **IgG bAbs MSD V-Plex**: **IgG binding antibodies** to SARS-CoV-2 proteins (Spike, Nucleocapsid);

(2) **Pseudovirus-nAbs**: **Neutralizing antibodies** against viruses **pseudotyped** with SARS-CoV-2 proteins.

We include the necessary statistical details below.

In the following, the D614 strain (Wuhan-Hu-1 index strain with Spike D614 (NC_045512)) is referred to as Index and the D614G basal outbreak lineage B.1 that bears the D614G mutation compared to the Index strain is referred to as Reference.

Markers as similar as possible to those analyzed in the P3001 and P3005 COVID-19 vaccine efficacy trials will be assessed as immune correlates. The BA.5 Omicron antigen is used as the primary antigen, with parallel analyses conducted for vaccine-strain responses (against Index or Reference) when viewed necessary. In summary, the following types of antibody markers are assessed as correlates, with additional details following: IgG Spike, IgG N, nAb ID50.

IgG anti-N antibodies were not studied as immune correlates in P3001-P3005; however they are included in the assessment of immune correlates for P3008 given that most study participants were previously infected with SARS-CoV-2. In addition, given that IgG N levels may be predictive of the recency of past SARS-CoV-2 infection, this marker may have value as a covariate to adjust for in the analyses of the immune markers measured against Spike protein antigens.

In addition, we may define baseline anti-N low, medium and high tertile, test the interaction between peak and baseline anti-N category, and/or repeat analyses within anti-N-defined baseline subgroups.

5.1.1 (1) MSD V-Plex IgG Spike and IgG N binding antibody markers

Based on the MSD V-Plex panel defined at

`https://www.mesoscale.com/products/`
`v-plex-sars-cov-2-key-variant-spike-panel-1-igg-kit-k15651u/`

IgG concentrations are measured to the following antigens:

- SARS-CoV-2 N (Index)
- SARS-CoV-2 Spike (Index)
- SARS-CoV-2 Spike (B.1.1.7) (Alpha)
- SARS-CoV-2 Spike (B.1.1.529) (BA.1 Omicron)
- SARS-CoV-2 Spike (B.1.351) (Beta)

- SARS-CoV-2 Spike (B.1.617.2; AY.4) (Delta)
- SARS-CoV-2 Spike (BA.2 Omicron)
- SARS-CoV-2 Spike (BA.2.12.1 Omicron)
- SARS-CoV-2 Spike (BA.2.75 Omicron)
- SARS-CoV-2 Spike (BA.5 Omicron)

MSD V-Plex readouts are in Arbitrary Units/ml (AU/ml). For each antigen, positive vs. negative response is computed based on antigen-specific positivity cut-offs provided by the Duke lab, except for the N Index antigen with positive vs. negative response defined by a positivity cut-off defined as follows. To define a positivity cut-off for IgG N Index, we accessed the knowledge that the central lab anti-N qualitative assay used to define M0 seropositive (and hence qualifying for the Hybrid Group) is less sensitive than the MSD IgG N Index assay. The cut-off was set to the minimum value of MSD IgG N Index values at M0 among Hybrid Group participants with central lab anti-N qualitative assay positive result at M0, this cut-off is 3970 AU/ml.

Values below antigen-specific positivity cut-offs are assigned values the positivity cut-off divided by 2.

The positivity cut-offs, lower limits of quantitation (LLOQs), and upper limits of quantitation (ULOQs) of the antigens for the MSD assay are as follows in Table 5. A multiplicative of 500 is applied to the lab-supplied LLOQs and ULOQs to make the same scale as the AU/ml readouts for the study samples and the positivity cutoff values that are based on 1:500 dilution runs.

The LLOQs and ULOQs do not influence the variables.

Table 5: Positivity Cutoff Values, Lower and Upper Quantitation Limits of the MSD V-Plex Assay

Antigens	Spot	Pos. cut-off	LLOQ (AU/mL)	ULOQ (AU/mL)
SARS-CoV-2 Spike	1	5175	24.5	35000
SARS-CoV-2 (BA.2.12.1)	2	1488	16.5	5500
SARS-CoV-2 N	3	3970	23	40000
SARS-CoV-2 Spike (BA.2.75)	4	1254	19.5	2850
SARS-CoV-2 Spike (BA.2)	5	1207	31	6000
SARS-CoV-2 Spike (BA.1.1.529; BA.1)	6	1141	14.5	2950
SARS-CoV-2 Spike (B.1.617 2; AY.4)	7	2829	32	20000
SARS-CoV-2 Spike (B.1.1.7)	8	6985	35	23500
SARS-CoV-2 Spike (B.1.351)	9	3183	36	10500
SARS-CoV-2 Spike (BA.5)	10	2031	17	5500

The MSD D614 Index strain assay is calibrated to the WHO 20/136 International Standard, such that IgG levels to D614 can be translated to units BAU/ml, by multiplying values by 0.009. All analyses are done on the AU/ml scale and reported on the AU/ml scale (i.e., without multiplication by 0.009). If AU/ml units are multiplied by 0.009, then analyses of the D614 Index strain

(Spike) and of the D614 Index strain (N) could be interpreted in terms of Binding Antibody Units (BAU/ml), for comparison with previous correlates analyses (Gilbert et al., 2022; Benkeser et al., 2023).

5.1.2 (2) Pseudovirus-nAb assay nAb ID50

The pseudovirus neutralization assay that is employed, referred to as the VSV nAb assay, is described in the plan "CoVPN 3008 Study plan for the SARS-CoV-2 VSV-Neutralization Antibody Assay." This assay was developed by Nexelis, with LLoQ determined to be 33.

The readouts of the assay is serum inhibitory dilution 50% titer (ID50).

The 2 markers for data analysis are as follows:

- ID50 BA.5 Omicron
- ID50 Reference / D614G

ID50 readouts are in units Arbitrary Units/ml (AU/ml).

For ID50, the LOD is 10 AU/ml, with values below 10 assigned value 5.

ID50 values for the D614G Reference strain can be approximately transformed to international units with the calibration factor 0.242, multiplying AU/ml values by 0.242.

This value was used in the Moderna COVE correlates study (Gilbert et al., 2022) and is approximately accurate based on the Duke/NICD assay concordance study. While correlates results are reported in units AU/ml (without multiplying readouts by the calibration constants), interpretation of results may consider the transformation to WHO international units or to the same, comparable units.

5.2 Summary of primary immune markers

The following markers are initially specified as primary markers.

1. log10 nAb Spike BA.5
2. log10 IgG Spike BA.5
3. log10 IgG Nucleocapsid (N) Index

Note that IgG against N was only measured for the Index strain.

5.3 Definition of immune markers by time point

Each immune marker is studied at M0 and at Peak, except anti-N markers are only studied at M0 given the N protein is not included in the mRNA-1273 vaccine. In addition, the fold-rise markers at Peak are studied, again excluding anti-N markers from the set of fold-rise markers.

5.4 Positive Response Calls for bAb and nAb Assays

For M0 anti-Spike binding antibody responses, positive vs. negative response status is provided by the lab and defined by the raw assay readout in electrochemiluminescent signal being above vs. below the antigen-specific positivity cut-off in the unit of electrochemiluminescent signal. For M0 anti-N binding antibody responses, response status is not provided by the lab because the antigen-specific positivity cutoff is not available. To define a positivity cut-off for anti-N responses, we accessed the knowledge that the central lab anti-N qualitative assay used to define M0 seropositive (and hence qualifying for the Hybrid Group) is less sensitive than the MSD IgG N Index assay. The cut-off was set to the minimum value of MSD IgG N Index values at M0 among Hybrid Group participants with a central lab anti-N qualitative assay positive result at M0, this cut-off is 3970 AU/ml.

For peak anti-Spike and anti-N binding antibody responses, 1) if a participant's M0 response status for a given antigen is negative, then their peak response status for the corresponding antigen is defined in the same way as their M0 response status by using the lab-supplied positivity status for anti-spike and the cutoff of 3970 AU/ml for anti-N responses; 2) if the participant's M0 response status for a given antigen is positive, then their peak response status is positive if Peak AU/ml is at least 4-fold above the M0 AU/ml value, and negative otherwise.

For M0 pseudovirus neutralization antibody responses, positive vs. negative response status is defined by the ID50 value being above vs. below the LOD (=10). For peak neutralization antibody responses, 1) if the participant's M0 response status is negative, then their peak response status is also defined by the peak ID50 value being above vs. below the LOD (=10); 2) if the participant's M0 response status is positive, then their peak response status is positive if peak ID50 is at least 4-fold above the M0 ID50, and negative otherwise.

5.5 Calculation of categorical/discretized markers into tertiles or dichotomous markers

For analysis of the nAb-ID50 markers at each time point, the following rules are used to design a categorical version of the marker:

1. If positive response rate > 0.667, use tertiles
2. If positive response rate is in [0.333, 0.667], still use 3 categories defined as Negative and above and below the median of positive responders
3. If positive response rate < 0.333, switch to 2 categories Positive vs. Negative

For analysis of the IgG Spike markers at each time point, tertiles are used.

Tertiles are calculated separately for the M0 and Peak time point, based on the HIPP serum RIS for antibody markers.

5.6 Missing data: Imputation of missing immune markers in the per-protocol immunogenicity set

At both M0 and Peak, for each assay class, at least one participant in the per-protocol immunogenicity set had marker values measured. Predictive mean matching implemented in the mice package in R is used to impute the missing marker levels. The imputation is conducted separately for each assay type and timepoint (M0 vs. Peak) and stratified by COVID-19 case/non-case status and baseline SARS-CoV-2 status (AG 1 & AG 3 vs AG 2-1 & 4-1). Within each stratum, the following R command is used:

```
mice(log10(dat_in_each_stratum), method = "pmm",m = 1,
               donors = 5L,
               matchtype = 1,
               ridge = 1e-05,
               seed = 1234,
               maxit = 5,
               remove_collinear = FALSE,
               diagnostics = FALSE,
               printFlag = FALSE)
```

with the random seed setting at 1234.

6 Study Cohorts

Antibody immune correlates analyses are conducted in the per-protocol serum correlates cohort (N = 11,697). Details of how to derive these two cohorts can be found in Section 4.2.

Note that these study cohorts exclude AG 2-2 and AG 4-2 defined in the primary paper (Garrett et al., 2025), which are the participants with discordant numbers of primary vaccinations planned vs. received. Thus per-protocol implies receiving the number of intended vaccine doses in the primary series (one or two).

7 Study Endpoints for Assessment of Correlates

The study endpoints of interest for immune correlates assessment are as follows.

- **COVID-19 endpoint:** Defined as virological confirmation of SARS-CoV-2 infection with event date symptom onset (CDC endpoint definition). To be included endpoints must occur at least 7 days after the Peak visit, to help ensure that the endpoint did not occur prior to Peak immune marker measurement.
- **Asymptomatic infection endpoint:** Defined as a viral RNA / NAAT positive test result in the absence of symptoms or an increase in anti-N IgG concentration of at least 4-fold between consecutive visits with anti-N IgG concentration data without a COVID-19 endpoint during the same period of follow-up. The date of acquisition is interval censored between these two visits. For example, for a participant with anti-N IgG value 1000 AU/ml at peak and anti-N IgG value 5000 AU/ml at M6, the participant has an asymptomatic infection endpoint that is

interval censored between peak and M6. If some scheduled visits are missing anti-N IgG data, then the endpoint criterion still applies, although as there are no scheduled visits between peak and M6 this criterion does not apply to Part A. If anti-N serology testing is conducted at unscheduled visits, then an asymptomatic endpoint is still defined by the same 4-fold rise criterion. To be included in correlates analyses, asymptomatic infection endpoints must have the 4-fold increase anti-N IgG event occur at least 7 days after the peak visit. Note that anti-N IgG is not measured in all participants at the key M6 booster visit whereas RNA / NAAT viral testing results are available in generaly for this time point.

- **Infection endpoint:** Defined by the composite of either the COVID-19 endpoint (starting at least 7 days post Peak) or the asymptomatic infection endpoint (starting at least 7 days post Peak), whichever occurs first. Because COVID-19 endpoints are registered on an exact date and asymptomatic infection endpoints are either measured on an exact date (viral positive) interval censored (anti-N serology criterion), the time of infection is partly right-censored and partly interval-censored failure time data.

- **SARS-CoV-2 viral load at COVID-19 onset endpoint:** Defined based on RNA PCR from a sample at COVID-19 onset (e.g., measured by nasal swab).

Figure 2 illustrates the relationship among five study endpoints that are considered in COVID-19 vaccine efficacy trials, where COVID-19 (symptomatic infection, CDC definition) is used as the primary endpoint. This SAP only describes analyses for studying the CDC COVID-19 endpoint.

A

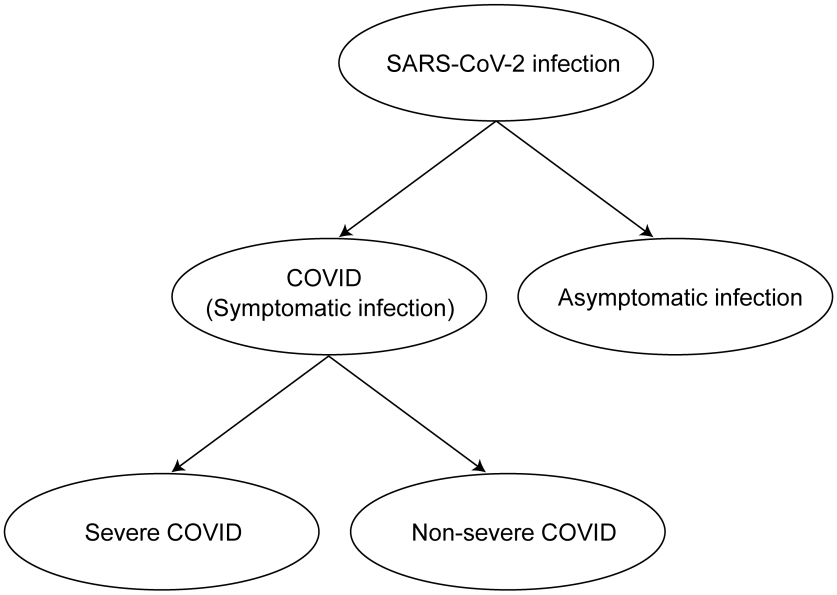

B

Clinical Endpoint	Definition
SARS-CoV-2 infection	Positive RNA PCR test or SARS-CoV-2 seroconversion*, whichever occurs first
COVID (Symptomatic infection)	Meeting a protocol-specified list of COVID-19 symptoms with virological confirmation of SARS-CoV-2 infection (symptom triggered)
Asymptomatic infection	SARS-CoV-2 seroconversion* without prior diagnosis of the COVID endpoint¶
Severe COVID	COVID endpoint with at least one protocol-specified severe disease event
Non-severe COVID	COVID endpoint with zero protocol-specified severe disease events

*Seroconversion is assessed via a validated assay that distinguishes natural vs vaccine-induced SARS-CoV-2 antibodies

¶Alternatively, the asymptomatic infection endpoint can also include an RNA PCR+ test result obtained through testing regardless of symptoms (e.g., as a requirement for travel, return to school or work, or elective medical procedures) and follow-up to confirm the participant remains asymptomatic

Figure 2: Relationship among study endpoints

For immune correlates analyses in this analysis plan, the time of right-censoring for a given failure time endpoint is the first event of loss to follow-up, receipt of outside COVID-19 vaccination, or the date of administrative censoring defined as the last date of available follow-up.

For the Part A follow-up period correlates analyses focusing on M6 endpoints, all available follow-

up for participants is included through to the M6 visit for Cox regression analyses. An earlier time point $t_F = 160$ or $t_F = 165$ days (following that in Garrett et al. (2025)) post Peak visit is used for other analyses (thresholded risk and controlled risk cumulative incidence based analyses). This means that the time of right-censoring for a given failure time endpoint is the M6 visit date in the former case and the specified t_F value in the latter case.

For Peak correlates analyses focusing on endpoints through 3 months post peak, endpoints up to Day 92 are included in Cox regression analyses and $t_F = 92$ is used for other correlates analyses.

8 Frameworks/Approaches for Assessing Immune Correlates

8.1 Correlates of Risk and Correlates of Protection

We broadly classify the proposed analyses into two related categories: correlates of risk (CoR) and correlates of protection (CoP) analyses. CoR analyses seek to characterize correlations/associations of markers with future risk of the outcome amongst vaccinated individuals in a given study cohort such as HIPP or VIPP. CoP analyses seek to formally characterize causal relationships among vaccination, markers and the study endpoint, and use data from both HIPP cohort and VIPP cohort recipients. Gilbert et al. (2024) summarizes CoP objectives and statistical frameworks that are commonly used to these ends, where the versions of the methods that study immune markers in both the HIPP and VIPP cohorts are the relevant methods.

Let RR refer to a COVID-19 endpoint risk ratio (HIPP cohort / VIPP cohort), which could be a cumulative incidence ratio through time t_F. Table 6 lists the objectives and statistical frameworks that are considered to address the objectives for the markers measured at Peak. For the HIPP cohort, the same analyses are relevant for the markers measured at M0.

Table 6: Correlates of Risk (CoRs) and Correlates of Protection (CoPs) Objectives for Peak Time Point Markers

**Objective Type**	**Objective**
**CoRs (Risk Prediction Modeling)**	**To assess peak markers as CoRs in the HIPP or VIPP cohort**<br>a. Relative risks of outcome across marker levels<br>b. Absolute risk of outcome across marker levels<br>c. Statistical/machine learning risk prediction for multivariable markers
**CoP: Correlates of RR**	**To assess peak markers as correlates of RR under assignment to HIPP or to VIPP**<br>a. Principal stratification effect modification analysis<br>b. Assesses RR across subgroups of HIPP participants defined by peak marker level if assigned to the HIPP group<br>Repeat for subgroups of VIPP participants defined by peak marker level if assigned to the VIPP group
**CoP: Controlled Effects on Risk and RR**	**To assess peak markers as controlled risk CoPs**<br>a. To assess peak markers for how assignment to HIPP and to a fixed marker value would alter risk compared to assignment to VIPP.<br>b. Repeat swapping the roles of HIPP and VIPP.
**CoP: Stochastic Interventional Effects on Risk and RR**	**To assess peak markers for as stochastic interventional CoPs**<br>a. To assess peak markers for how stochastic shifts in their distribution would alter mean risk for HIPP compared to the overall risk for VIPP.<br>b. Repeat swapping the roles of HIPP and VIPP.
**CoP: Mediators of RR**	**To assess peak markers as mediators of of the HIPP vs. VIPP RR effect on COVID-19**<br>a. Mechanisms of protection via natural direct and indirect effects<br>b. Estimate the proportion of RR mediated by a marker or markers

This SAP plans for all of the correlates of risk analyses noted above, as well as for conduct of the controlled effects analyses of CoPs. The principal stratification and stochastic interventional analyses are not done in the first set of analyses; they may be added in future correlates analyses.

9 Baseline Factors Adjusted for in Immune Correlates Analyses

Similar to the primary analysis of Garrett et al. (2025), the following baseline variables are adjusted for in the immune correlates analyses of antibody markers: region of enrollment (South Africa versus

any other African country), period of enrollment (< 3 months, 3-6 months, > 6 months post first person enrolled), HIV status, TB status (evidence of prior or active tuberculosis at baseline), and baseline risk score as defined in the next section.

In addition, any analyses pooling over HIPP and VIPP (Overall PP chohort) will adjust for HIPP/VIPP status, because it is strongly predictive of COVID-19 and likely also of most markers at Peak, hence being a likely confounder of the effect of markers on COVID-19.

9.1 Baseline Risk Score

A baseline risk score is developed based on the cohort of unvaccinated South African study participants in the ENSEMBLE trial. This score is built using baseline input variables considered in the efficacy SAP, excluding HIV status at baseline, baseline SARS-CoV-2 status at baseline, baseline CD4 count, or baseline HIV viral load. Specifically our predictors are: age, sex assigned at birth, and BMI, and tuberculosis at baseline. The primary COVID-19 endpoint in the ENSEMBLE trial is used for developing the baseline risk score. The ENSEMBLE data analysis yields a superlearner regression model.

The ENSEMBLE-derived superlearner regression model is applied to all P3008 participants to calculate their baseline risk score values, defined as the logit of the predicted COVID-19 probability, where this logit predicted outcome is scaled to have empirical mean zero and empirical standard deviation one. The classification accuracy of the baseline risk scores for the CDC COVID-19 endpoint in P3008 will be assessed via CV-AUC and CV-ROC curves.

10 Correlates Analysis Descriptive Tables by Case/Non-Case Status

This section considers descriptive analysis of each immune marker defined in Section 5.

For each marker and each of the two "time points" M0 and peak separately, the geometric mean value (GMV) with 95% CI, is reported for each of the case and non-case groups. In addition, for the peak time point the positive responder frequency with 95% CI is reported. In addition, the point and 95% CI estimate of the difference in positive response rate (non-cases vs. cases) and of the GMV ratio (non-cases/cases), is reported. These analyses are done separately for each of the cohorts HIPP, VIPP, HIPP PWH, and VIPP PWH, HIPP PWoH, VIPP PWoH. In addition, corresponding to the Part A correlates results, for antibody markers these analyses are done separately for HIPP and for VIPP (i.e., pooling over the PWH and PWoH groups).

The 95% CIs about the positive responder rates and their differences are computed using the Clopper-Pearson method. The 95% CIs about the GMVs and their ratio are computed using the t-distribution approximation of log-transformed concentrations/titers.

Arrangement of figure display to address the multiple antigen strains that are studied

The following figures are included, which focus on comparing markers between cases and non-cases. The figures are arranged to have panels that show results for each of the six cohorts HIPP, VIPP, HIPP PWH, and VIPP PWH, HIPP PWoH, VIPP PWoH.

Arrangement of figure display to address the multiple antigen strains that are studied

The following figures are included, which focus on comparing markers between cases and non-cases. The figures are arranged to have panels that show results for each of the six cohorts HIPP, VIPP, HIPP PWH, and VIPP PWH, HIPP HIV Negative, VIPP HIV Negative.

Set 1 plots: Immune marker distributions by case/non-case

1. For each immune marker at M0, show side-by-side violin/boxplots for cases vs. non-cases
2. Repeat 1. for peak markers (level)
3. Repeat 1. for peak markers (fold-rise)

Set 2 plots: Line plots for M0 and peak values for cases and non-cases

1. For each immune marker at M0 and peak level, side-by-side violin/box plots for cases vs. non-cases, with lines connecting an individual's data points.
2. For each immune marker at M0 and fold-rise, side-by-side violin/box plots for cases vs. non-cases, with lines connecting an individual's data points.

Set 3 plots: Correlation plots across all markers at a given time point

1. Pairs plot with Spearman rank correlation coefficients for markers at M0
2. Pairs plot with Spearman rank correlation coefficients for markers at peak (absolute level)
3. Pairs plot with Spearman rank correlation coefficients for markers fold-rise from M0 to peak

Sets of correlation plots will be made restricting to specific immunoassays and sets of immune markers.

Set 4 plots: Correlation plots for a given marker across the two time points M0 and peak

1. For each marker, a scatterplot of readouts at M0 and peak absolute level with Spearman rank correlation coefficient.
2. For each marker, a scatterplot of readouts at M0 and fold-rise with Spearman rank correlation coefficient.

11 Correlates of Risk Analysis Plan

As noted, this analysis plan for assessing CoRs and CoPs focuses on the CDC COVID-19 study endpoint, with continuous failure times (failure time defined by the day of the event) and no competing risks. This section details the CoR analyses.

11.1 Univariable marker CoR analyses

The objectives are to assess each immune marker at M0, peak level, and fold-rise, as a CoR for COVID-19 by 6 months. The analyses are done separately for the cohorts VIPP, HIPP, VIPP PWH, and HIPP PWH for the antibody markers.

This objective is addressed by Cox proportional hazards regression, controlled risk analysis by Cox modeling and nonparametric monotone-constrained modeling, and nonparametric threshold regression.

For antibody immune correlates analysis, within the HIPP cohort, Wald 2-sided interaction tests are conducted in the Cox model to assess whether PWH vs. PWoH modifies the CoRs measured at baseline or Peak. The same methods are applied to assess whether PWH vs. PWoH modifies the CoRs measured at Peak within the VIPP cohort.

11.2 Multivariable marker CoR analyses

The multivariable CoR objective is addressed by a multivariable Cox model including multiple marker variables and, depending on the analysis, also interaction terms.

The multivariable Cox model analyses can only support a small number of markers at a time. For immune marker CoR analyses, the following input variable sets are specified for such Cox model fits (which address specific questions). Each of these models use a generalized Wald test for whether the set of markers associate with the COVID-19 endpoint. The antibody marker analyses are done in the HIPP cohort.

1. M0 IgG N Index, M0 IgG Spike BA.4/5
2. M0 IgG N Index, M0 nAb-ID50 BA.4/5
3. M0 IgG Spike BA.4/5, M0 nAb-ID50 BA.4/5
4. M0 IgG Spike BA.4/5, Peak IgG Spike BA.4/5
5. M0 nAb-ID50 BA.4/5, Peak nAb-ID50 BA.4/5

To systematically compare the goodness-of-fit of Cox models with baseline covariates and different immune markers, a partial likelihood ratio test described in Lumley and Scott (2013) and implemented by the function regTermTest in the survey package is used.

11.3 Choice of regression methods

Time-to-event methods of all correlates analyses, both M0 and Peak markers, use the peak marker visit date as the time origin. This is legitimate for analyses of M0 markers because COVID-19 endpoints are not counted until 7 days post peak.

The inverse probability weighted complete-case (IPWCC) Cox regression model designed for case-cohort sampling designs will be used for estimation and inference on hazard ratios of outcomes by M0 and peak marker levels, and for estimation and inference on marginalized marker-conditional cumulative incidence over time. The models will be fit using the *survey*, *CaseCohortCoxSurvival* and *vaccine* R package available on CRAN, and will adjust for the baseline factors.

We use a method from the *survey* package that assumes without replacement two-phase sampling and not Bernoulli sampling, which matches the sampling design and approach to weight estimation (Lumley, 2010). An alternative implementation is provided in the *CaseCohortCoxSurvival* (Etievant and Gail, 2024).

The final time point t_F of follow-up for cumulative incidence parameter based correlates analyses is taken to be 165 days for controlled risk antibody marker analyses and 160 days for thresholded risk antibody marker.

For Cox model based analyses of hazard ratio parameters that does not assess cumulative incidence type parameters, all follow-up is used through to the M6 visit, amounting to a maximum of 230 days follow-up post Peak.

Let T be the failure time, S a M0 or peak marker of interest, and X the vector of baseline factors that are adjusted for. With $S_1(t|s,x) = P(T > t|S = s, X = x, A = 1)$, the Cox model fit yields an estimate of $S_1(t|s, X_i)$ for each individual i in the phase-two sample. The marginalized conditional risk $risk_1(t|s) = E_X[P(T \le t|s, X, A = 1)]$ through time t (for all times t through t_F simultaneously) is estimated based on the equation

$$risk_1(t|s) \quad = \quad \int (1 - S_1(t|s,x))dH(x) \tag{1}$$

where $H(\cdot)$ is the distribution of X in $A = 1$ individuals.

The function $risk_1(t|s)$ can be estimated by

$$\widehat{risk}_1(t|s) = \frac{\sum_{i=1}^{n} \frac{1}{\hat{\pi}(X_i)}(1 - \hat{S}_1(t|s, X_i))}{\sum_{i=1}^{n} \frac{1}{\hat{\pi}(X_i)}}, \tag{2}$$

where n is the number of participants with phase-two data.

When the *survey* R package is used, the bootstrap is used to obtain 95% pointwise confidence intervals for $risk_1(t_F|s)$ and 2-sided p-values. When the *vaccine* R package is used, analytic influence-curve based 95% pointwise confidence intervals and 2-sided p-values.

The bootstrap process will be performed by resampling with replacement the subjects within the subcohort and the subjects outside the subcohort separately within each stratum and by resampling with replacement subjects with undetermined stratification variables. Across all bootstrap samples, the number of participants in each stratum in the Random immunogenicity subset remains fixed, but the number of cases does not stay the same.

The results of the above Cox modeling will be output in a variety of ways, conducted separately for the M0 and peak level time points. For antibody markers the analyses are done for each of the four cohorts HIPP, VIPP, HIPP PWH, VIPP PWH. The details below apply for the analysis of the HIPP cohort (with VIPP as the comparator cohort). The same analyses are conducted swapping the roles of the HIPP and VIPP cohorts. If in either the HIPP or VIPP cohorts a marker does not evidence as a CoR, then the CoP analysis using the other cohort as the comparator cohort may be canceled.

1. Plot $\widehat{risk}_1(t_F|s)$ vs. s with 95% CIs for continuous $S = s$ varying over its whole range. Include on the plot the estimate of $\widehat{risk}_0(t_F)$ with a 95% CI for the VIPP arm (horizontal bands), computed by a Cox model marginalizing over the same baseline factors as for the analysis of the HIPP cohort. A version of this plot shows the two curves $\widehat{risk}_0(t_F)$ for VIPP and HIPP on the same plot.

2. Based on a fit of the Cox model to a nominal categorical marker defined as the tertiles of S, plot $\widehat{risk}_1(t|s)$ for each category of S values with 95% CIs, for all time points t from the time origin through t_F. If more than 20% of vaccine recipients have S below the positivity cut-off or LOD of the assay, then the categories instead will be (1) values $\leq$ positivity cut-off/LOD; (2) values below the median of values $>$ positivity cut-off/LOD; (3) values above the median of values $>$ LOD. Include on the plot the estimated curve $\widehat{risk}_0(t)$ with 95% CIs for the VIPP cohort, computed by a Cox model marginalizing over the same baseline factors as for the analysis of the vaccine arm.

3. Tabular reporting of the hazard ratio per 10-fold change in the quantitative M0 or peak marker with 95% confidence interval and 2-sided p-value.

4. The multivariable Cox models are implemented in two ways: reporting results per 10-fold change in markers and per SD-fold change in markers (the latter results are used for enabling a comparison of the magnitudes of association across the multiple immune markers).

5. Tabular reporting of the hazard ratio for the Middle and Upper categories of the categorical marker vs. the Lower category, with 95% confidence interval and 2-sided p-value, as well as a global generalized Wald two-sided p-value for whether the hazard rate of the endpoint varies across the three categories. The table includes the attack rate (with no. of cases / no. at risk) through t_F for each of the three marker-defined subgroups and for the VIPP arm.

6. Report point and 95% CI estimates for the hazard ratio per 10-fold change in the marker. A forest plot is used to show results for the 6 cohorts that are analyzed.

The bootstrap or analytic approach of the *vaccine* R package is used to calculate 95% pointwise CIs for $risk_1(t_F|s)$ in s. The 2-sided Wald p-value for testing the regression coefficient of the marker in the Cox model provides a valid test of the null hypothesis $H_0 : risk_1(t_F|s) = risk_1(t_F)$ for all s, and is reported.

11.4 Univariate CoR: Nonparametric threshold regression modeling

The van der Laan et al. (2023) extension of the nonparametric CoR threshold estimation method of Donovan et al. (2019) is applied to each of the immune markers at M0 and peak level using the version of the method that accounts for right-censoring of the binary outcome of interest $Y = I(T \leq t_F)$ where $Y = 1$ is the COVID-19 endpoint occurring by time t_F. The analyses adjust for the same baseline factors X as used in the Cox model CoR analyses.

The extension adjusts for baseline covariates by estimating the conditional mean function $E[Y|S \geq s, X, A = 1]$ using discrete-SuperLearner and then empirically averaging over the baseline covariates X to estimate the marginal risk $risk_1^Y(S \geq s) = E_X[P(Y = 1|S \geq s, X, A = 1)]$ for each threshold s of the the marker in a specified discrete set. We do not perform pooled regression across the thresholds s, which ensures we are totally nonparametric in estimating the threshold dependence of $risk_1^Y(S \geq s)$ on s. The SuperLearner library includes a range of increasingly flexible parametric learners including logistic regression (glm), bayesian logistic regression (bayesglm), and L1-penalized logistic regression (glmnet). (Two of each learner is included in the library, one with only main-term variables and another with main-term and interaction variables.) An advantage of the nonparametric CoR threshold method compared to Cox modeling that specifies a log

linear hazard ratio with the marker is that it can potentially detect a threshold of very low risk. The method is implemented with and without the monotonicity constraint that $risk_1^Y(S \geq s)$ is monotone non-increasing in s, where the results assuming monotonicity are reported unless there is evidence for violation of this assumption.

The results are reported in the same way that Donovan et al. (2019) reports results in its Figure 2, where point estimates, pointwise 95% confidence bands, and simultaneous 95% confidence bands for $risk_1^Y(S \geq s)$ are plotted for a range of threshold values. The simultaneous confidence bands cover the entire curve in s with at least 95% probability and are useful for judging whether risk varies over threshold subgroups, whereas the pointwise 95% confidence bands are useful for quantifying precision at particular threshold values. The method uses the same empirical two-phase sampling estimated weights (IPS weights) as used for the other univariable IPWCC CoR analyses.

The analysis is done using targeted maximum likelihood estimation (TMLE) as described in van der Laan et al. (2023), and the pointwise and simultaneous simultaneous confidence bands are of the Wald-type, obtained from the asymptotic distribution of the TMLE.

11.5 P-values and Multiple hypothesis testing adjustment for CoR analysis

In general, p-values are only reported from pre-specified and automated (press-button) analyses. For the CoR analyses, p-values are reported for the Cox regression analyses of each of the specified marker variables. For each time point M0 and Peak, two-sided p-values for hypothesis testing of each marker CoR are calculated for the Cox regression of quantitative markers (two-sided Wald tests). For Cox models with 2 or 3 markers, a two-sided generalized Wald test is included. Holm-Bonferroni family-wise error rate (FWER)-adjusted p-values are computed along with nominal unadjusted p-values. FWER p-values ≤ 0.05 are flagged as having statistical evidence for being a CoR.

11.6 Missing data (happenstance) on immune markers

Section 5.6 describes how the missing marker data was handled based on predictive mean matching single imputation. Correlates analyses are conducted using the same singly imputed dataset.

12 Correlates of Protection: Interventional Effects

In these analyses, we seek to understand whether, how, and to what extent peak markers impact risk and relative risk in causal ways. We describe two approaches to this problem. Each involves consideration of a counterfactual time-to-event outcome $Y(a, s)$ under a hypothetical intervention that both sets randomization assignment $A = a$ and sets the immune marker at Peak, S, to a fixed value or based upon a random draw from a analyst-specified distribution. Below, we assume that S is scalar-valued, but some of the approaches below naturally extend to the case where a vector of immunologic markers are considered (currently such analyses are not planned). Given the central goal to define parsimonious surrogate endpoints based on a single immunoassay, the main analysis will use each of the methods to assess each of the quantitative markers separately as CoPs, adjusting for the same set of baseline covariates as used in the CoR analyses previously described in Section 11.

12.1 CoP: Controlled Risk and Relative Risk

Controlled risk and relative risk analyses are done for the M0, peak level, and fold-rise immune markers.

12.1.1 Controlled Risk and Relative Risk Analysis

The analysis is done for the peak level and fold-rise markers. The methods use the time-to-event outcome approach accounting for right-censoring as described in Gilbert et al. (2023).

We consider the causal parameters the controlled risk curve and the controlled relative risk curve, defined as

$$\begin{aligned} r_C(1,s) &= P(Y(1,s)=1) \\ \mathrm{RR}(1,s) &= \frac{P(Y(1,s)=1)}{P(Y(0)=1)} \end{aligned}$$

where these parameters focus on assignment to the HIPP group. Analogous parameters are defined for the VIPP group:

$$\begin{aligned} r_C(0,s) &= P(Y(0,s)=1) \\ \mathrm{RR}(0,s) &= \frac{P(Y(0,s)=1)}{P(Y(1)=1)}. \end{aligned}$$

We describe the methodology for $r_C(1,s)$ and $\mathrm{RR}(1,s)$, noting that the same methodology applies for $r_C(0,s)$ and $\mathrm{RR}(0,s)$.

The value of $\mathrm{RR}(1,s)$ represents the relative decrease in endpoint frequency achieved by administering HIPP and setting peak immunologic marker level to s compared to the VIPP control intervention with no intervention on the immune marker. Under our approach, the value of $r_C(a,s)$ is assumed to be monotone non-decreasing in s for each a; in other words, controlled risk can only potentially become smaller by setting greater marker levels. The extent to which the marker plays a role in determining risk and relative risk can be determined by the degree of flatness of the graph of $r_C(a,s)$ and $\mathrm{RR}(a,s)$ versus s.

Now, $P(Y(0)=1) = E_X[P(Y=1 \mid A=0,X)]$ whenever $Y(0)$ and A are independent given a vector X of covariates, and $P(A=1 \mid X) > 0$ almost surely. Under this assumption that the study is as if HIPP vs. VIPP was randomized, the controlled relative risk $\mathrm{RR}(1,s)$ at level s can be identified using the fact that

$$P(Y(1,s)=1) = E\left[P(Y=1 \mid S=s, A=1, X)\right]$$

whenever $Y(1,s)$ and S are independent given $A=1$ and a vector X of covariates, and $P(S=s \mid A=1,X) > 0$ almost surely. In other words, identification of the controlled relative risk $\mathrm{RR}(1,s)$ requires that a rich enough set of covariates be available so that deconfounding of the relationship between endpoint Y and marker S is possible in the HIPP subpopulation (no-unmeasured confounding assumption), and that marker level $S=s$ may occur within each subpopulation defined by values of the covariates X (positivity assumption).

The parameter $\mathrm{RR}(1, s)$ is estimated for values of s ranging between the median of S for the $A = 0$ group up to the 95th percentile of S for the $A = 1$ group. This is done because setting S to the median value for the $A = 0$ group implies $\mathrm{RR}(1, s)$ is approximately similar to a controlled direct effect, a useful anchor point of the analysis, where a good surrogate endpoint will have a controlled direct effect near 1.0. Similarly, the parameter $\mathrm{RR}(0, s)$ is estimated for values of s ranging between the median of S for the $A = 1$ group up to the 95th percentile of S for the $A = 0$ group. The median and 95th percentile are computed using IPS weighting.

12.1.2 Point and 95% confidence interval estimation of $\mathbf{RR}(1, s)$ and of $RR_C(1; s_1, s_2) = \mathbf{RR}(1, s_2)/\mathbf{RR}(1, s_1)$ assuming the causal assumptions hold

In this subsection, we describe how the point and 95% confidence interval estimates for $\mathrm{RR}(1, s)$ that are reported in the main article and the Supplement are calculated, which assume that causal assumptions mentioned above hold. In this subsection we also describe how the point and 95% confidence interval estimates for $RR_C(1; 0, 1) = \mathrm{RR}(1; 1, 1)/\mathrm{RR}(1; 1, 0))$ for a binary marker S are calculated, with results for $S = 1$ representing the upper tertile and $S = 0$ representing the lower tertile. In the next subsection, we describe how the sensitivity analysis is conducted, which quantifies the sensitivity of the results to potential unmeasured confounding.

(Gilbert et al., 2023) details the inferential and sensitivity analysis approach. We summarize here the key details needed for understanding the analysis of P3008 Part A. Under the two causal assumptions, the numerator term $P(Y(1, s) = 1)$ of RR(1,s) $= P(Y(1, s) = 1)/P(Y(0) = 1)$ is

$$P(Y(1, s) = 1) = E\left[P(Y = 1 \mid S = s, A = 1, X)\right] = risk_1(t_F|s),$$

as defined in Section 11.3, using the notation of Section 11.3. That section described the Cox modeling approach that was used to compute an estimate $\widehat{risk}_1(t_F|x)$ of $risk_1(t_F|s)$, where $Y = I(T \leq t_F)$, T is the time from the peak marker measurement date until the COVID-19 outcome starting 7 days post peak.

The same estimate $\widehat{risk}_1(t_F|s)$ is used to estimate the numerator term $P(Y(1, s) = 1)$ of RR(1,s). That is, there is a harmonization of the correlate of risk and controlled RR analyses, where the estimate $\widehat{risk}_1(t_F|x)$ used for the former is also used for the numerator term $P(Y(1, s) = 1)$ of RR(1,s) for the latter:

$$\widehat{\mathrm{RR}}(1, s) = \frac{\widehat{risk}_1(t_F|x)}{\widehat{P}(Y(0) = 1)}$$

(where we detail the estimator $\widehat{P}(Y(0) = 1)$ next). For instance, for analysis of the ID50 titer marker in the main article, the estimate $\widehat{risk}_1(t_F|x)$ is also used for the estimate of RR(1,s).

To estimate the denominator of RR(1,s), $P(Y(0) = 1) = P(Y = 1 \mid A = 0) = P(T \leq t_F|A = 0)$, we use an estimation approach compatible with the approach used to estimate the numerator $\widehat{risk}_1(t_F|s)$ – in this case a Cox model. Accordingly, $E[P(Y = 1 \mid A = 0, X)]$ is estimated with a standard Cox model, with point estimate the average of the fitted values $\widehat{E}[P(Y_i = 1|A_i = 0, X_i)]$ across the VIPP participants. Then, the point estimate of RR(1,s) is computed as one minus the ratio of the numerator point estimate divided by the denominator point estimate. Pointwise

95% confidence intervals for $RR(1,s)$ were computed using the same set of bootstrap estimates of the numerator $\widehat{risk}_1(t_F|s)$ as used for the correlates of risk analysis, and also including bootstrap estimates of the denominator $\widehat{E}[P(Y=1\,|\,A=0,X)]$. The nonparametric percentile bootstrap method is used for the confidence intervals.

12.1.3 Sensitivity analysis (to unmeasured confounding) for the Cox model controlled relative risk analysis

Sensitivity analysis is generally warranted when a no-unmeasured confounders assumption is made. The sensitivity analysis quantifies the rigor of evidence for a controlled RR CoP after accounting for potential bias from unmeasured confounding. We define S to be a controlled RR CoP if $\mathrm{RR}(1,s)$ is monotone non-decreasing in s with RR(1,s) $<$ RR(1,s') for at least some $s < s'$, where point and 95% confidence interval estimates of $\mathrm{RR}(1,s)$ versus s, with built in robustness to unmeasured confounding, describe the strength of the CoP in terms of the amount and nature of increase. Because the denominator $P(Y(0)=1)$ of $\mathrm{RR}(1,s)$ does not depend on s, a controlled RR CoP can equivalently be defined as the numerator $P(Y(1,s)=1)$ being monotone non-increasing in s with $P(Y(1,s)=1) > P(Y(1,s')=1)$ for at least some $s < s'$, where point and 95% confidence interval estimates of $P(Y(1,s)=1)$ versus s indicate some robustness to unmeasured confounding.

Two sensitivity analyses are conducted, the first of which considers the binary immunologic marker S with 0 indicating the first tertile and 1 indicating the third tertile. The second sensitivity analysis considers the quantitative marker S varying over its full range.

As set-up for both sensitivity analyses, for any two marker values s_1 and s_2, define the controlled risk ratio

$$RR_C(1;s_1,s_2) = \frac{r_C(1,s_2)}{r_C(1,s_1)} = \frac{\mathrm{RR}(1,s_2)}{\mathrm{RR}(1,s_1)},$$

where $r_C(1,s) = P(Y(1,s)=1)$ is the controlled risk at $S=s$. From the observed data without the causal assumptions, the statistical parameters $r_M(1,s) = risk_1(t_F|s)$ (the marginalized conditional risk) and

$$RR_M(1;s_1,s_2) = \frac{r_M(1,s_2)}{r_M(1,s_1)}$$

(the marginalized conditional risk ratio) can be estimated. Moreover, under the causal assumptions (no-unmeasured confounding and positivity), $r_M(1,s) = r_C(1,s)$ and $RR_M(1;s_1,s_2) = RR_C(1;s_1,s_2)$. Given that CoR analysis is based on observational data — the biomarker value is not randomly assigned — a central concern is that unmeasured or uncontrolled confounding of the association between S and Y could render $r_M(1,s) \neq r_C(1,s)$, biasing estimates of the causal parameters of interest $r_C(1,s)$ and $RR_C(1;s_1,s_2)$. Because we can never be certain that confounding is adequately adjusted for, sensitivity analysis is warranted, as considered in extensive literature — see, e.g., VanderWeele and Ding (2017) and references therein.

Sensitivity analysis is useful to evaluate how strong unmeasured confounding would have to be to explain away an observed causal association, that is, to determine the strength of association of an unmeasured confounder between S and Y needed for the observed exposure-outcome association

to not be causal, $r_M(1,s) \neq r_C(1,s)$ and $RR_M(1;s_1,s_2) \neq RR_C(1;s_1,s_2)$. We follow the recommendation of VanderWeele and Ding (2017) to report the E-value as a summary measure of the evidence of causality, or, in our application, evidence of whether S is a controlled risk CoP based on variation in the controlled risk curve. We also include other closely related measures of sensitivity.

The E-value is the minimum strength of association, on the risk ratio scale, that an unmeasured confounder would need to have with both the exposure variable (S) and the outcome (Y) in order to fully explain away a specific observed exposure–outcome association, conditional on the measured covariates [VanderWeele and Ding (2017); VanderWeele and Mathur (2020)]. Here, in this section alone, we refer to the marker S as an "exposure" variable following the typical set-up in the causal inference statistical methods literature. If, as in CoP analyses, the estimated marginalized risk ratio $\widehat{RR}_M(1;s_1,s_2) = \widehat{r}_M(1,s_2)/\widehat{r}_M(1,s_1)$ for $s_1 < s_2$ is less than one, then the E-value for $\widehat{RR}_M(1;s_1,s_2)$ is calculated as

$$e_{RR}(1;s_1,s_2) = \frac{1+\sqrt{1-\widehat{RR}_M(1;s_1,s_2)}}{\widehat{RR}_M(1;s_1,s_2)}\,. \tag{3}$$

We include the argument (s_1,s_2) in the notation, with $s_1 < s_2$ by convention, to be clear that the E-value depends on specification of two specific marker-level subgroups.

To illustrate the interpretation of an E-value, suppose S is binary with levels 0 and 1 and regression analysis yields an estimate $\widehat{RR}_M(1;0,1) = \widehat{r}_M(1,1)/\widehat{r}_M(1,0) = 0.40$ with 95% confidence interval (CI) $(0.14, 0.78)$. An E-value $e(1;0,1)$ of 4.4 means that a marginalized risk ratio $RR_M(1;0,1)$ at the observed value 0.40 could be explained away (i.e., $RR_C(1;0,1) = 1.0$) by an unmeasured confounder associated with both the exposure and the outcome by a marginalized risk ratio of 4.4-fold each, after accounting for the vector X of measured confounders, but that weaker confounding could not do so.

In addition, we follow the recommendation of VanderWeele and Ding (2017) to also report the E-value $e_{UL}(1;s_1,s_2)$ for the upper limit $\widehat{UL}(1;s_1,s_2)$ of the 95% CI for the observed marginalized risk ratio $\widehat{RR}_M(1,s_1,s_2)$, computed as 1 if $\widehat{UL}(1;s_1,s_2) \geq 1$ and, otherwise, as

$$\frac{1+\sqrt{1-\widehat{UL}(1;s_1,s_2)}}{\widehat{UL}(1;s_1,s_2)}\,,$$

which in the example equals $e_{UL}(1;0,1) = 1.88$. This E-value for the upper limit indicates, for given $s_1 < s_2$, the strength of unmeasured confounding at which statistical significance of the inference that $RR_C(1;s_1,s_2) < 1$ would be lost. The two E-values above are useful for judging how confident we can be that an immunologic biomarker is a controlled risk CoP, with E-values near one suggesting weak support and evidence increasing with greater E-values.

Because $RR_C(1;s_1,s_2) = RR(1,s_2)/RR(1,s_1)$, evidence for $RR_C(1;s_1,s_2) < 1$ is equivalently evidence for $RR(1,s_1) < RR(1,s_2)$. Thus $RR_C(1;s_1,s_2)$ can be interpreted as the multiplicative degree of superior relative risk caused by marker level s_2 vs. marker level s_1, and E-values quantify evidence for whether $RR(1,s_1)$ is less than $RR(1,s_2)$. It is also useful to provide conservative estimates of controlled risk ratios and of the controlled risk curve, accounting for unmeasured

confounding. We approach these tasks based on the sensitivity analysis, or bias analysis, approach of Ding and VanderWeele (2016). We give their main result and refer readers to the paper for details.

We begin by defining two (possibly context-specific) fixed sensitivity parameters. First, we set $RR_{UD}(1; s_1, s_2)$ to be the maximum risk ratio for the outcome Y comparing any two categories of the unmeasured confounders U, within either exposure group $S = s_1$ or $S = s_2$, conditional on the vector X of observed covariates. Second, we set $RR_{EU}(1; s_1, s_2)$ to be the maximum risk ratio for any specific level of the unmeasured confounder U comparing individuals with $S = s_1$ to those with $S = s_2$, with adjustment already made for the measured covariate vector X. Thus, $RR_{UD}(1; s_1, s_2)$ quantifies the importance of the unmeasured confounder U for the outcome, and $RR_{EU}(1; s_1, s_2)$ quantifies how imbalanced the exposure/marker subgroups $S = s_1$ and $S = s_2$ are in the unmeasured confounder U. The values $RR_{UD}(1; s_1, s_2)$ and $RR_{EU}(1; s_1, s_2)$ are always specified as greater than or equal to one. We suppose that $RR_M(1; s_1, s_2) < 1$ for the fixed values $s_1 < s_2$ — this is the case of interest for immune correlates.

Define the bias factor

$$B(1; s_1, s_2) = \frac{RR_{UD}(1; s_1, s_2) RR_{EU}(1; s_1, s_2)}{RR_{UD}(1; s_1, s_2) + RR_{EU}(1; s_1, s_2) - 1}$$

for $s_1 \leq s_2$, and define $RR_M^U(1; s_1, s_2)$ the same way as $RR_M(1; s_1, s_2)$, except marginalizing over the joint distribution of X and U. Then, $RR_M^U(1; s_1, s_2) \leq RR_M(1; s_1, s_2) \times B(1; s_1, s_2)$, where $RR_M^U(1; s_1, s_2) = E\{r(1; s_2, X^*)\}/E\{r(1; s_1, X^*)\}$ with $X^* = (X, U)$ and $r(1; s, x, u) = P(Y = 1 \,|\, S = s, A = 1, X = x, U = u)$ conditional risk. Translating this result to our problem context, under the positivity assumption, we have that $RR_M^U(1; s_1, s_2) = RR_C(1; s_1, s_2)$ and so, it follows that

$$RR_C(1; s_1, s_2) \leq RR_M(1; s_1, s_2) \times B(1; s_1, s_2) \ . \tag{4}$$

This inequality states that the controlled risk ratio is bounded above by the marginalized risk ratio multiplied by the bias factor. It follows that a conservative (upper bound) estimate of $RR_C(1; s_1, s_2)$ is obtained as $\widehat{RR}_M(1; s_1, s_2) \times B(1; s_1, s_2)$, and a conservative 95% CI is obtained by multiplying each confidence limit for $RR_M(1; s_1, s_2)$ by $B(1; s_1, s_2)$. These estimates for $RR_C(1; s_1, s_2)$ account for the presumed-maximum plausible amount of deviation from the no unmeasured confounders assumption specified by $RR_{UD}(1; s_1, s_2)$ and
$RR_{EU}(1; s_1, s_2)$. An appealing feature of this approach is that the bound (4) holds without making any assumption about the confounder vector X or the unmeasured confounder U.

Conservative (bounded) estimation of $r_C(1, s)$ and $RR_C(1; s_1, s_2)$ for a quantitative marker S

The above approach does not directly provide a conservative estimate of the controlled risk curve $r_C(1, s)$, because additional information is needed for absolute versus relative risk estimation. To provide conservative inference for $r_C(1, s)$, we next select a central value s^{cent} of S such that $\widehat{r}_M(1, s^{cent})$ matches the observed overall risk, $\widehat{P}(Y = 1|A = 1)$. This value is a 'central' marker value at which the observed marginalized risk equals the observed overall risk. Next, we 'anchor' the analysis by assuming $r_C(1, s^{cent}) = r_M(1, s^{cent})$, where picking the central value s^{cent} makes

this plausible to be at least approximately true. Under this assumption, the bound (4) implies the bounds

$$r_C(1,s) \leq r_M(1,s)B(1;s^{cent},s) \quad \text{if} \quad s \geq s^{cent} \tag{5}$$

$$r_C(1,s) \geq r_M(1,s)\frac{1}{B(1;s,s^{cent})} \quad \text{if} \quad s < s^{cent}. \tag{6}$$

Therefore, after specifying $B(1;s^{cent},s)$ and $B(1;s^{cent},s)$ for all s, we conservatively estimate $r_c(1,s)$ by plugging $\widehat{r}_M(1,s)$ into the formulas (5) and (6).

Because $B(1;s_1,s_2)$ is always greater than one for $s_1 < s_2$, formula (5) pulls the observed risk $\widehat{r}_M(1,s)$ upwards for subgroups with high biomarker values, and formula (6) pulls the observed risk $\widehat{r}_M(1,s)$ downwards for subgroups with low biomarker values. This makes the estimate of the controlled risk curve flatter, closer to the null curve, as desired for a sensitivity/robustness analysis.

To specify $B(1;s_1,s_2)$, we note that it should have greater magnitude for a greater distance of s_1 from s_2, as determined by specifying $RR_{UD}(1;s_1,s_2)$ and $RR_{EU}(1;s_1,s_2)$ increasing with $s_2 - s_1$ (for $s_1 \leq s_2$). We consider one specific approach, which sets $RR_{UD}(1;s_1,s_2) = RR_{EU}(1;s_1,s_2)$ to the common value $RR_U(1;s_1,s_2)$ that is specified log-linearly: $\log RR_U(1;s_1,s_2) = \gamma(s_2 - s_1)$ for $s_1 \leq s_2$. Then, for a user-selected pair of values $s_1 = s_1^{fix}$ and $s_2 = s_2^{fix}$ with $s_1^{fix} < s_2^{fix}$, we set a sensitivity parameter $RR_U(1;s_1^{fix},s_2^{fix})$ to some value above one. It follows that

$$\log RR_U(1;s_1,s_2) = \left(\frac{s_2 - s_1}{s_2^{fix} - s_1^{fix}}\right)\log RR_U(1;s_1^{fix},s_2^{fix}), \qquad s_1 \leq s_2.$$

We anchor the analysis by setting $s_1 = s_1^{fix}$ at the 15th percentile of the peak marker and $s_2 = s_2^{fix}$ at the 85th percentile of the peak marker.

Once $r_C(1,s)$ is conservatively estimated via the formulas (5) and (6), it is immediate how to obtain a conservative estimate of $\mathrm{RR}(s)$:

$$\widehat{\mathrm{RR}}(1,s) = 1 - \frac{\widehat{r}_M(1,s)B(1;s^{cent},s)}{\widehat{P}(Y(0)=1)},$$

where the estimate of the VIPP arm risk, $\widehat{P}(Y(0)=1)$, is the same as for the controlled RR analysis assuming no-unmeasured confounders.

Sensitivity analyses for controlled relative risk

The sensitivity analysis is done for each of the two Cox model CoR analyses described in Section 11.3, first for the binary peak marker and second for the quantitative peak marker. For the former analysis, E-values are reported for both the point estimate and the upper 95% confidence limit for $RR_C(1;0,1)$, where category 1 is the upper tertile (vaccine recipients with antibodies S in the top third), category 0 is the lower tertile (vaccine recipients with antibodies in the bottom third), and the intermediate middle tertile subgroup of vaccine recipients is excluded from the analysis. In addition, we set $RR_{UD}(1;0,1) = RR_{EU}(1;0,1) = 2$, such that $B(1;0,1) = 4/3$, and report conservative estimation and inference on the controlled risk ratio $RR_C(1;0,1)$ and equivalently on the ratio of controlled relative risk curves $RR_C(1;0,1) = RR(1,1)/RR(1,0)$.

Next, we conduct the sensitivity analysis treating S as a quantitative variable, as detailed in Section "Conservative (bounded) estimation of $r_C(1; s)$ and $RR_C(1; s_1, s_2)$ for a quantitative marker S." This analysis reports results in terms of point and 95% point-wise confidence interval estimates of $\mathrm{RR}(1, s)$ vs. s assuming the specified amount of unmeasured confounding that makes the estimates of $\mathrm{RR}(1, s)$ flatter than under the assumption of no unmeasured confounding.

For validity the controlled risk/relative risk analyses require the positivity assumption, and thus the methods will only be applied if the data are reasonably supportive of the positivity assumption. To check positivity, we study the marker distribution in vaccine recipients within each subgroup of the covariates X that are adjusted for. For the tertiles analysis we require evidence that within each subgroup some vaccine recipients have lower tertile responses and some vaccine recipients have upper tertile responses. For the quantitative S analysis, we look for evidence that S varies over its full range within each level of the potential confounders that are adjusted for.

Estimation of the controlled risk and controlled relative risk parameters are done for S ranging from the lowest possible value up to the 95th percentile value.

12.1.4 Monotone-constrained nonparametric approach

The controlled risk analyses for each group HIPP, VIPP, HIPP PWH, VIPP PWH, are repeated using Avi Kenny's method (PhD dissertation) that was applied to the Moderna COVE booster dose correlates study. It is implemented using the *vaccine* R package. Analyses of relative risk are not done with this approach.

12.1.5 Plotting results for controlled risk and controlled relative risk analyses

For each immune marker at M0, peak level, and fold-rise, the following output is planned:

1. Cox-model method plotting of point, 95% CI, and 95% EUI estimates of each of $P(Y(1, s) = 1)$ and $P(Y(0, s) = 1)$ in side by side panels or on the same panel
2. Cox-model method plotting of point, 95% CI, and 95% EUI estimates of each of $P(Y(1, s) = 1)/P(Y(0) = 1)$ and $P(Y(0, s) = 1)/P(Y(1) = 1)$ in side by side panels or on the same panel
3. Nonparametric monotone-constrained method plotting of point, 95% CI, and 95% EUI estimates of each of $P(Y(1, s) = 1)$ and $P(Y(0, s) = 1)$ in side by side panels or on the same panel
4. Nonparametric monotone-constrained method plotting of point and 95% CI estimates of each of $P(Y(1, s) = 1)/P(Y(0) = 1)$ and $P(Y(0, s) = 1)/P(Y(1) = 1)$ in side by side panels or on the same panel

12.2 CoP: Stochastic Interventional Thresholded Effects on Risk and Relative Risk

An alternative stochastic interventional effects causal CoP analysis is also considered based on thresholding of antibody markers. The thresholded risk parameter of interest for a given cohort (e.g., HIPP) is as defined above in Section 11.4, where van der Laan et al. (2023) explains the

causal interpretation of this parameter and the assumptions needed for the statistical parameter to equate to the causal parameter of interest. In addition, as described in the previous section, the thresholded relative risk is also estimated, where this parameter is the above-described thresholded risk for a given cohort (e.g., HIPP) divided by the overall risk for the comparator group (i.e., VIPP). The efficient influence functions and the delta method are used to obtain 95% confidence intervals about the thresholded relative risk.

13 Summary of the Set of CoR and CoP Analyses and Their Requirements and Contingencies, and Synthesis of the Results, Including Reconciling Any Possible Contradictions in Results

Table 7 summarizes all of the correlates analyses of peak level (and fold-rise) markers that are done, including contingencies for whether and when each analysis is done. For the correlates analyses of antibody markers, enough cases are expected. For the correlates analyses of cellular markers, this table has influence on what analyses are done.

Most of the analyses focus on univariate peak markers. The primary reason to do this is the goal to identify a parsimonious correlate based on a single marker without needing to run the set of assays, and secondary reasons are: (1) the assay readouts are expected to be moderately to highly correlated, and (2) there is ample precedent for univariate markers being accepted as immunological surrogate endpoints for approved vaccines (Plotkin, 2010).

Table 7: Summary of Stage 1 Peak Marker CoR and CoP Analyses with Requirements/Contingencies for Conduct of the Analysis

Analysis	Structure of Peak Marker(s)	Requirements/Contingencies Min No. Vaccine Endpoints	Other
CoR Cox Model	Tertiles of S^1	25	None
	Quant. $S = s^2$	25	None
	Quant. $S \geq s^1$	25	None
CoR Nonpar. threshold	Quant. $S \geq s^1$	35	None
CoR Superlearner[3]	Quant. $S = s$, 2FR, 4FR	35	None
CoP: Correlates of RR	Binary S	35	None
	Quant. $S = s$	35	BIP with $R^2 \geq 0.25$
CoP: Controlled RR	Quant. $S = s$	35	Feasibility of positivity[4]
	Tertiles of $S = s$	35	Feasibility of positivity[4]
CoP: Stoch. Interv. RR	Quant. $S = s$	35	Feasibility of positivity[4]

[1]These analyses are harmonized in addressing the same scientific question of how does endpoint risk vary over vaccinated subgroups defined by S above a threshold.
[2]These exploratory supportive analyses are harmonized in addressing the same scientific question of how does endpoint risk vary over vaccinated subgroups defined by S equal to a given marker value.
[3]Only this Superlearner analysis uses data from multiple assays and multiple readouts as input features; the other analyses consider one peak marker at a time. [4]The positivity assumptions are as follows. Controlled RR: $P(S = s \mid A = 1, X) > 0$ almost surely. Stochastic Interventional RR: $s_i \in \mathcal{S} \implies s_i + \delta \in \mathcal{S} \mid A = 1, X = x$ for all $x \in \mathcal{X}$ and $i = 1, \ldots n$. Mediators of RR: $P(S = s \mid A = 1, X) > 0$ almost surely and $P(S = s|A = 0, X = x) >$ implies $P(S = s|A = 1, X = x) > 0$. The quantitative analysis will require that the largest value S observed in the VIPP arm is larger than the smallest value of S observed in the HIPP arm. This assumption would naturally be satisfied for the tertiles analysis. For quantitative S, the assumption is weaker for the Stochastic Interventional RR analysis, such that it is possible that only this analysis of the three will be done.

Some of the analyses include parametric assumptions for characterizing associations (Cox model and threshold analyses, Cox model versions of Controlled RR analyses) and others are nonparametric or approximately so (all other analyses). If parametric and nonparametric analyses of the same type (e.g., Cox model vs. nonparametric CoR analysis of the same association parameter; Controlled RR Cox model vs. nonparametric monotone dose-response) suggest contradictory results, then the interpretation from the nonparametric analysis will be prioritized, given it is more robust and less likely to be an incorrect result. The diagnostic testing of the parametric assumptions will aid this interpretation. As noted above, if the nonparametric analysis suggesting a contradictory result requires a positivity assumption, then its results will only be prioritized if diagnostics support feasibility of the positivity assumption.

References

Benkeser, D., Montefiori, D.C., McDermott, A.B., Fong, Y., Janes, H.E., Deng, W. et al (2023), "Comparing antibody assays as correlates of protection against COVID-19 in the COVE mRNA-1273 vaccine efficacy trial," *Science translational medicine*, 15, eade9078.

Breslow, N., Lumley, T., Ballantyne, C., Chambless, L. and Kulich, M. (2009a), "Improved Horvitz-Thompson Estimation of Model Parameters from Two-phase Stratified Samples: Applications in Epidemiology," *Statistical Biosciences*, 1, 32–49.

Breslow, N., Lumley, T., Ballantyne, C., Chambless, L. and Kulich, M. (2009b), "Using the whole cohort in the analysis of case-cohort data." *American Journal of Epidemiology*, 169, 1398–1405.

Ding, P. and VanderWeele, T. (2016), "Sensitivity analysis without assumptions," *Epidemiology*, 27(3), 368.

Donovan, K., Hudgens, M. and Gilbert, P.B. (2019), "Nonparametric inference for immune response thresholds of risk in vaccine studies," *Annals of Applied Statistics*, 13, 1147–1165, PMCID: PMC6613658 [Delayed release (embargo): Available on 2020-06-01].

Etievant, L. and Gail, M.H. (2024), "CaseCohortCoxSurvival: an R Package for Case-Cohort Inference for Relative Hazard and Pure Risk under the Cox Model," *arXiv preprint arXiv:2402.08744*.

Garrett, N., Tapley, A., Hudson, A., Dadabhai, S., Zhang, B., Mgodi, N.M. et al (2025), "Hybrid versus vaccine immunity of mRNA-1273 among people living with HIV in East and Southern Africa: a prospective cohort analysis from the multicentre CoVPN 3008 (Ubuntu) study," *eClinicalMedicine*, 80, 103054.

Gilbert, P.B., Montefiori, D.C., McDermott, A.B., Fong, Y., Benkeser, D., Deng, W. et al (2022), "Immune correlates analysis of the mRNA-1273 COVID-19 vaccine efficacy clinical trial," *Science*, 375, 43–50.

Gilbert, P.B., Fong, Y., Kenny, A. and Carone, M. (2023), "A controlled effects approach to assessing immune correlates of protection," *Biostatistics*, 24, 850–865.

Gilbert, P.B., Fong, Y., Hejazi, N.S., Kenny, A., Huang, Y., Carone, M. et al (2024), "Four statistical frameworks for assessing an immune correlate of protection (surrogate endpoint) from a randomized, controlled, vaccine efficacy trial," *Vaccine*.

Lumley, T. (2010), *Complex surveys: a guide to analysis using R*, vol. 565, John Wiley & Sons.

Lumley, T. and Scott, A. (2013), "Partial likelihood ratio tests for the Cox model under complex sampling," *Statistics in Medicine*, 32, 110–123.

Plotkin, S.A. (2010), "Correlates of Protection Induced by Vaccination." *Clinical Vaccine Immunology*, 17, 1055–1065.

Prentice, R. (1986), "A case-cohort design for epidemiologic cohort studies and disease prevention trials." *Biometrika*, 73, 1–11.

van der Laan, L., Zhang, W. and Gilbert, P.B. (2023), "Nonparametric estimation of the causal effect of a stochastic threshold-based intervention," *Biometrics*, 79, 1014–1028.

VanderWeele, T. and Ding, P. (2017), "Sensitivity analysis in observational research: introducing the E-value," *Annals of Internal Medicine*, 167(4), 268–74.

VanderWeele, T. and Mathur, M. (2020), "Commentary: developing best-practice guidelines for the reporting of E-values," *International Journal of Epidemiology*, 49(5), 1495–1497.

MEMORANDUM

**Date:**	August 01, 2023
**To:**	David Montefiori, Nono Mkhize
**From:**	Jin Kee, Yunda Huang
**RE:**	SARS-CoV-2 Neutralizing Antibody Assay Comparisons between Duke and NICD Labs using Samples from CoVPN 3008

EXECUTIVE SUMMARY

This report summarizes the comparison of neutralization antibody responses measured by two neutralizing antibody (nAb) assays using samples collected from CoVPN 3008. One of the assays is the SARS ACE-2 nAb assay performed at the Duke lab, and the other is the VSV-based nAb assay performed at the NICD lab. Overall, we found that there does not appear to be a simple multiplicative factor to convert the two assay readouts to the same scale. More advanced calibration methods may be needed and should be discussed.

Specifically, responses to the D614G and BA.4/5 variants were measured by both assays using samples collected at Month 0 and Month 1 (or Month 2), corresponding to baseline and 1 month after the last pre-Month 6 vaccination for 38 HIV-1 negative participants in the immunogenicity subset from CoVPN 3008. Titers not quantifiable below the initial starting dilution (1:10) were set to 5. A total of 152 samples were assayed by both assays and compared: 38 participants x 2 timepoints (Month 0 and Month 1 or Month 0 and Month 2) x 2 variants (D614G and BA.4/5).

In summary, ID50 and ID80 titers between Duke and NICD were highly correlated with a linear correlation coefficient > 0.8 but with systemic shifts in levels, especially for D614G.

ID50 and ID80 titers between Duke and NICD were highly correlated with a linear correlation coefficient > 0.8 but with systemic shifts in levels for D614G.
ID50 for D614G
- Average fold change of NICD/Duke at Month 0 is 4.19 (range: 0.79, 22.12)
- Average fold change of NICD/Duke at Month 1 or 2 is 4.96 (range: 0.10, 10.55)

ID80 for D614G
- Average fold change of NICD/Duke at Month 0 is 2.67 (range: 0.68, 12.75)
- Average fold change of NICD/Duke at Month 1 or 2 is 3.79 (range: 0.18, 8.32)

ID50 for BA.4/5
- Average fold change of NICD/Duke at Month 0 is 1.65 (range: 0.07, 8.54)
- Average fold change of NICD/Duke at Month 1 or 2 is 1.85 (range: 0.56, 6.92)

ID80 for BA.4/5

1100 Fairview Ave N - LE-400 - PO Box 19024 - Seattle, WA 98109-1024 - (206) 667-7000 - (206) 667-4812 Fax

- Average fold change of NICD/Duke at Month 0 is 1.09 (range: 0.09, 2.66)
- Average fold change of NICD/Duke at Month 1 or 2 is 1.18 (range: 0.51, 3.36)

The concordance correlation coefficient (CCC) is a combined measure of linear correlation and agreement accuracy. We also found that ID50 and ID80 titers for BA.4/5 between Duke and NICD were highly concordant with a CCC > 0.9. ID50 and ID80 titers for D614G between Duke and NICD were reasonably concordant with CCC > 0.8 at Month 0, but less concordant at Month 1 or 2, mainly due to the shift in levels.

ID50 for D614G
- CCC between NICD and Duke at Month 0 is 0.84 (95% CI: 0.74, 0.91)
- CCC between NICD and Duke at Month 1 or 2 is 0.53 (95% CI: 0.38, 0.66)

ID80 for D614G
- CCC between NICD and Duke at Month 0 is 0.89 (95% CI: 0.82, 0.93)
- CCC between NICD and Duke at Month 1 or 2 is 0.64 (95% CI: 0.49, 0.74)

ID50 for BA.4/5
- CCC between NICD and Duke at Month 0 is 0.93 (95% CI: 0.87, 0.96)
- CCC between NICD and Duke at Month 1 or 2 is 0.91 (95% CI: 0.83, 0.95)

ID80 for BA.4/5
- CCC between NICD and Duke at Month 0 is 0.95 (95% CI: 0.91, 0.97)
- CCC between NICD and Duke at Month 1 or 2 is 0.97 (95% CI: 0.94, 0.98)

Table 1: Titer Summary for Duke and NICD Data

Titer	Antigen	Month	Lab	N	Min	25%ile	Median	75%ile	Max	Mean	Geometric Mean	SD
ID50	D614G	0	Duke	38	5.0	13.7	182.9	653.4	3067.5	544.4	111.2	860.0
			NICD	38	5.0	62.1	383.1	1995.6	20706.5	2198.3	318.3	4386.2
		1	Duke	25	293.5	2243.7	3159.6	5839.2	16737.5	5092.9	3436.5	4818.3
			NICD	25	659.7	12368.4	20673.6	34207.5	111456.1	27132.0	18077.7	24272.1
		2	Duke	13	319.8	816.2	1655.5	4042.6	18965.4	3630.3	1765.4	5155.6
			NICD	13	33.5	2038.2	4015.4	16031.4	56974.2	13171.3	4128.4	18301.5
	BA.4/5	0	Duke	38	5.0	5.0	74.2	654.5	3649.1	453.9	76.9	775.0
			NICD	38	5.0	18.2	130.4	363.7	6163.1	486.3	96.3	1161.1
		1	Duke	25	116.8	1781.0	2504.7	3256.5	39804.4	4312.3	2446.3	7663.1
			NICD	25	808.7	2003.7	3079.8	5764.4	37273.6	5849.8	3469.4	7767.4
		2	Duke	13	5.0	43.5	521.9	1536.3	6544.4	1267.9	324.5	1835.2
			NICD	13	30.4	66.1	1132.6	1795.5	17071.8	2187.9	545.4	4533.6
ID80	D614G	0	Duke	38	5.0	5.0	62.2	254.9	1337.1	191.7	49.4	300.9
			NICD	38	5.0	6.3	130.7	633.0	5441.0	654.3	101.0	1254.3
		1	Duke	25	88.3	773.4	1626.2	2294.7	8455.8	2045.4	1390.3	1936.5
			NICD	25	290.0	3574.7	7616.1	9609.8	24798.1	8246.0	5661.5	6506.0
		2	Duke	13	88.0	319.6	478.5	1812.2	7555.8	1337.4	615.4	2021.0
			NICD	13	16.0	480.0	1056.4	5674.6	15207.3	3897.7	1252.6	5147.8
	BA.4/5	0	Duke	38	5.0	5.0	19.9	211.4	1431.8	148.6	33.8	273.2
			NICD	38	5.0	5.0	31.5	125.5	1667.7	136.3	33.0	314.6
		1	Duke	25	54.9	547.3	1080.6	1457.5	11935.9	1491.1	886.3	2280.4
			NICD	25	165.5	568.8	909.1	1227.0	11420.7	1594.6	938.7	2303.4
		2	Duke	13	5.0	13.2	261.3	457.5	2152.9	436.2	129.5	606.9
			NICD	13	5.0	14.8	284.0	485.5	2655.9	455.2	142.8	698.3

Table 2: Titer Summary for Duke and NICD Data (Positive Responders Only)

Titer	Antigen	Month	Lab	N	Min	25%ile	Median	75%ile	Max	Mean	Geometric Mean	SD
ID50	D614G	0	Duke	29	11.2	125.5	226.0	768.7	3067.5	711.7	291.3	924.5
			NICD	34	15.7	142.6	478.9	3391.5	20706.5	2456.4	518.9	4573.8
		1	Duke	25	293.5	2243.7	3159.6	5839.2	16737.5	5092.9	3436.5	4818.3
			NICD	25	659.7	12368.4	20673.6	34207.5	111456.1	27132.0	18077.7	24272.1
		2	Duke	13	319.8	816.2	1655.5	4042.6	18965.4	3630.3	1765.4	5155.6
			NICD	13	33.5	2038.2	4015.4	16031.4	56974.2	13171.3	4128.4	18301.5
	BA.4/5	0	Duke	27	13.5	59.5	209.1	995.6	3649.1	636.8	234.4	857.1
			NICD	33	10.6	42.7	155.8	534.5	6163.1	559.2	150.7	1231.7
		1	Duke	25	116.8	1781.0	2504.7	3256.5	39804.4	4312.3	2446.3	7663.1
			NICD	25	808.7	2003.7	3079.8	5764.4	37273.6	5849.8	3469.4	7767.4
		2	Duke	12	22.2	262.1	658.5	1781.5	6544.4	1373.2	459.5	1875.3
			NICD	13	30.4	66.1	1132.6	1795.5	17071.8	2187.9	545.4	4533.6
ID80	D614G	0	Duke	26	16.7	56.7	143.2	354.1	1337.1	277.8	142.2	330.9
			NICD	28	10.2	95.4	299.3	857.0	5441.0	886.2	295.7	1394.3
		1	Duke	25	88.3	773.4	1626.2	2294.7	8455.8	2045.4	1390.3	1936.5
			NICD	25	290.0	3574.7	7616.1	9609.8	24798.1	8246.0	5661.5	6506.0
		2	Duke	13	88.0	319.6	478.5	1812.2	7555.8	1337.4	615.4	2021.0
			NICD	13	16.0	480.0	1056.4	5674.6	15207.3	3897.7	1252.6	5147.8
	BA.4/5	0	Duke	24	15.7	25.7	104.0	356.8	1431.8	232.4	103.2	316.5
			NICD	26	11.0	31.5	61.3	195.5	1667.7	196.9	78.8	366.6
		1	Duke	25	54.9	547.3	1080.6	1457.5	11935.9	1491.1	886.3	2280.4
			NICD	25	165.5	568.8	909.1	1227.0	11420.7	1594.6	938.7	2303.4
		2	Duke	12	10.6	66.8	298.1	533.0	2152.9	472.1	169.8	619.3
			NICD	12	11.3	145.2	305.8	512.3	2655.9	492.7	188.9	715.5

Table 3: Fold Change Summary for Duke and NICD Data

Titer	Fold Change	Antigen	Month	N	Min	25%ile	Median	75%ile	Max	Mean	SD
ID50	NICD/Duke	D614G	0	38	0.79	1.52	2.90	4.74	22.12	4.19	4.71
			1/2	38	0.10	2.93	4.30	6.89	10.55	4.96	2.75
		BA.4/5	0	38	0.07	0.87	1.08	2.03	8.54	1.65	1.48
			1/2	38	0.56	0.95	1.46	2.11	6.92	1.85	1.40
ID80	NICD/Duke	D614G	0	38	0.68	1.00	1.95	3.15	12.75	2.67	2.41
			1/2	38	0.18	2.25	3.37	5.06	8.32	3.79	1.94
		BA.4/5	0	38	0.09	0.85	1.00	1.16	2.66	1.09	0.49
			1/2	38	0.51	0.85	1.00	1.28	3.36	1.18	0.60

Table 4: Fold Change Summary for Duke and NICD Data among Positive Responders for Both Labs Included in Fold Change

Titer	Fold Change	Antigen	Month	N	Min	25%ile	Median	75%ile	Max	Mean	SD
ID50	NICD/Duke	D614G	0	29	0.8	1.8	2.9	5.1	22.1	4.4	5.1
			1/2	38	0.1	2.9	4.3	6.9	10.6	5.0	2.7
		BA.4/5	0	27	0.1	0.8	1.1	1.6	2.8	1.2	0.7
			1/2	37	0.6	0.9	1.4	2.0	6.9	1.7	1.2
ID80	NICD/Duke	D614G	0	26	0.7	1.8	2.2	3.5	12.8	3.1	2.5
			1/2	38	0.2	2.2	3.4	5.1	8.3	3.8	1.9
		BA.4/5	0	24	0.1	0.7	0.9	1.2	2.3	1.0	0.5
			1/2	37	0.5	0.8	1.0	1.3	3.4	1.2	0.6

Table 5: Listing of Titers of Discordant Positive/Negative Samples

Titer	Participant ID	Antigen	Month	Duke	NICD
ID50	340802575	D614G	0	<10.0	24.1975651
	341802567	D614G	0	<10.0	57.6278700
	341802567	BA.4/5	0	<10.0	42.6855119
	342803347	D614G	0	<10.0	22.1215977
	342803347	BA.4/5	0	<10.0	16.1963312
	723800648	D614G	0	<10.0	18.2493853
	723800648	BA.4/5	0	<10.0	12.8021509
	753802562	BA.4/5	2	<10.0	30.4197595
	834802150	BA.4/5	0	<10.0	17.6690661
	873800904	BA.4/5	0	<10.0	10.6074999
	874800378	D614G	0	<10.0	15.6646971
	874800378	BA.4/5	0	<10.0	22.2436589
ID80	340802575	D614G	0	<10.0	10.1752622
	341801849	BA.4/5	0	<10.0	13.3098212
	341802567	BA.4/5	0	<10.0	11.0030732
	342800251	D614G	0	<10.0	36.9769377

Figure 1a: Boxplot of ID50 Titer for D614G

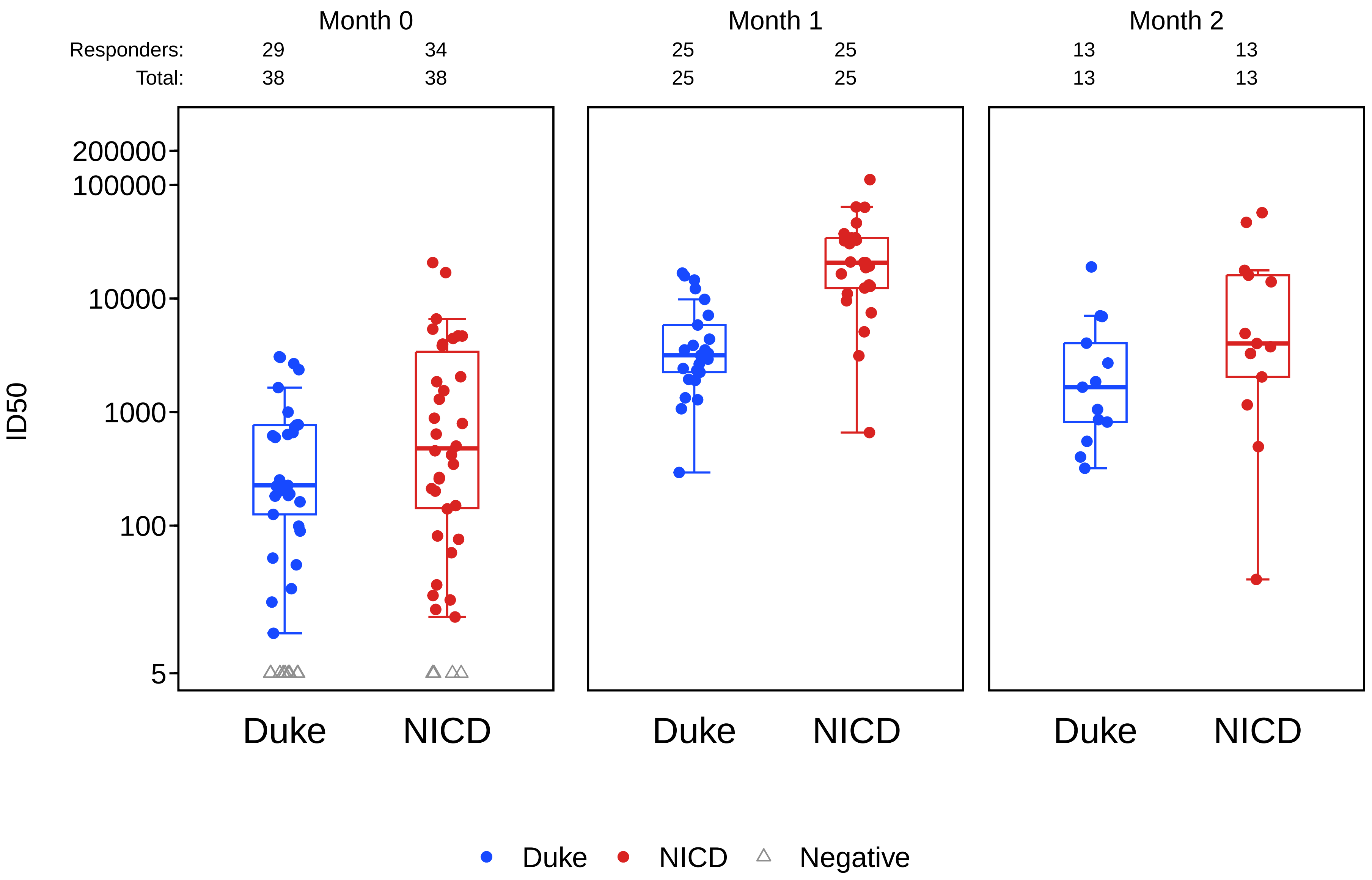

SOURCE: SCHARP jkee2 /trials/covpn/p3008/analysis/adhoc/NAb_concordance/code/create_report.R (Aug 01 12:20 2023)

Figure 1b: Boxplot of ID80 Titer for D614G

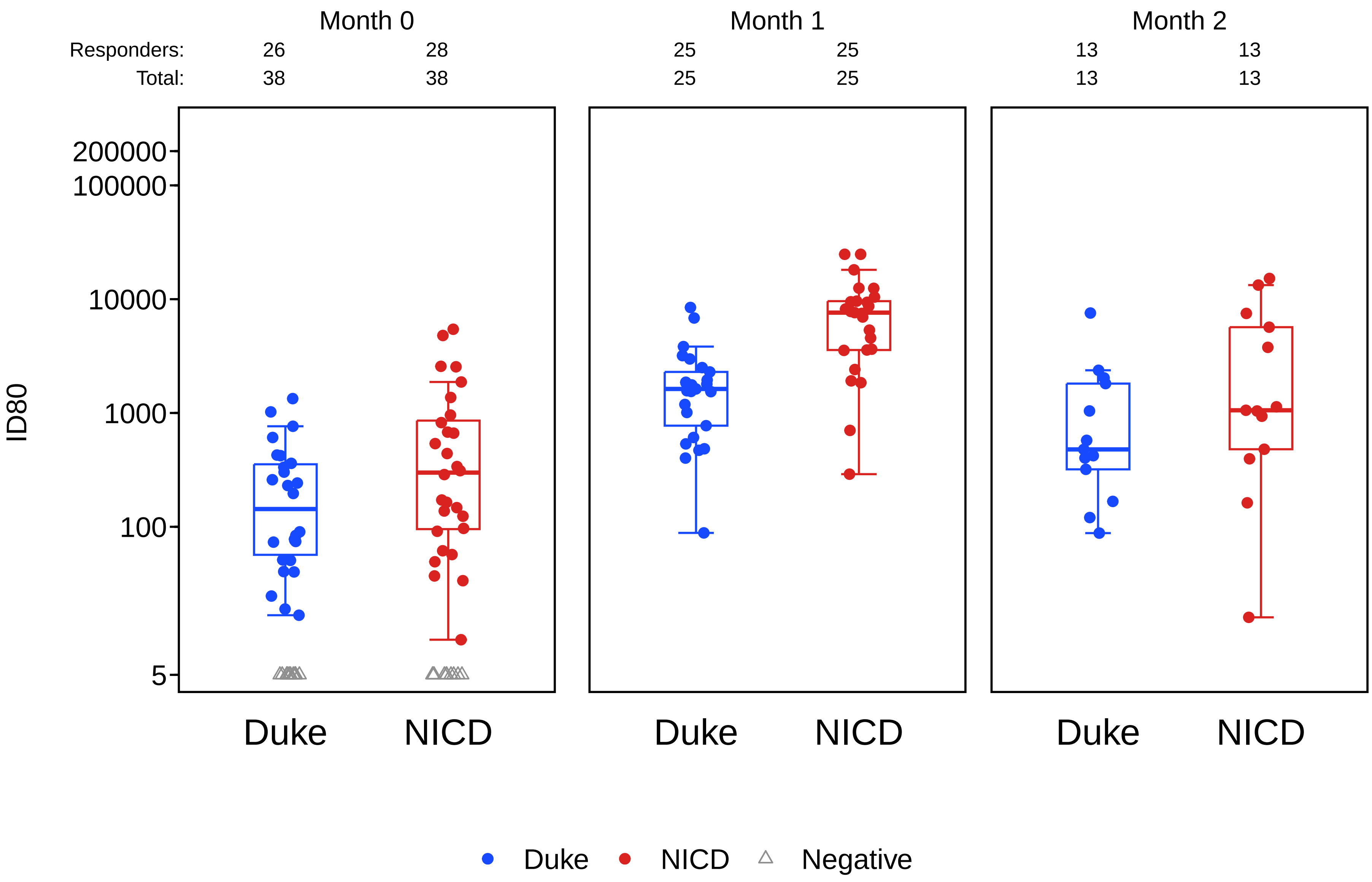

SOURCE: SCHARP jkee2 /trials/covpn/p3008/analysis/adhoc/NAb_concordance/code/create_report.R (Aug 01 12:20 2023)

Figure 1c: Boxplot of ID50 Titer for BA.4/5

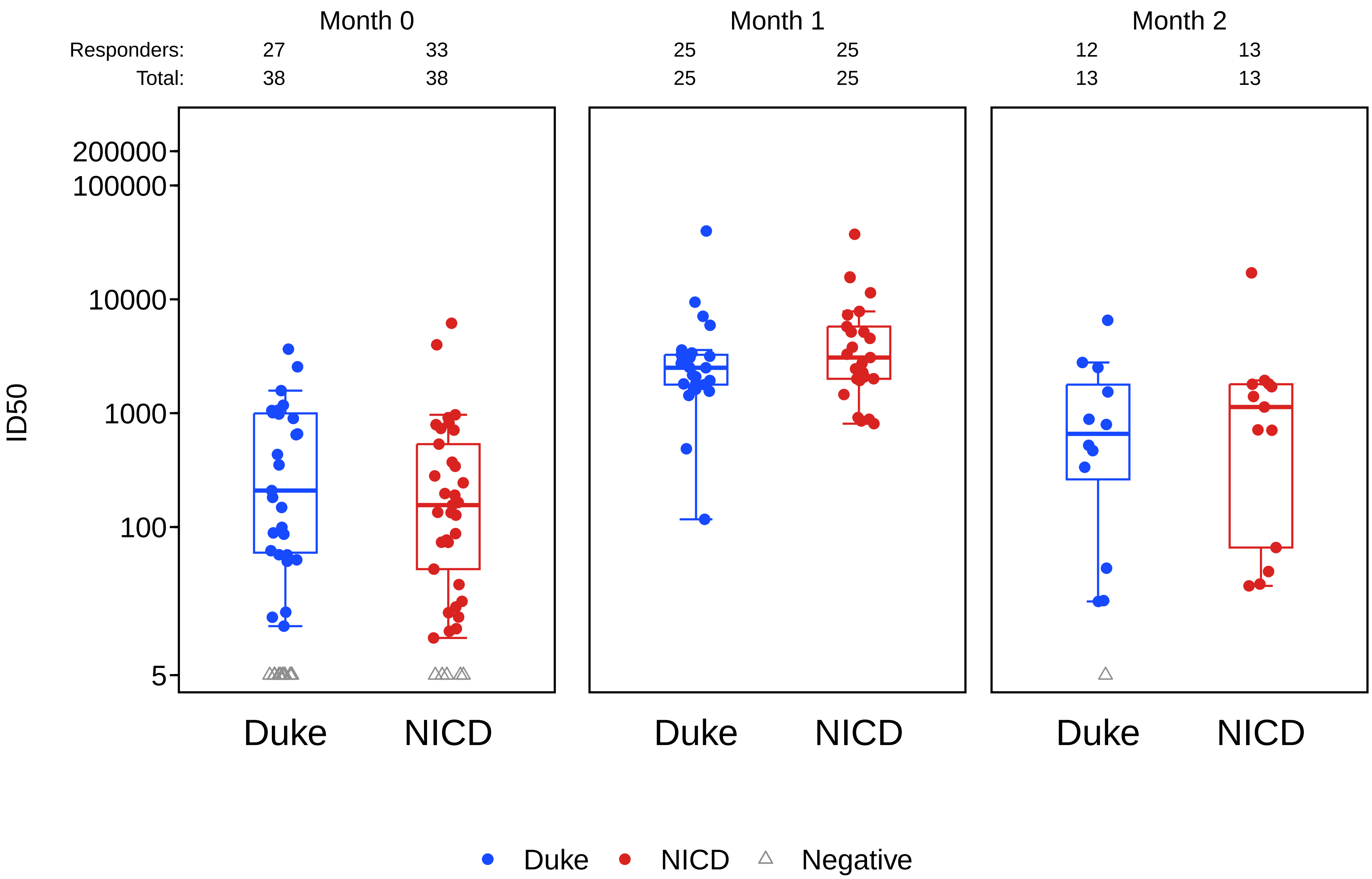

SOURCE: SCHARP jkee2 /trials/covpn/p3008/analysis/adhoc/NAb_concordance/code/create_report.R (Aug 01 12:20 2023)

Figure 1d: Boxplot of ID80 Titer for BA.4/5

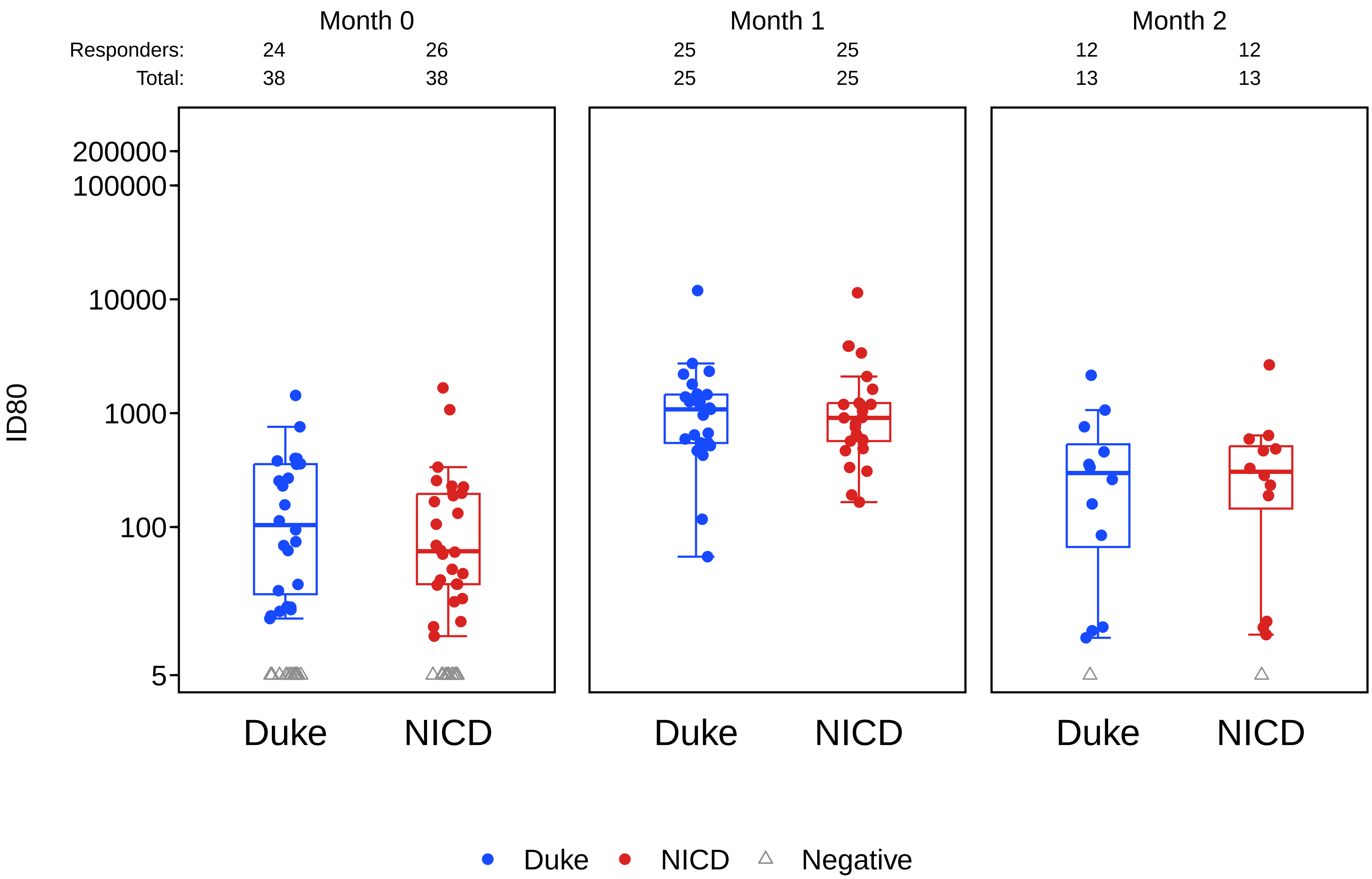

SOURCE: SCHARP jkee2 /trials/covpn/p3008/analysis/adhoc/NAb_concordance/code/create_report.R (Aug 01 12:20 2023)

Figure 2a: Scatterplot of ID50 titer for D614G at Month 0

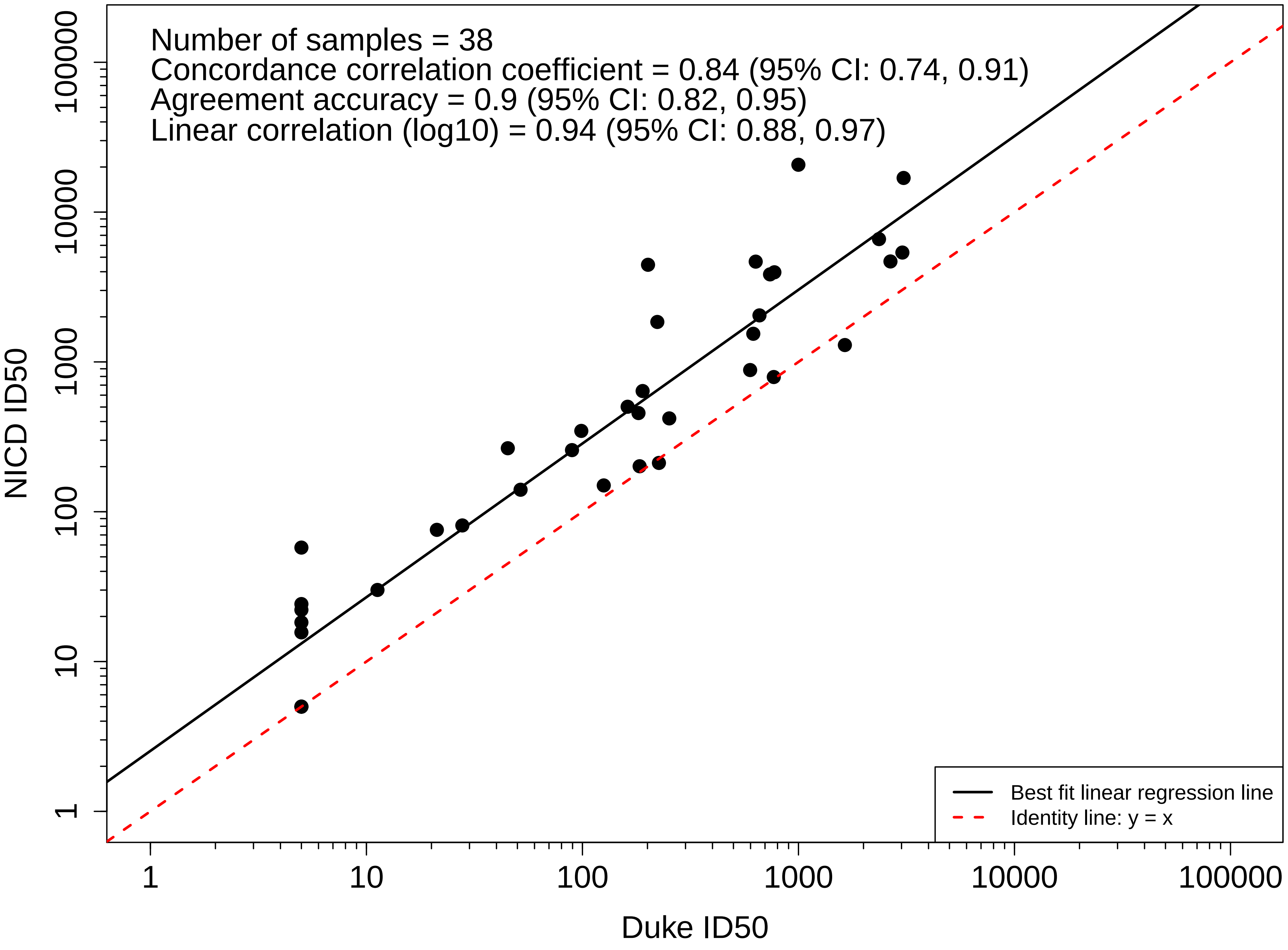

Figure 2b: Scatterplot of ID50 titer for D614G at Months 1/2

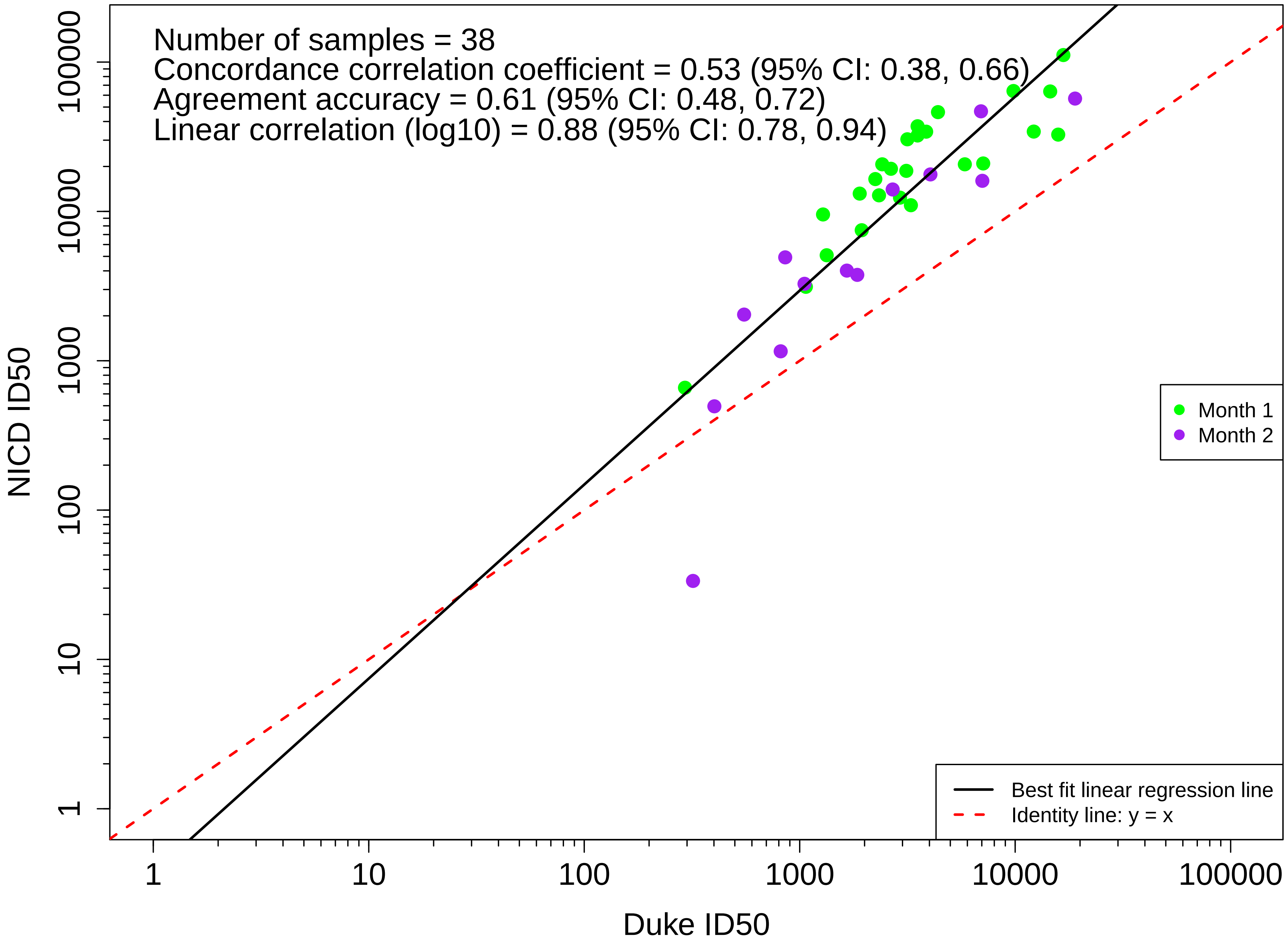

Figure 2c: Scatterplot of ID50 titer for BA.4/5 at Month 0

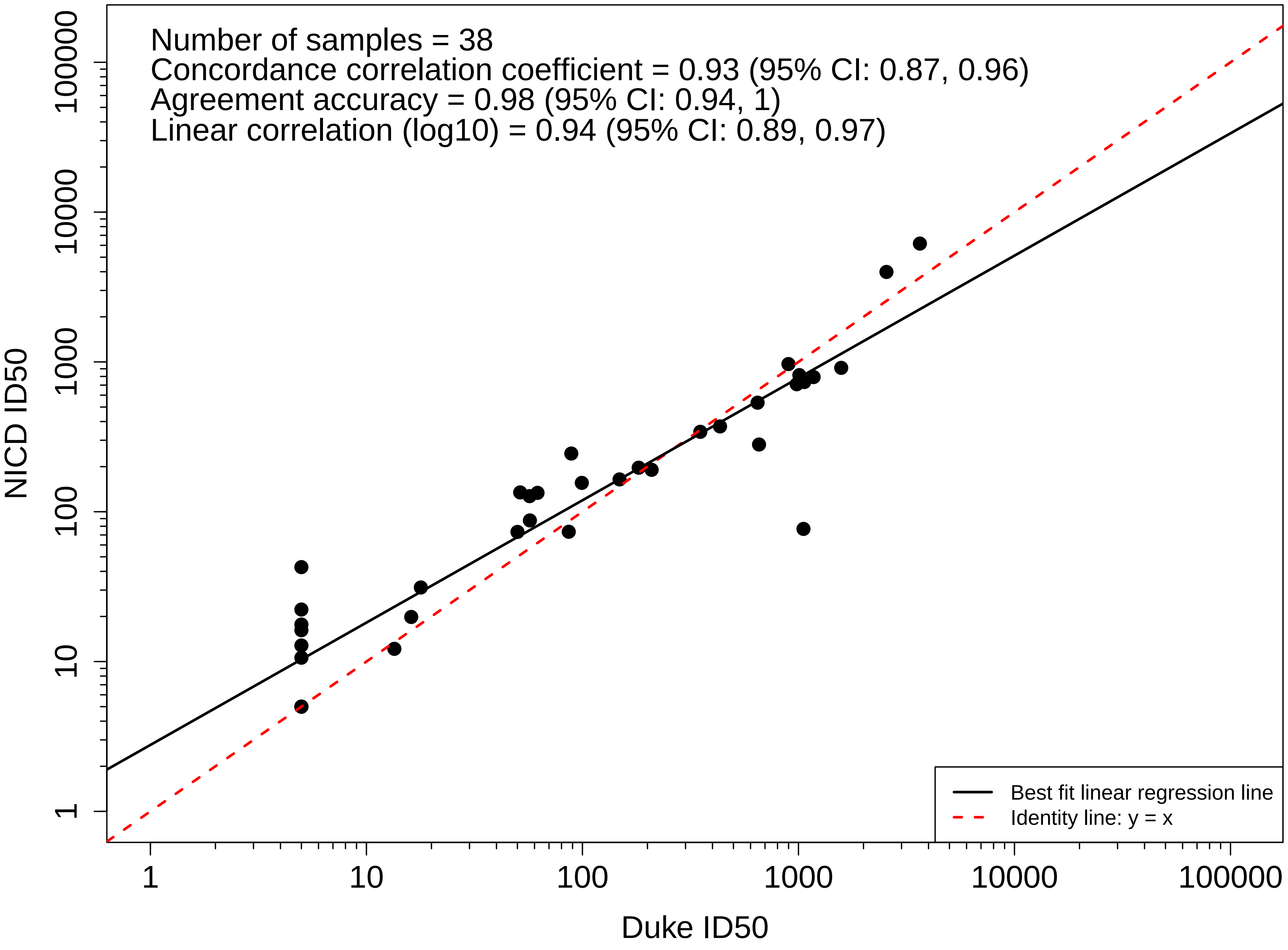

Figure 2d: Scatterplot of ID50 titer for BA.4/5 at Months 1/2

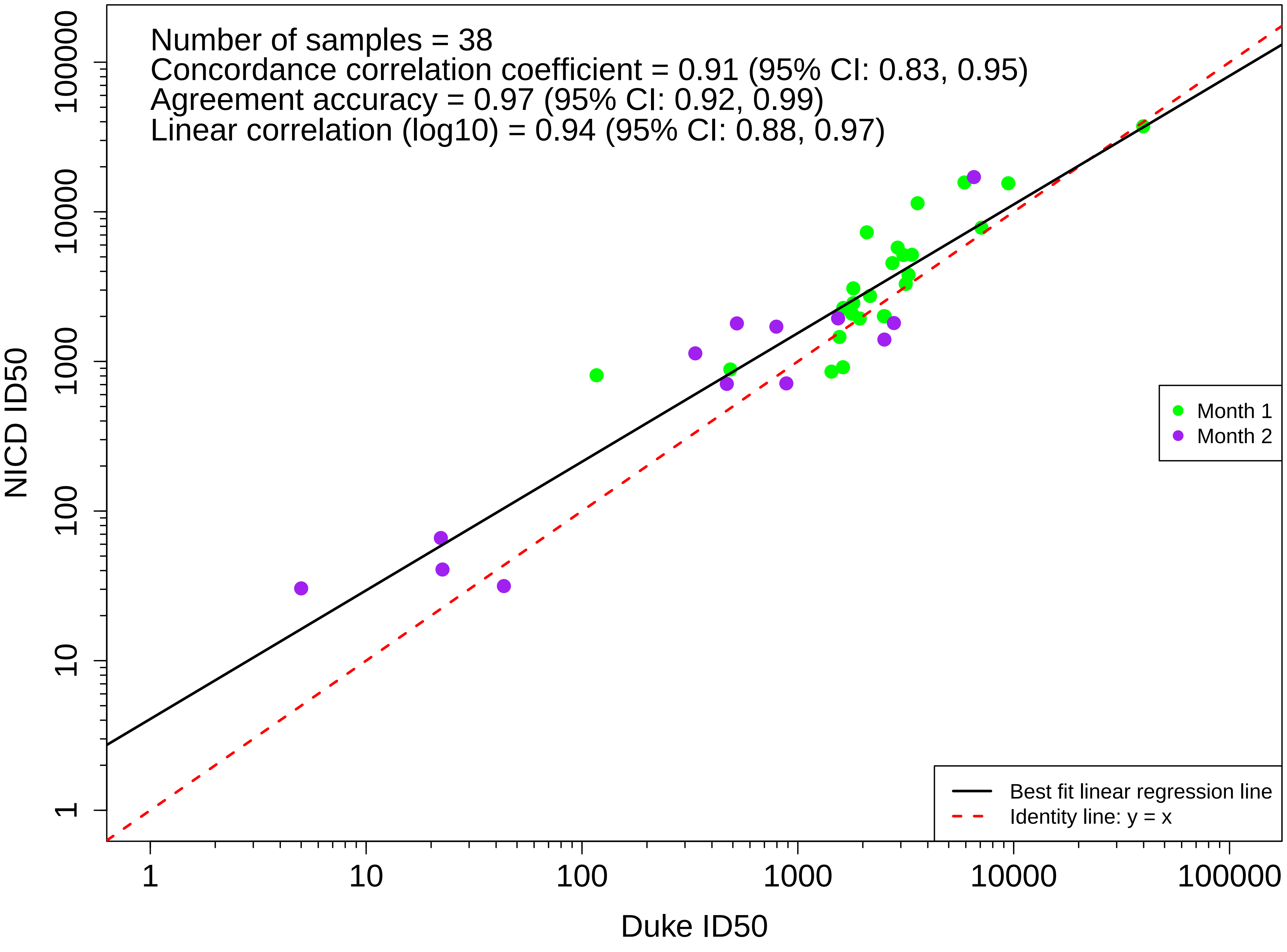

Figure 2e: Scatterplot of ID80 titer for D614G at Month 0

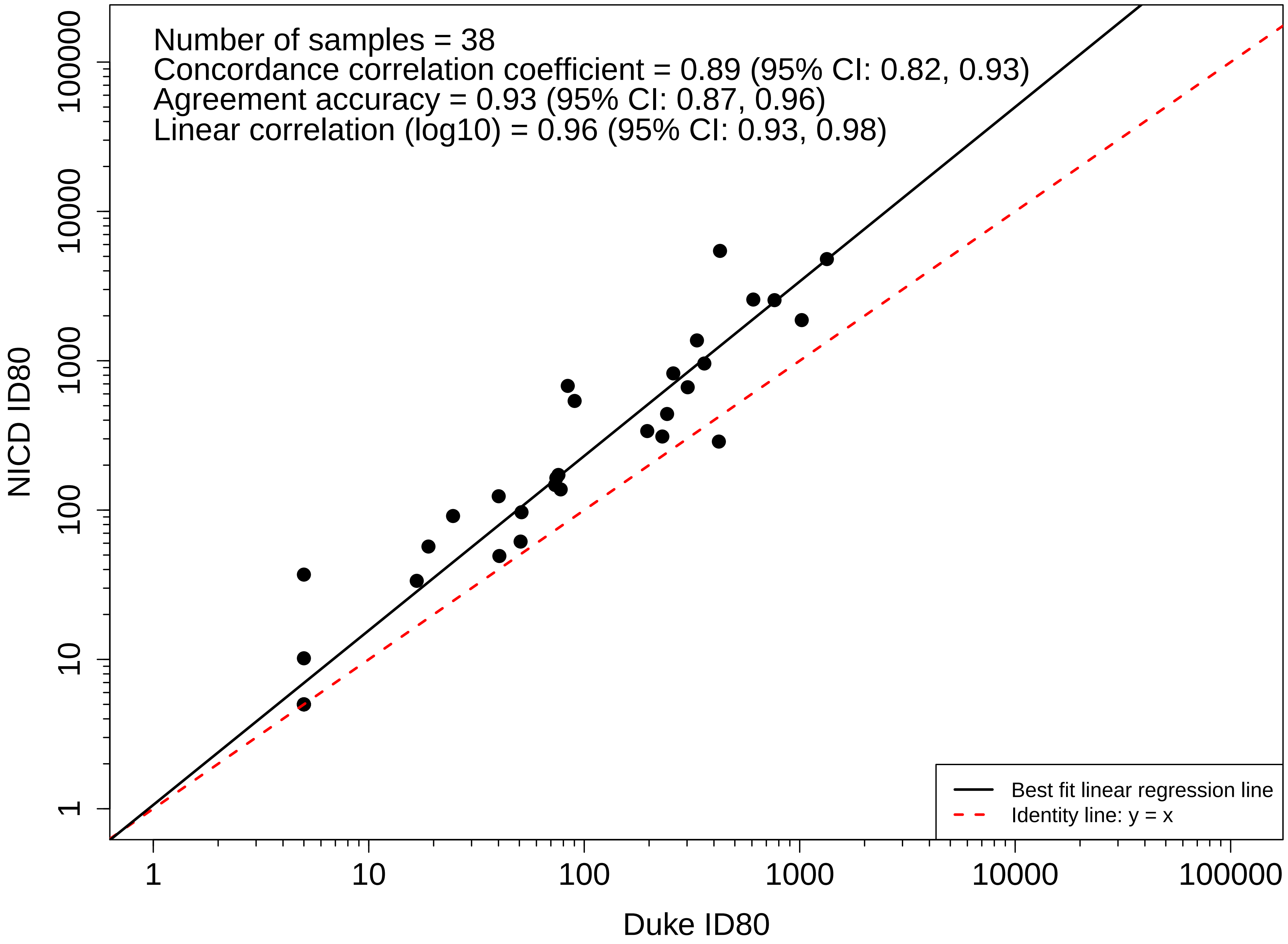

Figure 2f: Scatterplot of ID80 titer for D614G at Months 1/2

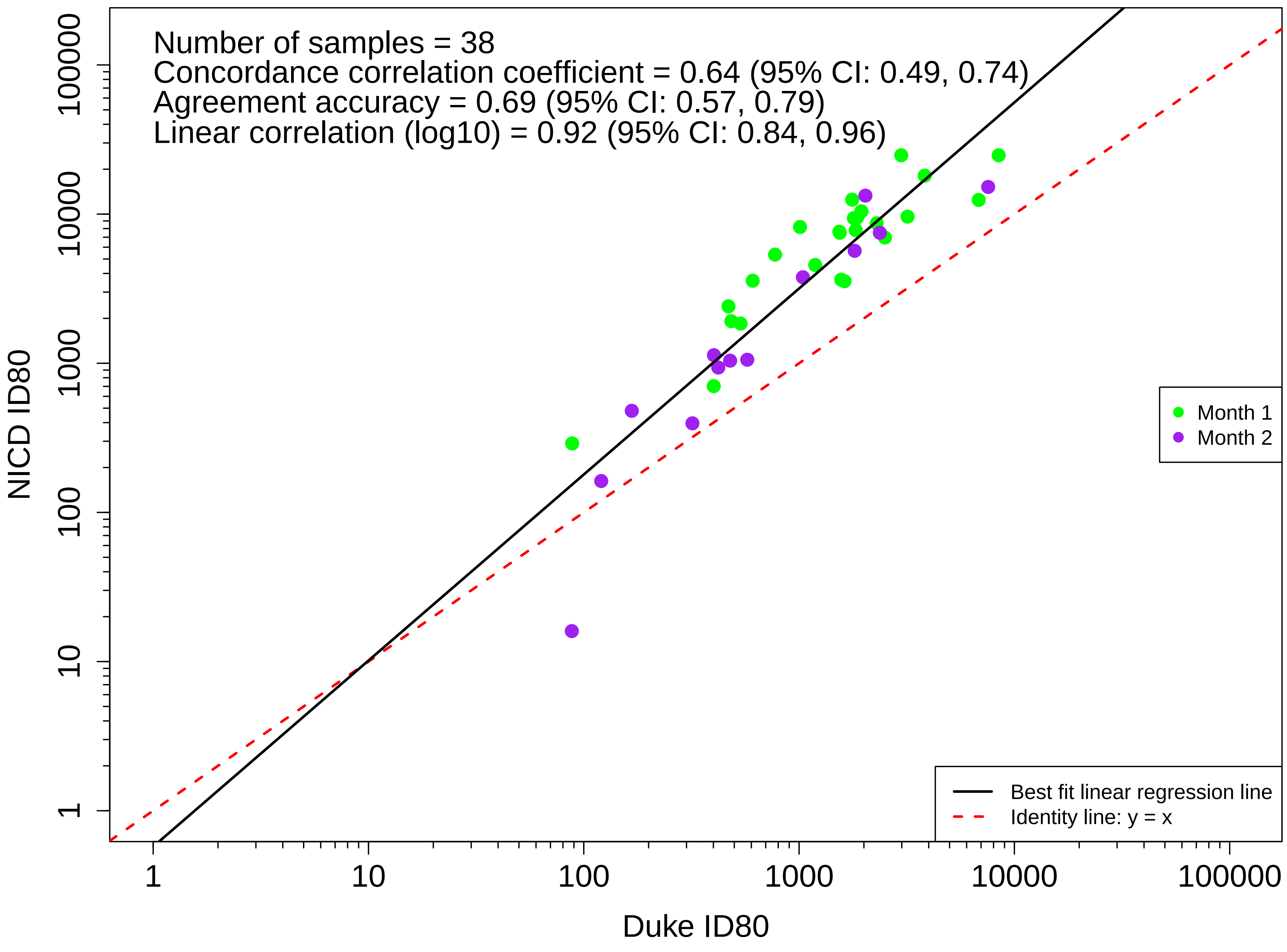

Figure 2g: Scatterplot of ID80 titer for BA.4/5 at Month 0

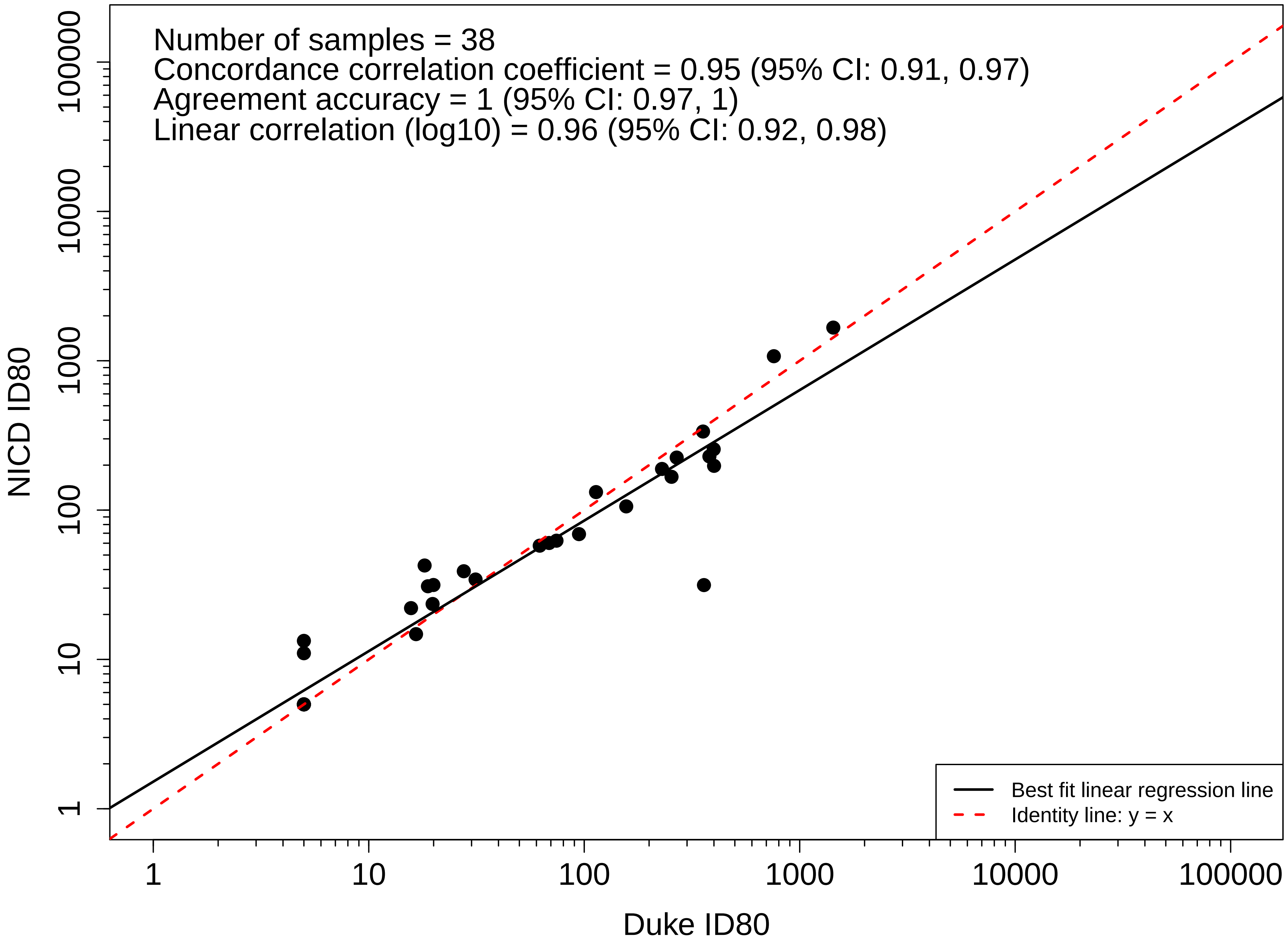

Figure 2h: Scatterplot of ID80 titer for BA.4/5 at Months 1/2

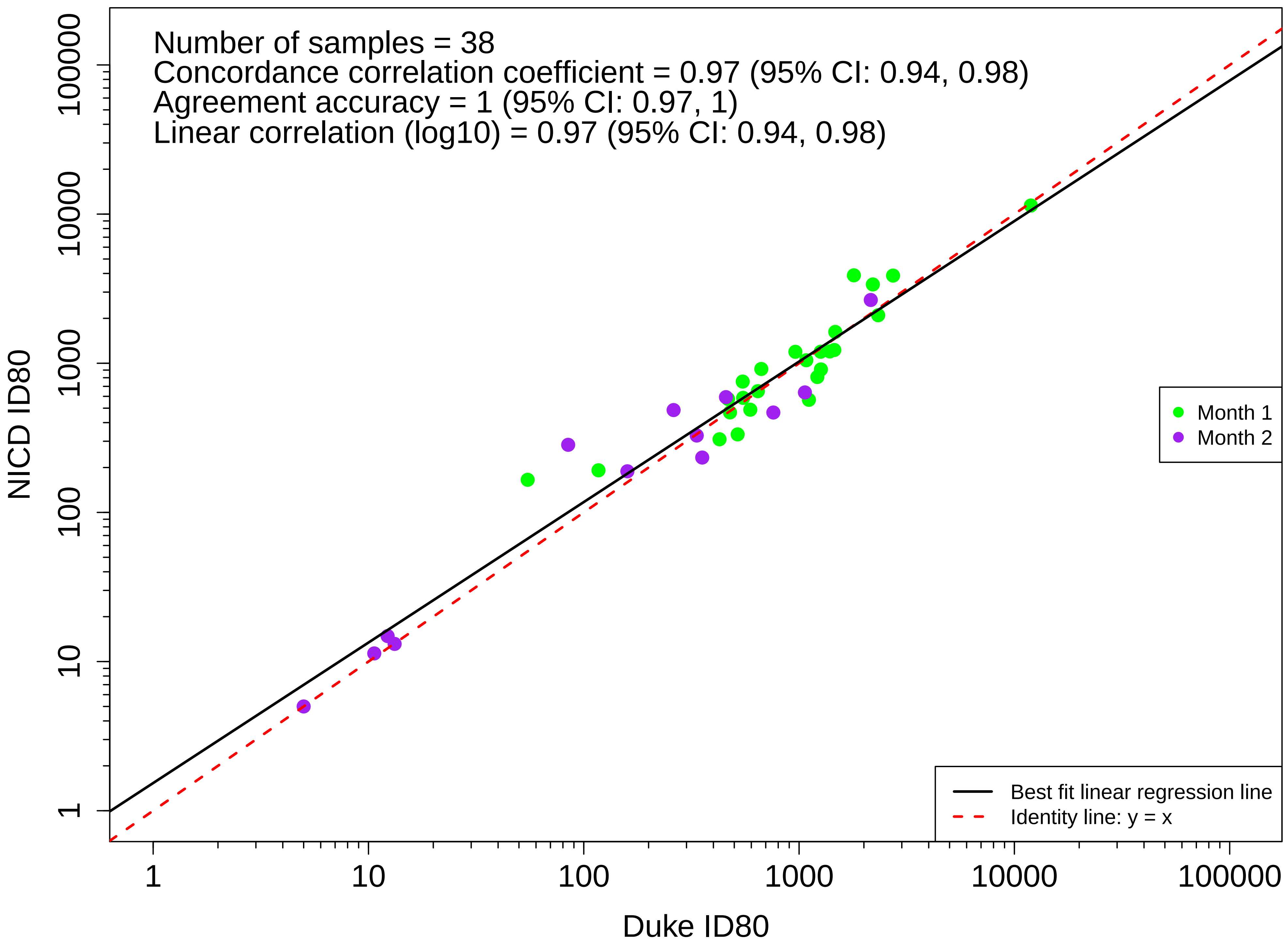

Figure 3a: Bland−Altman plot of ID50 titer for D614G at Month 0

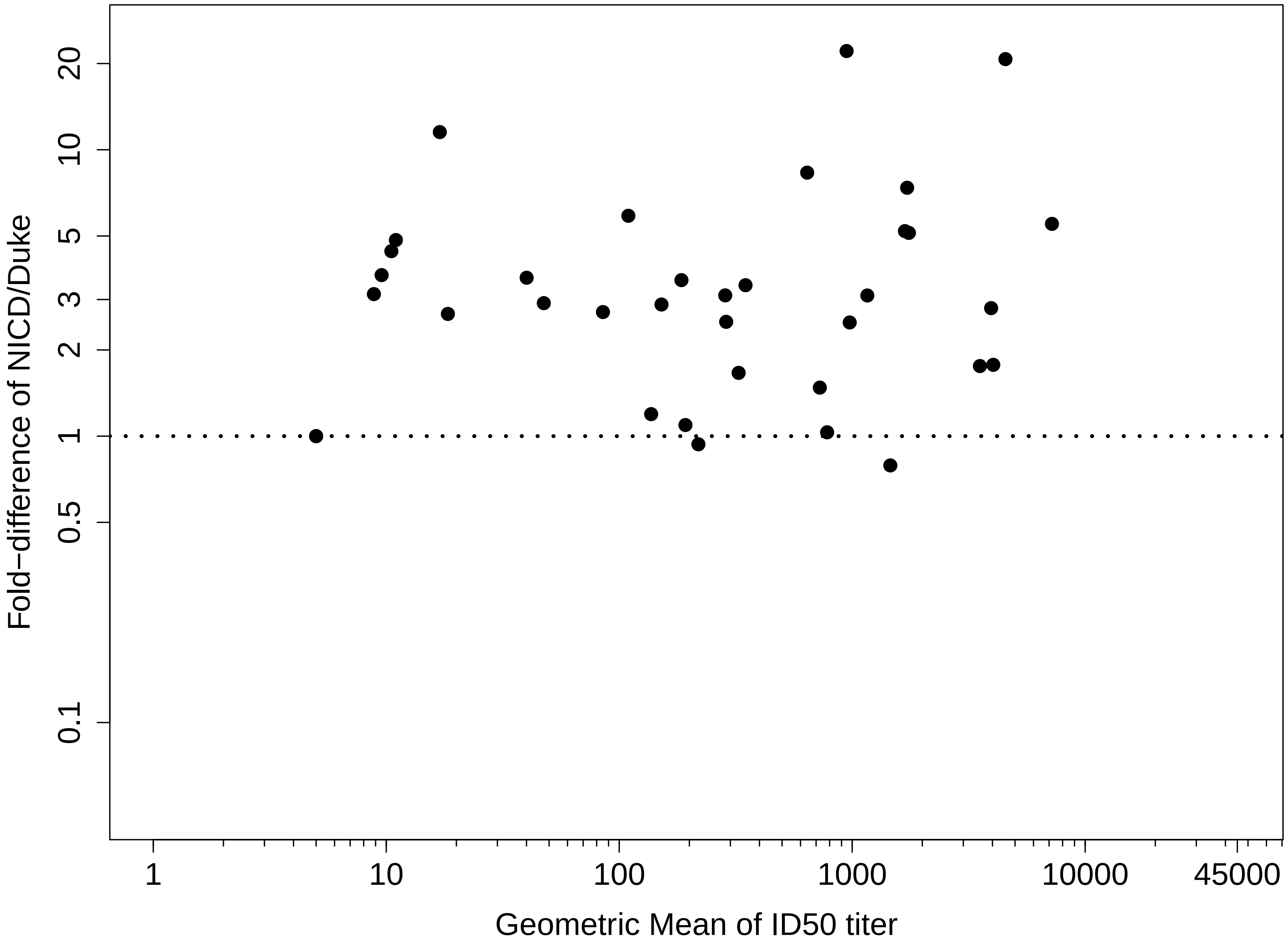

Figure 3b: Bland−Altman plot of ID50 titer for D614G at Months 1/2

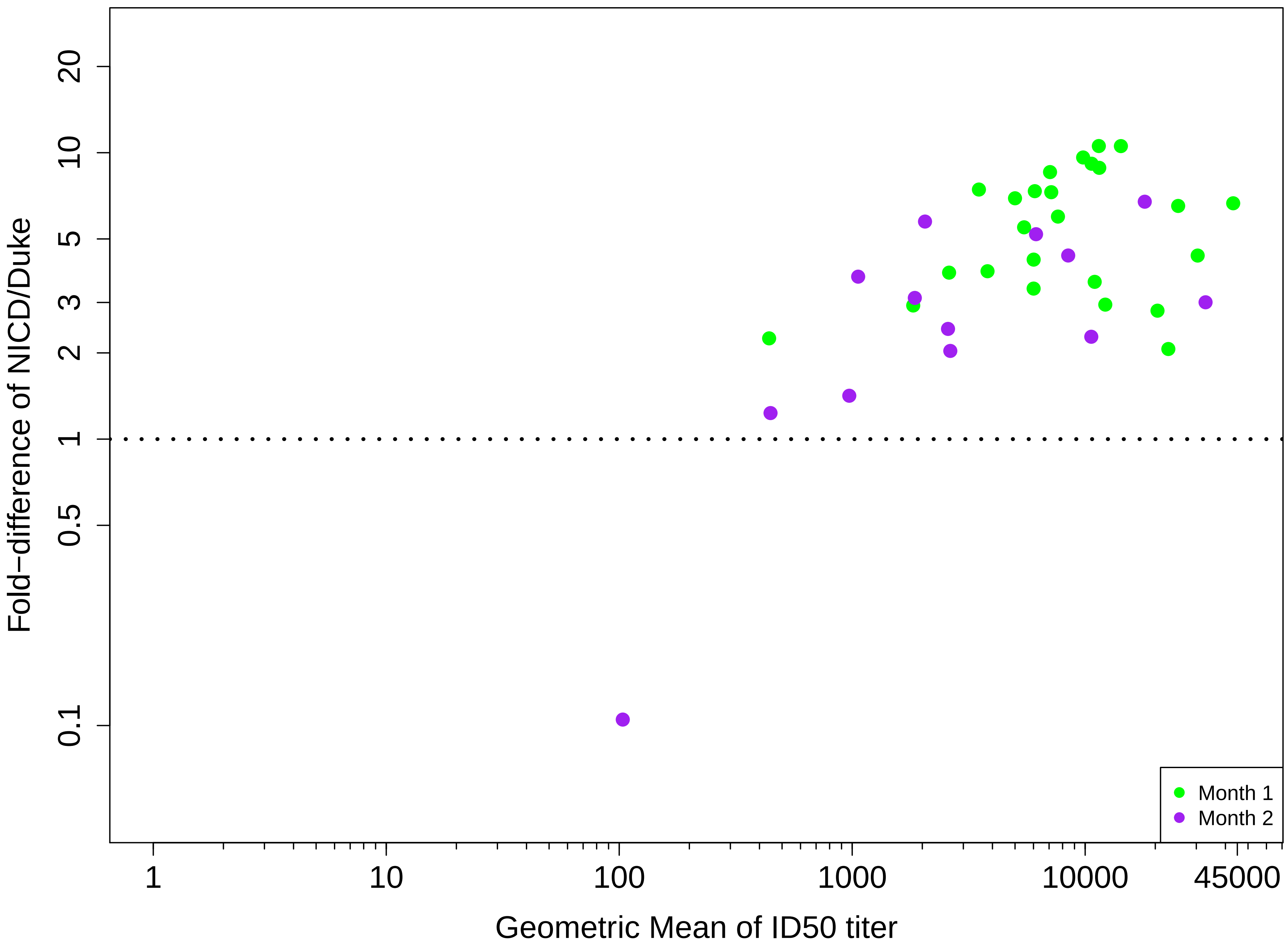

Figure 3c: Bland−Altman plot of ID50 titer for BA.4/5 at Month 0

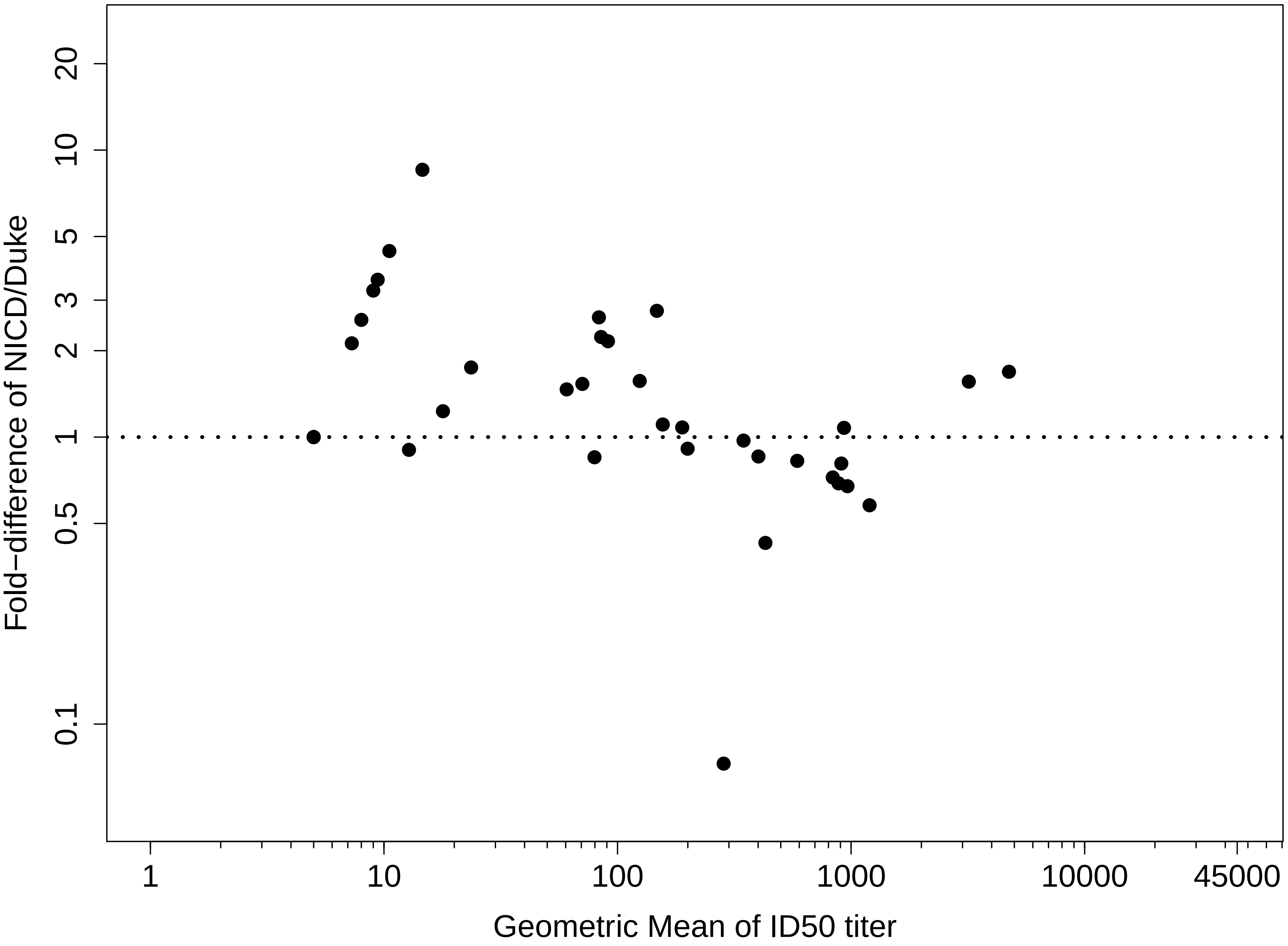

Figure 3d: Bland−Altman plot of ID50 titer for BA.4/5 at Months 1/2

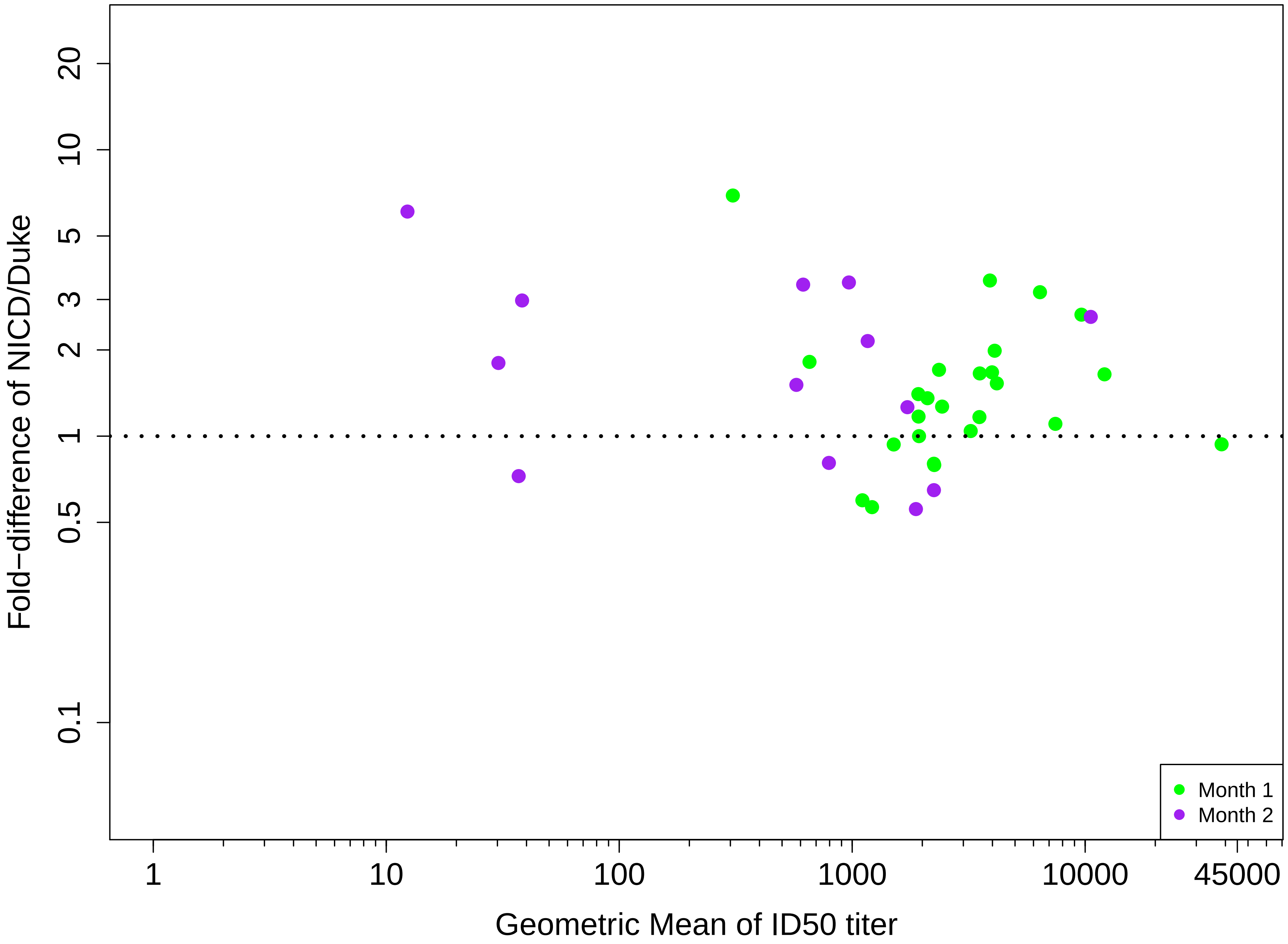

Figure 3e: Bland−Altman plot of ID80 titer for D614G at Month 0

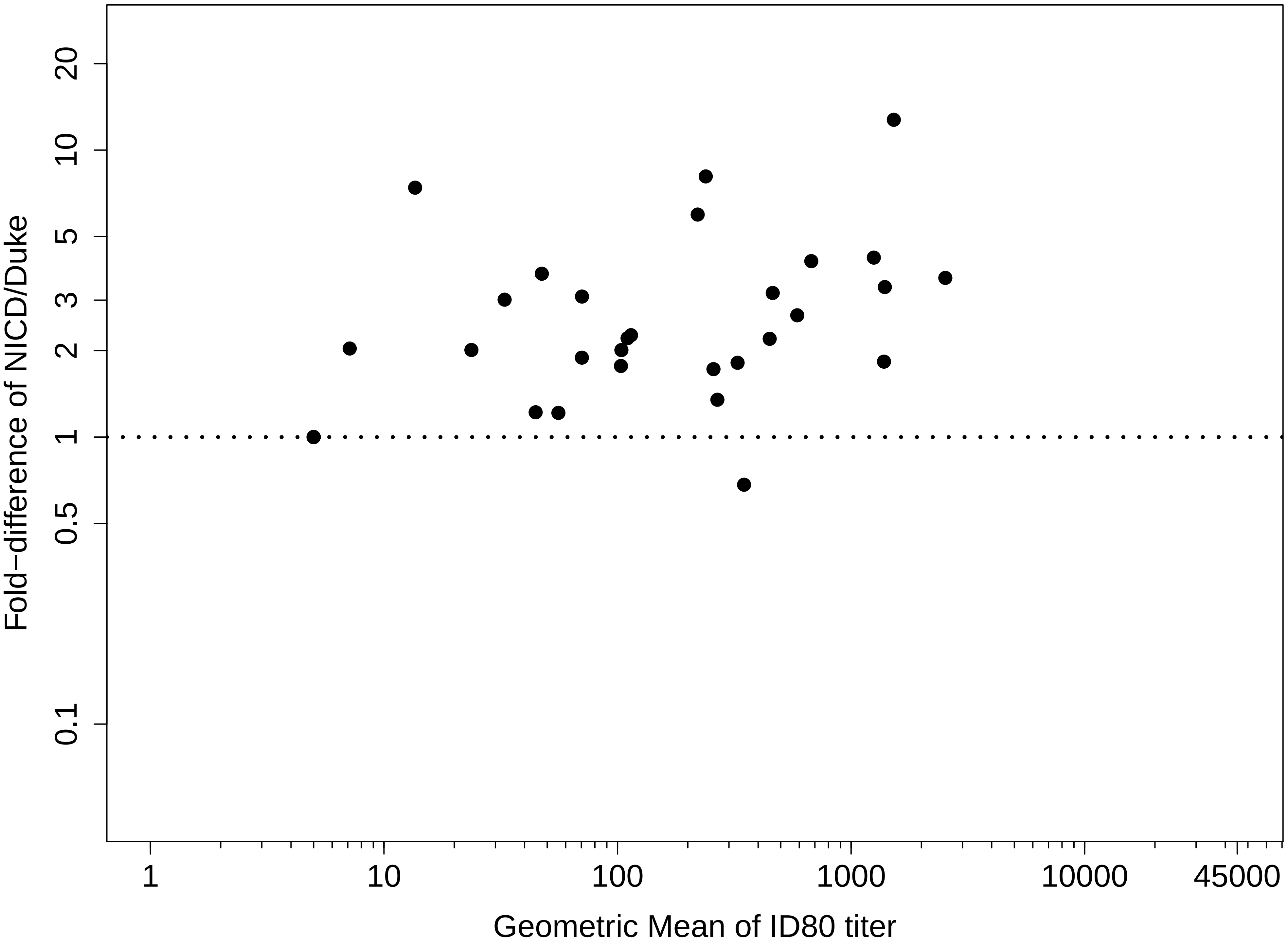

Figure 3f: Bland−Altman plot of ID80 titer for D614G at Months 1/2

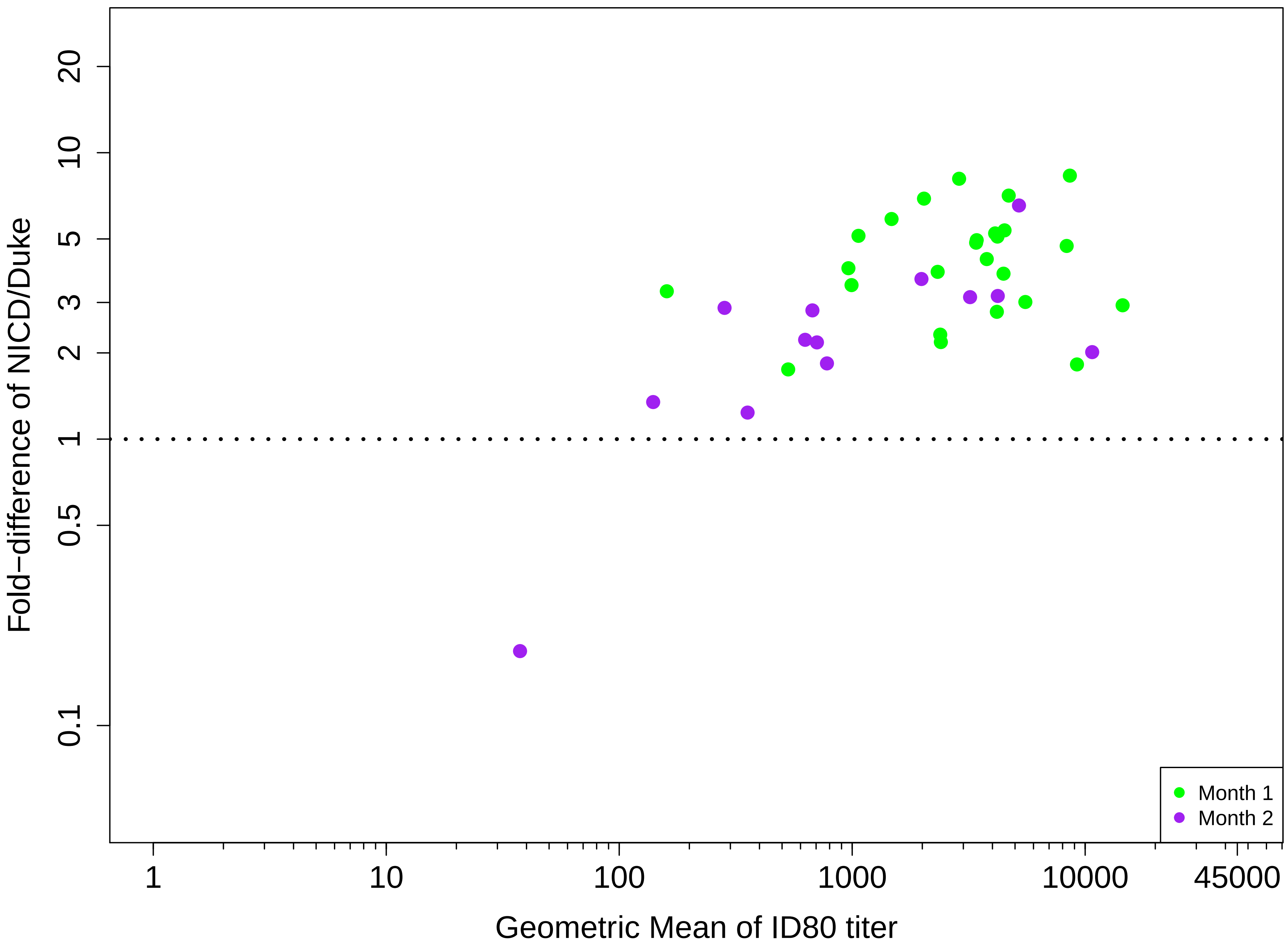

Figure 3g: Bland−Altman plot of ID80 titer for BA.4/5 at Month 0

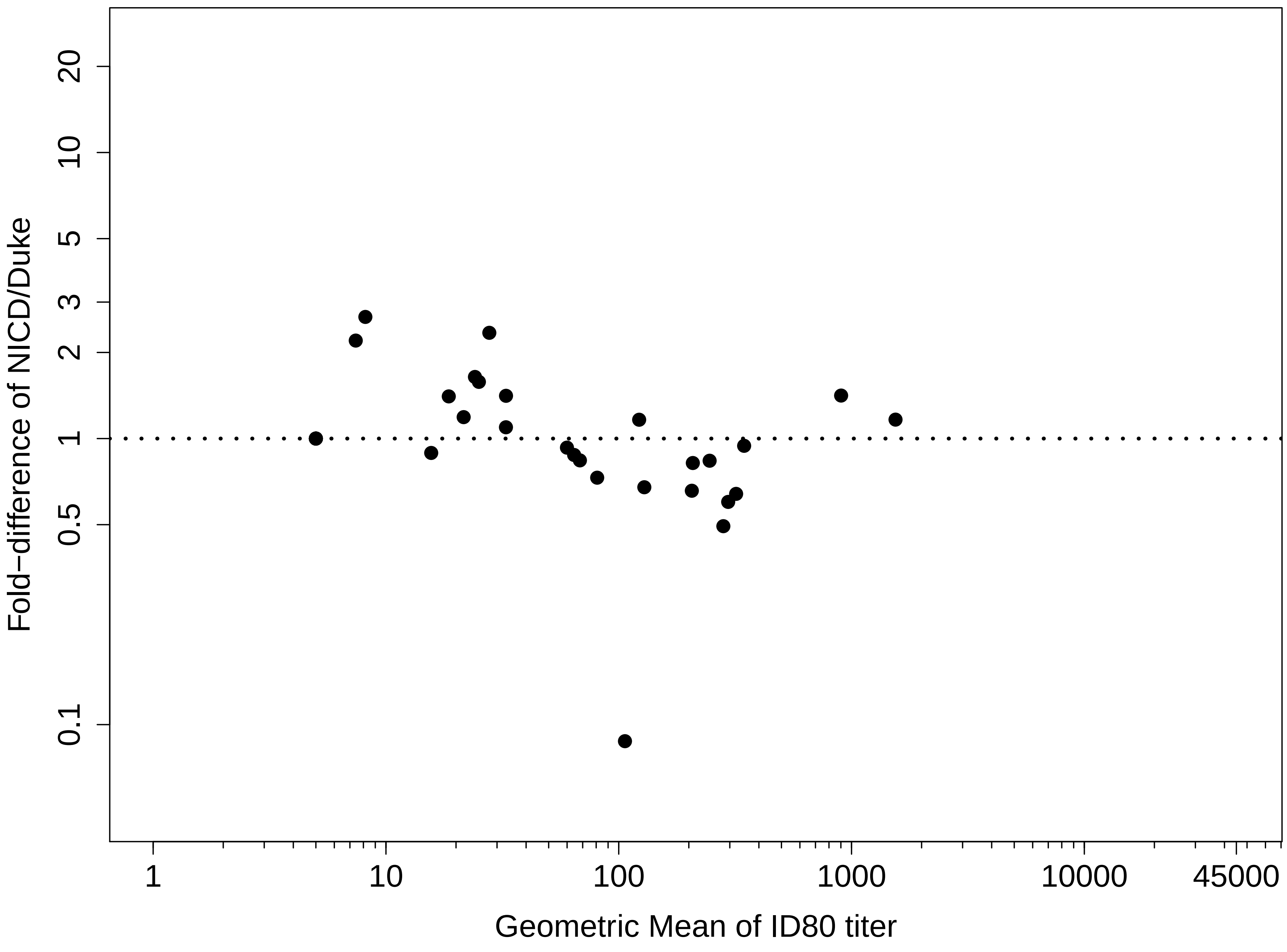

Figure 3h: Bland−Altman plot of ID80 titer for BA.4/5 at Months 1/2

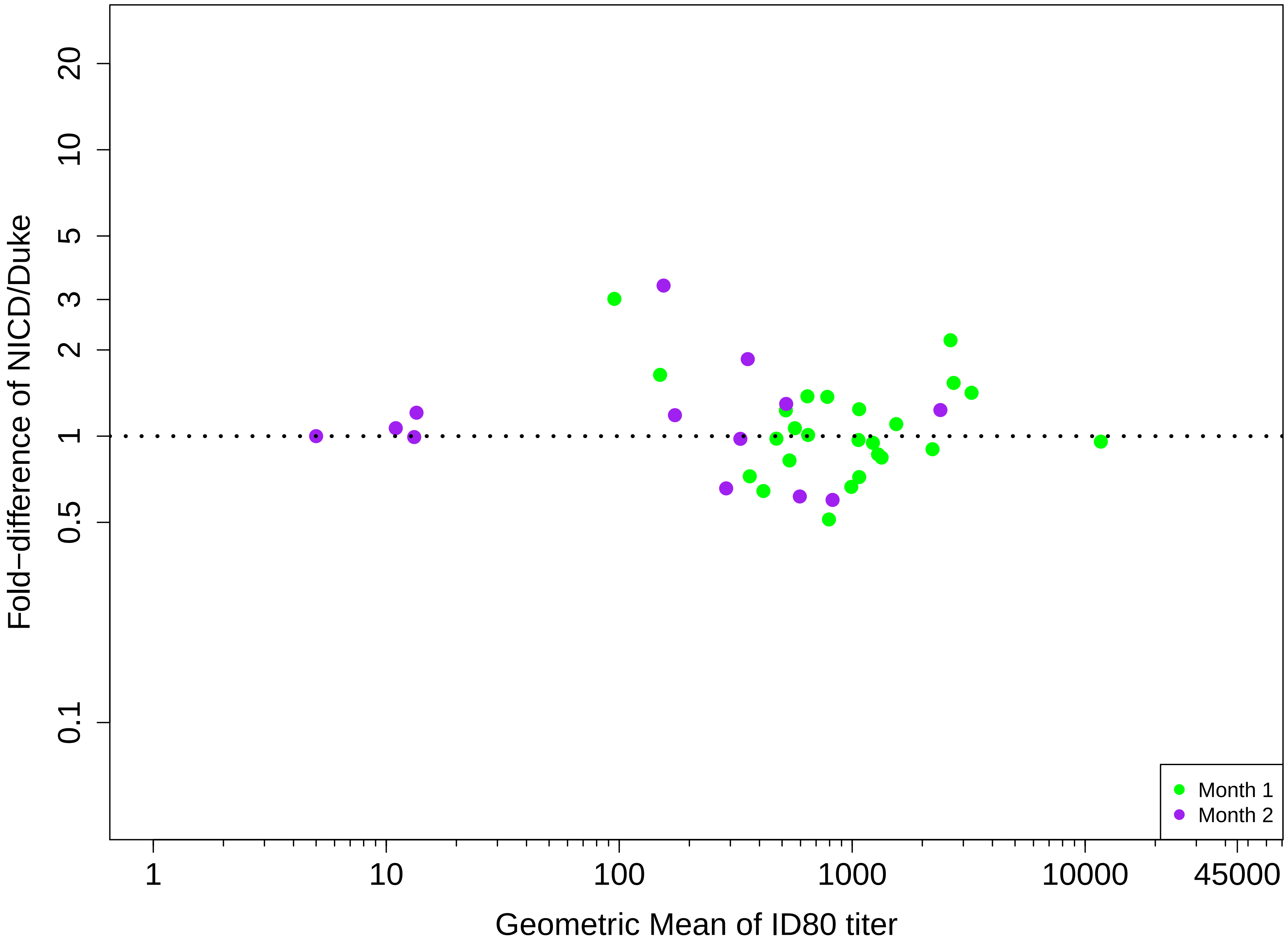

Supplement: Supplementary file 1 — Supplementary Information [file 41467_2025_63948_MOESM1_ESM.pdf]
