# Supplementary material for: Neutralizing and binding antibodies are a correlate of risk of COVID-19 in the CoVPN 3008 study in people with HIV
Source: Nat Commun. 2025 Oct 6;16:8876. doi: 10.1038/s41467-025-63948-4 (PMC12501024; doi:10.1038/s41467-025-63948-4)
Supplement: Supplementary file 3 — Reporting Summary [file 41467_2025_63948_MOESM3_ESM.pdf]

nature portfolio

Corresponding author(s): Georgia D. Tomaras and Peter B. Gilbert

Last updated by author(s): August 10, 2025

Reporting Summary

Nature Portfolio wishes to improve the reproducibility of the work that we publish. This form provides structure for consistency and transparency in reporting. For further information on Nature Portfolio policies, see our Editorial Policies and the Editorial Policy Checklist.

Statistics

For all statistical analyses, confirm that the following items are present in the figure legend, table legend, main text, or Methods section.

n/a	Confirmed	
☐	☒	The exact sample size (*n*) for each experimental group/condition, given as a discrete number and unit of measurement
☐	☒	A statement on whether measurements were taken from distinct samples or whether the same sample was measured repeatedly
☐	☒	The statistical test(s) used AND whether they are one- or two-sided<br>*Only common tests should be described solely by name; describe more complex techniques in the Methods section.*
☐	☒	A description of all covariates tested
☐	☒	A description of any assumptions or corrections, such as tests of normality and adjustment for multiple comparisons
☐	☒	A full description of the statistical parameters including central tendency (e.g. means) or other basic estimates (e.g. regression coefficient) AND variation (e.g. standard deviation) or associated estimates of uncertainty (e.g. confidence intervals)
☐	☒	For null hypothesis testing, the test statistic (e.g. *F*, *t*, *r*) with confidence intervals, effect sizes, degrees of freedom and *P* value noted<br>*Give P values as exact values whenever suitable.*
☒	☐	For Bayesian analysis, information on the choice of priors and Markov chain Monte Carlo settings
☒	☐	For hierarchical and complex designs, identification of the appropriate level for tests and full reporting of outcomes
☐	☒	Estimates of effect sizes (e.g. Cohen's *d*, Pearson's *r*), indicating how they were calculated

Our web collection on statistics for biologists contains articles on many of the points above.

Software and code

Policy information about availability of computer code

Data collection	VSV-based neutralizing antibody assay: Perkin Elmer 2030 workstation version 4 was used for acquisition.<br>Binding antibody assay: MESO SECTOR S 600 instrument using Methodical Mind Instrument Software was used for acquisition.
Data analysis	VSV-based neutralizing antibody assay: SCHARP’s Atlas portal (https://atlas.scharp.org/project/home/begin.view) was used for data analysis.<br>Binding antibody assay: MSD Discover Workbench 4.0 Analysis Software was used for data analysis.<br>Statistical analyses: All analyses were done reproducibly based on publicly available R scripts hosted on the GitHub collaborative platform at https://github.com/CoVPN/CoVPN3008/tree/master/Antibody_manucript.

For manuscripts utilizing custom algorithms or software that are central to the research but not yet described in published literature, software must be made available to editors and reviewers. We strongly encourage code deposition in a community repository (e.g. GitHub). See the Nature Portfolio guidelines for submitting code & software for further information.

Data

Policy information about availability of data

All manuscripts must include a data availability statement. This statement should provide the following information, where applicable:

- Accession codes, unique identifiers, or web links for publicly available datasets
- A description of any restrictions on data availability
- For clinical datasets or third party data, please ensure that the statement adheres to our policy

In accordance with the data sharing policies of the National Institute of Allergy and Infectious Diseases, permission to access data will have to be requested from the HVTN and Statistical Center for HIV/AIDS Research & Prevention (SCHARP). The trial dataset with data dictionary will be made available to appropriate academic parties with input from the investigator group subject to submission of a suitable study protocol and analysis plan. Requests should be directed to Peter Gilbert (pgilbert@fredhutch.org) and will be responded to within one month. Some of the findings of this study are based on Spike amino acid sequences obtained from 3,000 individual SARS-CoV-2 genome sequences available on GISAID up to December 20, 2024 (EPI_SET_241220es), and publicly accessible at https://doi.org/10.55876/gis8.241220es (Supplementary Table 4). Source Data pertaining to Figs. 3, 4, and 6 are provided with this paper.

Research involving human participants, their data, or biological material

Policy information about studies with human participants or human data. See also policy information about sex, gender (identity/presentation), and sexual orientation and race, ethnicity and racism.

Reporting on sex and gender

Cell lines:
The VSV-based neutralizing antibody assay uses Vero E6 cells, a cell line isolated from the kidney of an African green monkey. While the supplier does not provide information on sex of the African green monkey from which this line was derived, Osada et al. (DNA Res. 2014 Sep 28;21(6):673–683. doi: 10.1093/dnares/dsu029) performed a genomic analysis of Vero cells (Vero JCRB0111, Japanese Collection of Research Bioresources Cell Bank) that revealed that Vero cells are of female provenance. It is unknown whether alternative neutralization assays would provide different results if based on a cell line derived from the kidney of a male African green monkey.

Human research participants:
Information on sex was determined based on self-report. Participants could choose between the following options: Male, Female.
Sex was used as one of the baseline input variables considered in building the baseline risk score, and sex was adjusted for in analyses comparing antibody levels between the Hybrid and Vaccine Groups and between the PWH and PWoH groups.
Disaggregated sex data are not provided in the source data because consent has not been obtained for sharing of individual-level data.
The present study did not prespecify any sex-based analyses as immunogenicity of the mRNA-1273 vaccine did not vary with sex in our previous analyses of this vaccine and there has not been evidence that sex modifies correlates of risk of COVID-19 for this vaccine.
Supplementary Table 1 provides demographics on Per-protocol Serum Random Immunogenicity Subset participants in key pre-specified subgroups and overall.

Reporting on race, ethnicity, or other socially relevant groupings

Race was self-reported on the Demographics Case Report Form, where participants could select Black, White, Indian, Asian (non-Indian), Colored/Mixed, or Other.
These categories mapped to the US Government categories as follows:

CoVPN 3008 Race category	US Government Category (OMB)
Black	Black or African American
White	White
Indian	Asian
Asian (non-Indian)	Asian
Colored/Mixed	Multiple - More than one race / Mixed race
Other (specified)	Other Race (specified as provided)

"Multiple race / Other" pools participants who selected Colored/Mixed, Other, or more than one race option.
No participants reported American Indian/Alaska Native or Native Hawaiian/Other Pacific Islander.
Also, Ethnicity ("Hispanic or Latino" vs "Not Hispanic or Latino") was collected separately and was not altered by the race recode.
Supplementary Table 1 provides demographics on Per-protocol Serum Random Immunogenicity Subset participants in key pre-specified subgroups and overall.

Population characteristics

Supplementary Table 1 summarizes demographic variables in the Serum-RIS (Random Immunogenicity Subset), which represent the Hybrid [N=49 males (14 PWH, 35 PWoH), mean age 40.8 years and N=89 females (52 PWH, 37 PWoH), mean age 39.3 years] and Vaccine [N=36 males (16 PWH, 20 PWoH), mean age 40.0 years and N=25 females (21 PWH, 4 PWoH), mean age 42.5 years] Group populations for immunogenicity assessment.
PWH = people with HIV; PWoH = people without HIV

Recruitment

Participants were recruited from 47 sites across East and Southern Africa based on broad eligibility criteria requiring HIV or another comorbidity associated with increased risk of severe COVID-19. Willingness to be vaccinated and attend study visits may have selected for individuals more engaged with healthcare, potentially leading to overestimates of immune responses and protection. Conversely, limiting enrollment to individuals with underlying health conditions, such as HIV, could have

biased results in the opposite direction.

Ethics oversight

We have complied with all relevant ethical regulations. The trial was registered on ClinicalTrials.gov (NCT05168813) and conducted according to International Council for Harmonisation of Technical Requirements for Pharmaceuticals for Human Use, Good Clinical Practice guidelines. Written informed consent was obtained from all participants prior to enrolment. Each site followed its standard, approved procedures to determine reimbursement amounts for participants to cover travel, time, and inconvenience costs associated with study participation.

The CoVPN 3008 study protocol was approved by the following Research Ethics Committees (RECs) [listed as Clinical Research Site (CRS)/Institution Name — REC(s); Regulatory Body(ies)]: Gaborone CRS — Harvard Institutional Review Board (IRB) and Health Research and Development Committee; Botswana Medicines Regulatory Authority. Eswatini Prevention Center CRS — Columbia Human Research Protection Office IRB, Eswatini Health and Human Research Review Board; Medicine Regulatory Unit. Moi University Clinical Research Centre (CRC) — Moi University CRC: Institutional Research and Ethics Committee (IREC); Ministry of Health Pharmacy and Poisons Board and National Commission for Science, Technology and Innovation (NACOSTI). Kisumu CRS — Kenya Medical Research Institute (KEMRI) Scientific Ethics Review Unit; NACOSTI, Jaramogi Oginga Odinga Teaching & Referral Hospital (JOOTRH), County Administry of Health. Kombewa CRC — KEMRI Scientific Ethical Review Unit & Walter Reed Army Institute of Research; NACOSTI, County Administry of Health. Blantyre CRS — College of Medicine Research Ethics Committee, University of Malawi College of Medicine, Johns Hopkins School of Public Health IRB. Malawi CRS — University of North Carolina (UNC) Chapel Hill Office of Human Research Ethics, National Health Science Research Committee. UVRI-IAVI HIV Vaccine Program LTD. CRS — UVRI (Uganda Virus Research Institute Research and Ethics Committee) Ethics; Uganda National Council for Science and Technology (UNCST). Baylor-Uganda CRS — UVRI REC/IRC; UNCST. Joint Clinical Research Centre — UVRI REC; UNCST. MU-JHU Research Collaboration CRS — UVRI Ethics; UNCST. CFHRZ CRS — University of Zambia Biomedical Research Ethics Committee (UNZABREC); Zambia Medicines Regulatory Authority; National Health Research Authority. Matero Reference Clinic CRS / Centre for Infectious Disease Research in Zambia (CIDRZ) — UNZABREC; Zambia Medicines Regulatory Authority; National Health Research Authority. UNC Global Projects / Kamwala District Health Centre — UNZABREC; Zambia Medicines Regulatory Authority; National Health Research Authority. Zambia Emory HIV Research Project – Ndola CRS — UNZABREC; Zambia Medicines Regulatory Authority; National Health Research Authority. Zambia Emory HIV Research Project – Ndola CRS — UNZABREC; Zambia Medicines Regulatory Authority; National Health Research Authority. PHOENIX Pharma (Pty) Ltd — Pharma Ethics; South African Health Products Regulatory Authority (SAHPRA). Groote SchuurHIV CRS — University of Cape Town Human Research Ethics Committee (UCT HREC); SAHPRA. Task Central — Pharma Ethics; SAHPRA. Soweto - Kliptown CRS — Wits HREC; SAHPRA. FAM-CRU (Family Clinical Research Unit) — University of Stellenbosch Ethics Committee; SAHPRA. Josha Research CRS — Wits HREC; SAHPRA. Tembisa Clinic 4 — Wits HREC; SAHPRA. Qhakaza Mbokodo Research Clinic (QMRC) — Wits HREC; SAHPRA. Aurum Institute Klerksdorp CRS — Wits HREC; SAHPRA. Rustenburg CRS — Wits HREC; SAHPRA. Emavundleni CRS — UCT HREC; SAHPRA. Clinical HIV Research Unit (CHRU)/ Helen Joseph CRS — Wits HREC; SAHPRA. CAPRISA eThekwini Clinic — University of KwaZulu-Natal Biomedical Research Ethics Committee (UKZN BREC); SAHPRA. Masiphumelele Clincial Research Site (Masi) CRS — UCT HREC; SAHPRA. Soweto - Bara CRS — Wits HREC; SAHPRA. Tongaat CRS — South African Medical Research Council (SAMRC); SAHPRA. CAPRISA Vulindlela CRS — UKZN BREC; SAHPRA. Nelson Mandela Academic Clinical Research Unit CRS — Wits HREC; SAHPRA. MeCRU CRS — Sefako Makgatho University Research Ethics Committee (SMUREC); SAHPRA. University of Cape Town Lung CRS Institute — UCT HREC; SAHPRA. PHRU Matlosana CRS — Wits HREC; SAHPRA. Synergy Biomed Research Institute — Pharma Ethics; SAHPRA. Newtown Clinical Research — Pharma Ethics; SAHPRA. TASK Eden — Pharma Ethics; SAHPRA. Wits RHI Ward 21 CRS — Wits HREC; SAHPRA. Isipingo CRS — SAMRC; SAHPRA.

Note that full information on the approval of the study protocol must also be provided in the manuscript.

Field-specific reporting

Please select the one below that is the best fit for your research. If you are not sure, read the appropriate sections before making your selection.

☒ Life sciences ☐ Behavioural & social sciences ☐ Ecological, evolutionary & environmental sciences

For a reference copy of the document with all sections, see nature.com/documents/nr-reporting-summary-flat.pdf

Life sciences study design

All studies must disclose on these points even when the disclosure is negative.

Sample size

Table 4 in the USG COVID-19 Response Team / CoVPN Vaccine Efficacy Trial Immune Correlates Statistical Analysis Plan (https://doi.org/10.6084/m9.figshare.13198595.v13) provides minimum numbers of evaluable endpoints in a given population of interest to initiate different types of correlates analyses in that population, where these numbers generally range between 25 and 50. The HVTN 505 trial serves as a precedent where 25 evaluable vaccine recipient cases provided enough data to reasonably characterize correlates of risk for a preventive candidate HIV vaccine (Janes et al., 2017; Fong et al., 2018; Neidich et al., 2019; Gilbert et al., 2020b). In addition, simulation studies show that correlates analyses at 20 endpoints have notably lower precision.

For this CoVPN 3008 Part A antibody correlates study, all the correlates of risk analyses listed in Table 10 of the SAP were planned to be conducted, as well as the controlled effects analyses of correlates of protection.

Data exclusions

Table 1 in the SAP lists reasons for exclusion from the per-protocol correlates cohorts, which included
- Discordant numbers of primary vaccinations planned vs. received (Analysis Group 2-2 or 4-2 in the primary paper by Garrett et al.).
- Early termination/drop out by peak
- CDC COVID-19 endpoint by 6 days post peak
- >= 4-fold increase in anti N IgG concentration from M0 to peak
- Out of window peak visit (<15 or > 42 days)

The rationale for these exclusions is that including these participants may complicate interpretation of the results and/or hinder the detection

	of correlates.<br>Moreover, anti-N markers at Peak (and fold-rise from M0 to Peak) were excluded because the mRNA-1273 vaccine did not contain the N protein. These exclusions were also prespecified in the SAP (Section 5.5).
Replication	All statistical analyses were implemented in automated and reproducible press-button fashion, based on publicly available R scripts hosted on the GitHub collaborative platform at https://github.com/CoVPN/CoVPN3008/tree/master/Antibody_manucript.<br>All results in the paper can be replicated using the provided R scripts and an analysis-ready dataset can be requested from the HVTN.
Randomization	Part A of CoVPN 3008, which was studied in this work, was an observational study, not a randomized trial.<br>Participants were allocated to receive one or two doses of 100 mcg of mRNA-1273 vaccine depending on testing SARS-CoV-2 anti-Spike positive or negative as assessed by a point-of-care SARS-CoV-2 anti-spike serology test (POC anti-S) (Assure Ecotest, Assure Tech, Hangzhou, China) taken at baseline.<br>The baseline factors adjusted for in immune correlates analyses are pre-specified and detailed in Section 9 of the SAP. In sum, as in Garrett et al., the following baseline variables are adjusted for in the immune correlates analyses of antibody markers: region of enrollment (South Africa versus any other African country), period of enrollment (< 3 months, 3-6 months, > 6 months post first person enrolled), HIV status, TB status (evidence of prior or active tuberculosis at baseline), and<br>baseline risk score.<br>In addition, analyses comparing antibody levels between the Hybrid and Vaccine Groups and between the PWH and PWoH groups (within each of the Hybrid and Vaccine groups) adjusted for whether age exceeded 40 and the four categories (male, female) × (BMI <= 25, BMI > 25); where the Hybrid vs. Vaccine group analyses also adjusted for PWH status.
Blinding	Neither participants nor investigators were blinded to treatment assignment (one-dose vs two-dose mRNA-1273 vaccine) in Part A of CoVPN 3008. This is because participants were assigned to the appropriate treatment group depending on testing SARS-CoV-2 anti-Spike positive or negative at baseline (not randomized).<br>Lab technicians, when measuring the marker levels, were blinded to case/non-case status and time point of sample collection. Statisticians were blinded to case/non-case status for analyses that characterized immunogenicity and were not blinded to case/non-case status for analyses that assessed correlates of risk. Inferential statistical analysis was conducted in an automated/press-button fashion, with the inferences valid based on the pre-specification of inferences and the reproducibility of the computer code.

Reporting for specific materials, systems and methods

We require information from authors about some types of materials, experimental systems and methods used in many studies. Here, indicate whether each material, system or method listed is relevant to your study. If you are not sure if a list item applies to your research, read the appropriate section before selecting a response.

Materials & experimental systems

n/a	Involved in the study
☐	☒ Antibodies
☐	☒ Eukaryotic cell lines
☒	☐ Palaeontology and archaeology
☒	☐ Animals and other organisms
☐	☒ Clinical data
☒	☐ Dual use research of concern
☒	☐ Plants

Methods

n/a	Involved in the study
☒	☐ ChIP-seq
☒	☐ Flow cytometry
☒	☐ MRI-based neuroimaging

Antibodies

Antibodies used	SULFO-TAG Anti-Human IgG Antibody, MSD, Catalog Number: D21ADF-3. Was used diluted (1X) in MSD Diluent 100 solution.
Validation	Not applicable.

Eukaryotic cell lines

Policy information about cell lines and Sex and Gender in Research

Cell line source(s)	Vero E6 cells (American Type Culture Collection (ATCC) cat# CRL-1586
Authentication	There was no formal authentication of the cells but the validation of the assay, conducted after the assay transfer, yielded equivalent neutralization results with Nexelis, the developers of the assay.
Mycoplasma contamination	Cells were tested for Mycoplasma quarterly using Lonza's MycoAlert® kit and results were negative for mycoplasma.
Commonly misidentified lines<br>(See ICLAC register)	Not applicable.

Clinical data

Policy information about clinical studies

All manuscripts should comply with the ICMJE guidelines for publication of clinical research and a completed CONSORT checklist must be included with all submissions.

Clinical trial registration	CoVPN 3008 is registered on ClinicalTrials.gov (NCT05168813). The present study considers Part A of CoVPN 3008, which was an observational study (not a randomized trial).
Study protocol	The full trial protocol is available with Garrett et al. 2025 eClinicalMedicine, and can be accessed at the following link: https://pmc.ncbi.nlm.nih.gov/articles/PMC11788791/#appsec1
Data collection	Participants were enrolled participants at 47 clinical research sites in seven East and Southern African countries: Botswana, Eswatini, Kenya, Malawi, South Africa, Uganda, and Zambia. Study participants received the first vaccination between December 2, 2021, and September 9, 2022 (from Garrett et al.).<br>Garrett et al. also describe surveillance for occurrence of COVID-19, which includes nasal swabs for SARS-CoV-2 NAAT before each vaccination and at month 6, self-monitoring for prespecified COVID-19 symptoms at home, and site personnel contacting participants every two weeks to assess for symptoms. COVID-19 symptoms triggered a clinic visit for further assessment and nasal swab testing.
Outcomes	The primary endpoints were the first occurrence of COVID-19 or severe COVID-19 with onset at least 1 day after the enrolment vaccination until the month 6 visit among PWH. Using CDC criteria, COVID-19 was defined as one NAAT-positive nasal swab within 14 days of the onset of at least one systemic symptom (fever ≥38 °C, chills, myalgia, headache, sore throat, new loss of taste or smell), or at least one respiratory sign/symptom (cough, shortness of breath or difficulty breathing), or clinical or radiographical evidence of pneumonia. (from Garrett et al.).

Plants

Seed stocks	Not applicable.
Novel plant genotypes	Not applicable.
Authentication	Not applicable.
